# Identifying genetic variants that affect viability in large cohorts

**DOI:** 10.1371/journal.pbio.2002458

**Published:** 2017-09-05

**Authors:** Hakhamanesh Mostafavi, Tomaz Berisa, Felix R. Day, John R. B. Perry, Molly Przeworski, Joseph K. Pickrell

**Affiliations:** 1 Department of Biological Sciences, Columbia University, New York, New York, United States of America; 2 New York Genome Center, New York, New York, United States of America; 3 MRC Epidemiology Unit, Institute of Metabolic Science, University of Cambridge, Cambridge, United Kingdom; 4 Department of Systems Biology, Columbia University, New York, New York, United States of America; The Institute of Science and Technology Austria, AUSTRIA

## Abstract

A number of open questions in human evolutionary genetics would become tractable if we were able to directly measure evolutionary fitness. As a step towards this goal, we developed a method to examine whether individual genetic variants, or sets of genetic variants, currently influence viability. The approach consists in testing whether the frequency of an allele varies across ages, accounting for variation in ancestry. We applied it to the Genetic Epidemiology Research on Adult Health and Aging (GERA) cohort and to the parents of participants in the UK Biobank. Across the genome, we found only a few common variants with large effects on age-specific mortality: tagging the *APOE* ε4 allele and near *CHRNA3*. These results suggest that when large, even late-onset effects are kept at low frequency by purifying selection. Testing viability effects of sets of genetic variants that jointly influence 1 of 42 traits, we detected a number of strong signals. In participants of the UK Biobank of British ancestry, we found that variants that delay puberty timing are associated with a longer parental life span (*P*~6.2 × 10^−6^ for fathers and *P*~2.0 × 10^−3^ for mothers), consistent with epidemiological studies. Similarly, variants associated with later age at first birth are associated with a longer maternal life span (*P*~1.4 × 10^−3^). Signals are also observed for variants influencing cholesterol levels, risk of coronary artery disease (CAD), body mass index, as well as risk of asthma. These signals exhibit consistent effects in the GERA cohort and among participants of the UK Biobank of non-British ancestry. We also found marked differences between males and females, most notably at the *CHRNA3* locus, and variants associated with risk of CAD and cholesterol levels. Beyond our findings, the analysis serves as a proof of principle for how upcoming biomedical data sets can be used to learn about selection effects in contemporary humans.

## Author summary

Our global understanding of adaptation in humans is limited to indirect statistical inferences from patterns of genetic variation, which are sensitive to past selection pressures. We introduced a method that allowed us to directly observe ongoing selection in humans by identifying genetic variants that affect survival to a given age (i.e., viability selection). We applied our approach to the GERA cohort and parents of the UK Biobank participants. We found viability effects of variants near the *APOE* and *CHRNA3* genes, which are associated with the risk of Alzheimer disease and smoking behavior, respectively. We also tested for the joint effect of sets of genetic variants that influence quantitative traits. We uncovered an association between longer life span and genetic variants that delay puberty timing and age at first birth. We also detected detrimental effects of higher genetically predicted cholesterol levels, body mass index, risk of coronary artery disease (CAD), and risk of asthma on survival. Some of the observed effects differ between males and females, most notably those at the *CHRNA3* gene and variants associated with risk of CAD and cholesterol levels. Beyond this application, our analysis shows how large biomedical data sets can be used to study natural selection in humans.

## Introduction

A number of central questions in evolutionary genetics remain open, in particular for humans. Which types of variants affect fitness? Which components of fitness do they affect? What is the relative importance of directional and balancing selection in shaping genetic variation? Part of the difficulty is that our understanding of selection pressures acting on the human genome is based either on experiments in fairly distantly related species or cell lines or on indirect statistical inferences from patterns of genetic variation [1–3].

The statistical inferences rely on patterns of genetic variation in present-day samples (or, very recently, in ancient samples [4]) to identify regions of the genome that appear to carry the footprint of positive selection [2]. For example, a commonly used class of methods asks whether rates of nonsynonymous substitutions between humans and other species are higher than expected from putatively neutral sites in order to detect recurrent changes to the same protein [5]. Another class instead relies on polymorphism data and looks for various footprints of adaptation involving single changes of large effect [6]. These approaches detect adaptation over different timescales and, likely as a result, suggest quite distinct pictures of human adaptation [1]. For example, approaches that are sensitive to selective pressures acting over millions of years have identified individual chemosensory and immune-related genes (e.g., [7]). In contrast, approaches that are most sensitive to selective pressures active over thousands or tens of thousands of years have revealed strong selective pressures on individual genes that influence human pigmentation (e.g., [8–10]), diet [11–13], as well as sets of variants that shape height [14–16]. Even more recent still, studies of contemporary populations have suggested that natural selection has influenced life-history traits like age at first childbirth as well as educational attainment over the course of the last century [17–23].

Because these approaches are designed (either explicitly or implicitly) to be sensitive to a particular mode of adaptation, they provide a partial and potentially biased picture of what variants in the genome are under selection. In particular, most have much higher power to adaptations that involve strongly beneficial alleles that were rare in the population when first favored and will tend to miss selection on standing variation or adaptation involving many loci with small beneficial effects (e.g., [24–27]). Moreover, even where these methods identify a beneficial allele, they are not informative about the components of fitness that are affected or about possible fitness trade-offs between sexes or across ages.

In line with Lewontin’s proposal to track age-specific mortality and fertility of hundreds of thousands of individuals [28], we sought a more direct and, in principle, comprehensive way to study adaptation in humans, focusing on current viability selection. Similar to the approach that Allison took in comparing frequencies of the sickle cell allele in newborns and adults living in malarial environments [29], we aimed to directly observe the effects of genotypes on survival by taking advantage of the recent availability of genotypes from large cohorts of individuals of different ages. Specifically, we tested for differences in the frequency of an allele across individuals of different ages, controlling for changes in ancestry and possible batch effects. This approach resembles a genome-wide association study (GWAS) for longevity yet does not focus on an end point (e.g., survival to an old age) but on any shift in allele frequencies with age. Thus, it allows the identification of possible nonmonotonic effects at different ages or sex differences. Any genetic variant that affects survival by definition has a fitness cost, even if the cost is too small to be effectively selected against (depending on the effective population size, the age structure of the population, and the age at which the variant exerts its effects [30]). Of course, a genetic variant can influence fitness without influencing survival through effects on reproduction or inclusive fitness. Our approach is therefore considering only 1 of the components of fitness that are likely important for human adaptation.

As a proof of principle, we applied our approach to 2 recent data sets: to 57,696 individuals of European ancestry from the Genetic Epidemiology Research on Adult Health and Aging (GERA) cohort [31,32] and, by proxy [33–35], to the parents of 117,648 individuals of British ancestry surveyed as part of the UK Biobank [36]. We did so for individual genetic variants then jointly for sets of variants previously found to influence 1 of 42 polygenic traits [37–40].

## Results

### A method for testing for differences in allele frequencies across age bins

If a genetic variant does not influence viability, its frequency should be the same in individuals of all ages. We therefore tested for changes in allele frequency across individuals of different ages, while accounting for systematic differences in the ancestry of individuals of different ages (for example, due to migration patterns over decades) and genotyping batch effects. We used a logistic regression model in which we regressed each individual’s genotype on their age bin, their ancestry as determined by principal component analysis (PCA) (S1 Fig), and the batch in which they were genotyped (see Materials and methods for details). In this model, we treated age bin as a categorical variable; this approach allowed us to test for a relationship between age and the frequency of an allele regardless of the functional form of this relationship. We also tested a model with an interaction between age and sex to assess whether a variant affects survival differently in the 2 sexes.

**Fig 1:**
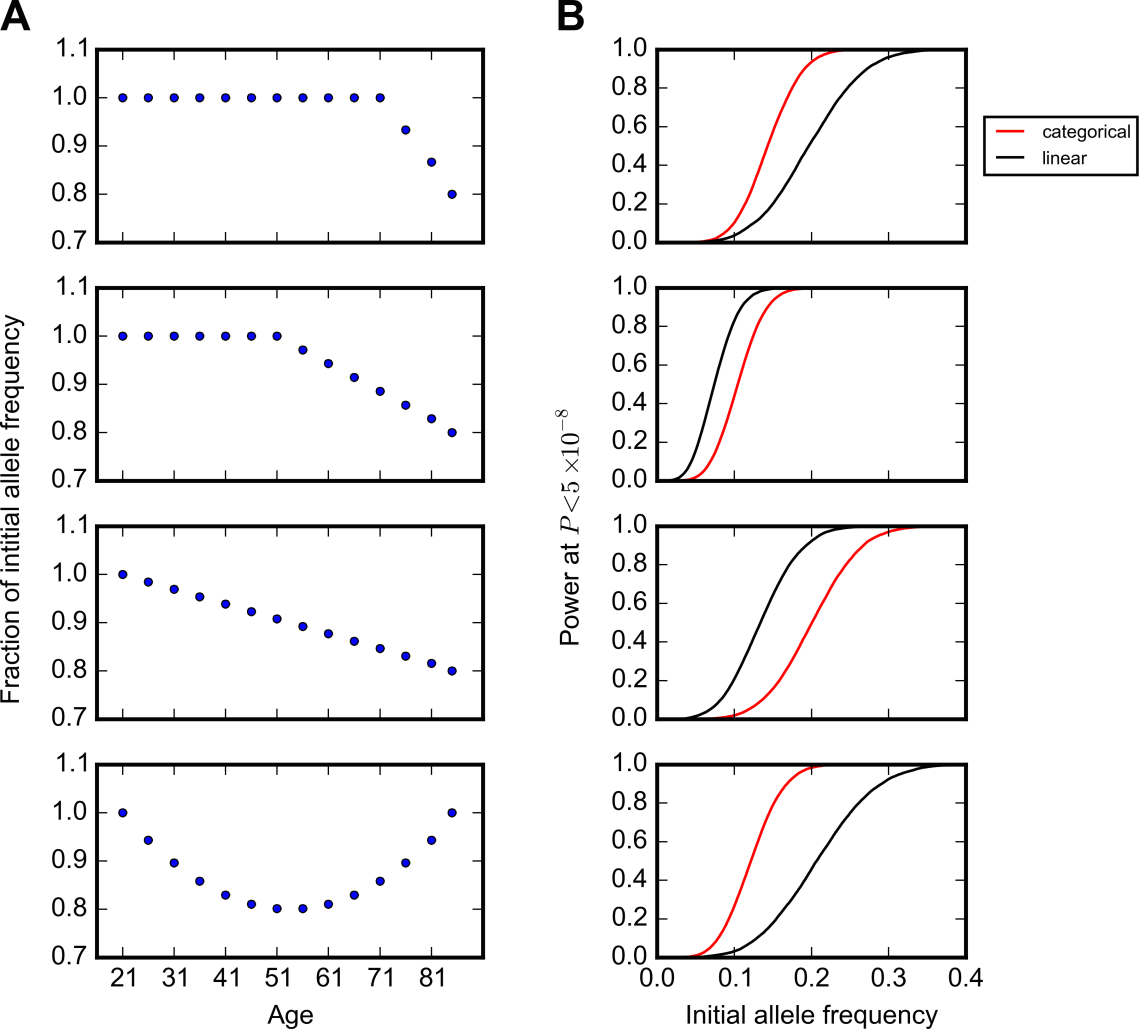
Power of the model to detect changes in allele frequency with age. (A) Trends in allele frequency with age considered in simulations. The y-axis indicates the allele frequency standardized to the frequency in the first age bin. (B) Power to detect the trends in (A) at *P* < 5 × 10^−8^, given the sample size per age bin in the Genetic Epidemiology Research on Adult Health and Aging (GERA) cohort (S2 Fig and total sample size of 57,696). Shown are results using models with age treated as a categorical (red) or an ordinal (black) variable, assuming no change in population structure and batch effects across age bins. The curves show simulation results sweeping allele frequency values with an increment value of 0.001 (1,000 simulations for each allele frequency) smoothed using a Savitzky-Golay filter in the SciPy package [43].

We first evaluated the power of this method using simulations. We considered 3 possible trends in allele frequency with age: (i) a constant frequency up to a given age followed by a steady decrease, i.e., a variant that affects survival after a given age (e.g., variants contributing to late-onset disorders), (ii) a steady decrease across all ages for a variant with detrimental effect throughout life, and (iii) a U-shaped pattern in which the allele frequency decreases to a given age but then increases, reflecting trade-offs in the effects at young and old ages, as hypothesized by the antagonistic pleiotropy theory of aging [41] or as may be seen if there are protective alleles that buffer the effect of risk alleles late in life [42] (Fig 1). In all simulations, we used sample sizes and age distributions that matched the GERA cohort (S2 Fig). For simplicity, we also assumed no population structure or batch effects across age bins (see Materials and methods). For all trends, we set a maximum of 20% change in the allele frequency from the value in the first age bin (Fig 1).

Because of the age distribution of individuals in the GERA cohort (S2 Fig), our power to detect the trend is greater when most of the change in allele frequency occurs in middle age (Fig 1). For example, for an allele with an initial allele frequency of 0.15 that begins to decrease in frequency among individuals at age 20, age 50, or age 70 years, there is around 20%, 90%, and 60% power, respectively, to detect the trend at *P* < 5 × 10^−8^, the commonly used criterion for genome-wide significance [44]. We also experimented with a version of the model in which the age bin is treated as an ordinal variable; as expected, this model is more powerful if there is in fact a linear relationship between age and allele frequency. Because we do not know the functional form of the relationship between age and allele frequency a priori in most cases, we used the categorical model for all analyses unless otherwise noted.

In the UK Biobank, all individuals were 45–69 years old at enrollment, so the age range of the participants is restricted and our method has low power. However, the UK Biobank participants reported the survival status of their parents: age of the parents if alive or age at which their parents died; following recent studies [33–35], we therefore used these values (when reported) instead in our model. In this situation, we are testing for correlations between an allele frequency and father’s or mother’s age (if alive) or age at death (if deceased). This approach obviously comes with the caveat that children inherit only half of their genome from each parent and so power is reduced (e.g., [45]). Furthermore, the patterns expected when considering individuals who have died differ subtly from those generated among surviving individuals. Notably, when an allele begins to decline in frequency starting at a given age (Fig 1A), there should be an increase in the allele frequency among individuals who died at that age followed by a decline in frequency, rather than the steady decrease expected among surviving individuals (S3 Fig, see Materials and methods for details). In a first analysis, we therefore focused on the majority of participants who reported father’s or mother’s age at death, 88,595 and 71,783 individuals, respectively. We compared the results of this approach with the results of a Cox proportional hazards model [46], which allowed us to include individuals who reported their parents to be alive but has the disadvantage of assuming fixed effects across all ages.

We further adapted this model to allow us to test for changes in frequency at sets of genetic variants jointly. Many phenotypes of interest, from complex disease risk to anthropomorphic and life-history traits such as age at menarche, are polygenic [47,48]. If a polygenic trait has an effect on fitness, either directly or indirectly (i.e., through pleiotropic effects), the individual loci that influence the trait may be too subtle in their survival effects to be detectable with current sample sizes. We therefore investigated whether there is a shift across ages in sets of genetic variants that were identified as influencing a trait in GWASs (S1 Table). Specifically, for a given trait, we calculated a polygenic score for each individual based on trait effect sizes of single variants previously estimated in GWASs and then tested whether the scores vary significantly across 5-year age bins (see Materials and methods for details). These scores are calculated under an additive model, which appears to provide a good fit to GWAS data [49].

If a polygenic trait is under stabilizing selection (e.g., human birth weight [50]), i.e., an intermediate polygenic score is optimal, no change in the mean value of polygenic scores across different ages is expected. However, if extreme values of a trait are associated with lower chance of survival, the spread of the polygenic scores should decrease with age. To consider this possibility, we tested whether the squared difference of the polygenic scores from the mean varies significantly with age (see Materials and methods for details).

### Testing for changes in allele frequency at individual genetic variants

We first applied the method to the GERA cohort using 8,868,517 filtered genotyped and imputed autosomal biallelic single-nucleotide polymorphisms (SNPs) and indels. We focused on a subset of 57,696 filtered individuals who we confirmed to be of European ancestry by PCA (see Materials and methods, S4 and S5 Figs). The ages of these individuals were reported in bins of 5-year intervals (distribution shown in S2 Fig). We tested for significant changes in allele frequencies across these bins. For each variant, we obtained a *P* value comparing a model in which the allele frequency changes with age to a null model. No inflation was detected in the quantile-quantile plot (S6A Fig), indicating that, for common variants at least, our control for population structure (and other potential confounders) is sufficient. To illustrate this point, we looked at the lactose intolerance-linked SNP rs4988235 within the *LCT* locus, which is among the most differentiated variants across European populations [11]; the trend in the expected allele frequency based on the null model (i.e., accounting for confounding batch effects and changes in ancestry) tracks the observed trend quite well (S7 Fig).

**Fig 2:**
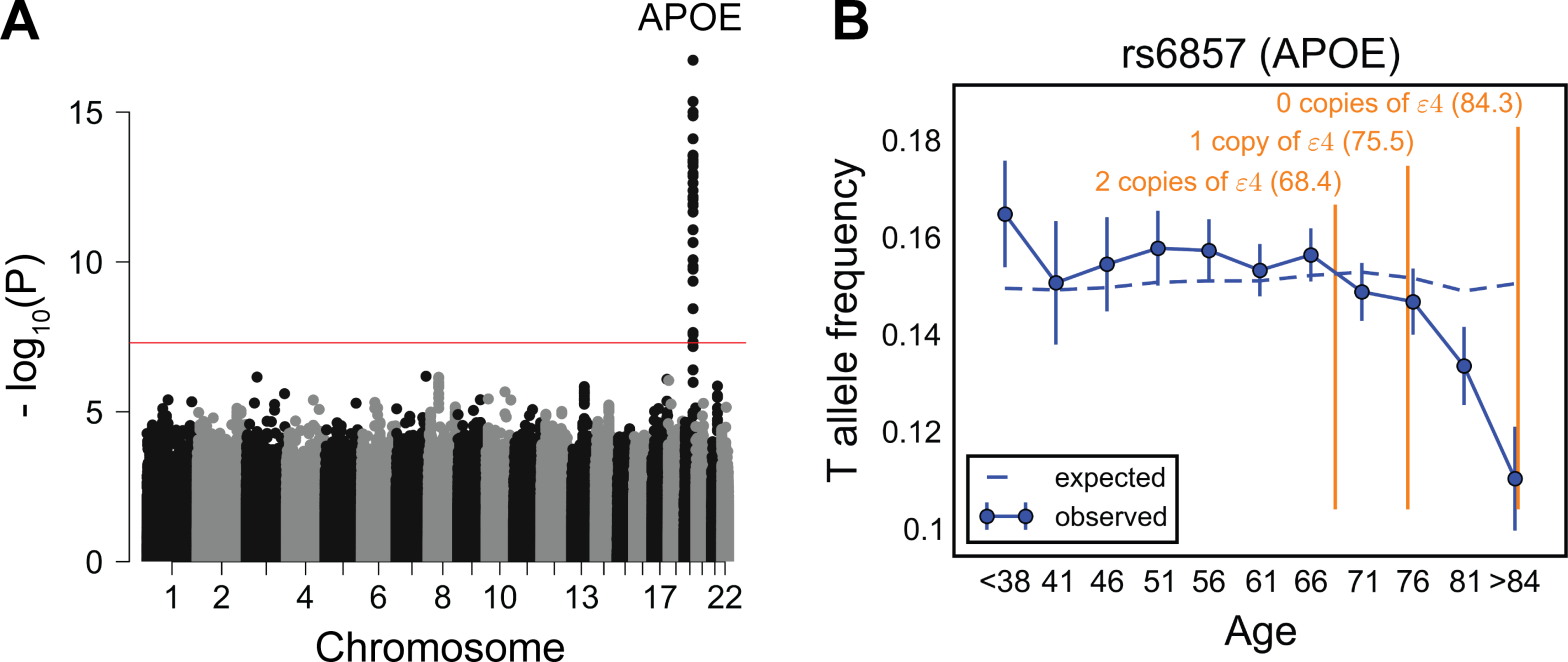
Testing for the influence of single genetic variants on age-specific mortality in the Genetic Epidemiology Research on Adult Health and Aging (GERA) cohort. (A) Manhattan plot of *P* values for the change in allele frequency with age. The red line marks the *P* = 5 × 10^−8^ threshold. (B) Allele frequency trajectory of rs6857, a tag SNP for the *APOE ε*4 allele, with age. Data points are the frequencies of the risk allele within 5-year interval age bins (± 2 SE), with the center of the bin indicated on the x-axis (except for the first and the last points). Bins with ages below 38 years are merged into 1 bin because of the relatively small sample sizes. The dashed line shows the expected frequency based on the null model, accounting for confounding batch effects and changes in ancestry (see Materials and methods). In orange are the mean ages at onset of Alzheimer disease for carriers of 0, 1, or 2 copies of the *APOE ε*4 allele [53]. See S1 Data for underlying data.

By our approach, all variants that reached genome-wide significance (*P* < 5 × 10^−8^) reside on chromosome 19 near the *APOE* gene (Fig 2A and S8 Fig). This locus has previously been associated with longevity in multiple studies [51,52]. The *APOE* ε4 allele is known to increase the risk of late-onset Alzheimer disease (AD) as well as of cardiovascular diseases [53,54]. We observe a monotonic decrease in the frequency of the T allele of the ε4 tag SNP rs6857 (C, protective allele; T, risk allele) beyond the age of 70 years old (Fig 2B). This trend is observed for both the heterozygous and homozygous risk variants (S9 Fig) and for both males and females (S10 Fig). No variant reaches genome-wide significance testing for age by sex interactions (quantile-quantile plot shown in S6B Fig).

We further investigated the trends in frequency with age for the other 2 major *APOE* alleles defined by rs7412 and rs429358 SNPs: ε2 (rs7412-T, rs429358-T) and ε3 (rs7412-C, rs429358-T), while ε4 is known by rs7412-C and rs429358-C alleles. Unlike the ε4 allele, ε2 allele carriers are suggested to be at lower risk of AD, cardiovascular disease, and mortality relative to the ε3 carriers [51,55]. We focused on a subset of 38,703 individuals with unambiguous counts of each *APOE* allele. There is a significant change in the frequency of the ε4 allele with age in this subset (*P*~6.0 × 10^−12^), similar to the trend observed for the tag SNP rs6857 (S11 Fig). The ε3 allele shows the reverse trend, with a significant, monotonic increase in frequency beyond the age of 70 years old (*P*~1.7 × 10^−8^) (S11 Fig). The enrichment of the ε3 allele in elderly individuals can be explained by the corresponding depletion of the ε4 allele and does not necessarily imply an independent, protective effect of the ε3 allele. The frequency of the ε2 allele does not change significantly with age (*P*~0.21), possibly reflecting low power given its allele frequency of approximately 0.06 (S11 Fig).

We considered the possibility that some unobserved confounding variable was driving the strength of this signal at *APOE*. Since there are 2 genotyped SNPs with signals similar to rs6857 within the locus, genotyping error seems unlikely to be driving the pattern (S8 Fig). Another concern might be a form of ascertainment bias, in which individuals with AD are underrepresented in the Kaiser Permanente Medical Care Plan. However, there is no correlation in these data between the amount of time that an individual has been enrolled in this plan and the individual’s *APOE* genotype (S12 Fig). These observations, along with previously reported associations at this locus, argue that the allele frequency trends in Fig 2B are driven by effects of *APOE* genotype on mortality (or severe disability). Moreover, the effects that we identified are concordant with epidemiological data on the mean age at onset of AD, given 0 to 2 copies of *APOE* ε4 allele [53]. This case not only serves as a positive control for our approach, it illustrates the resolution that it provides about age effects of genetic variants.

We estimated that we have about 93% power to detect the trend in allele frequency with age as observed for rs6857 (at a genome-wide significance level, see Materials and methods). Using both versions of the model treating age bin as a categorical or an ordinal variable, we have similar power to detect other potential trends considered in Fig 1 for variants as common as rs6857 and with similar magnitude of effect on survival. Yet across the genome, only *APOE* variants show a significant change in allele frequency with age for both versions of the model (Fig 2 and S13 Fig). Thus, our finding only *APOE* ε4 allele indicates that there are few or no other common variants in the genome with an effect on survival as strong as is seen in the *APOE* region.

We then turned to the UK Biobank data set. We applied our method to individuals of British ancestry whose data passed our filters; of these, 88,595 had death information available for their father and 71,783 for their mother. We analyzed 590,437 genotyped autosomal variants, applying similar quality control (QC) measures as with the GERA data set (see Materials and methods). We tested for significant changes in allele frequencies with father’s age at death and mother’s age at death stratified in eight 5-year interval bins. As in the GERA data set, no inflation was detected in the quantile-quantile plots (S14 Fig).

**Fig 3:**
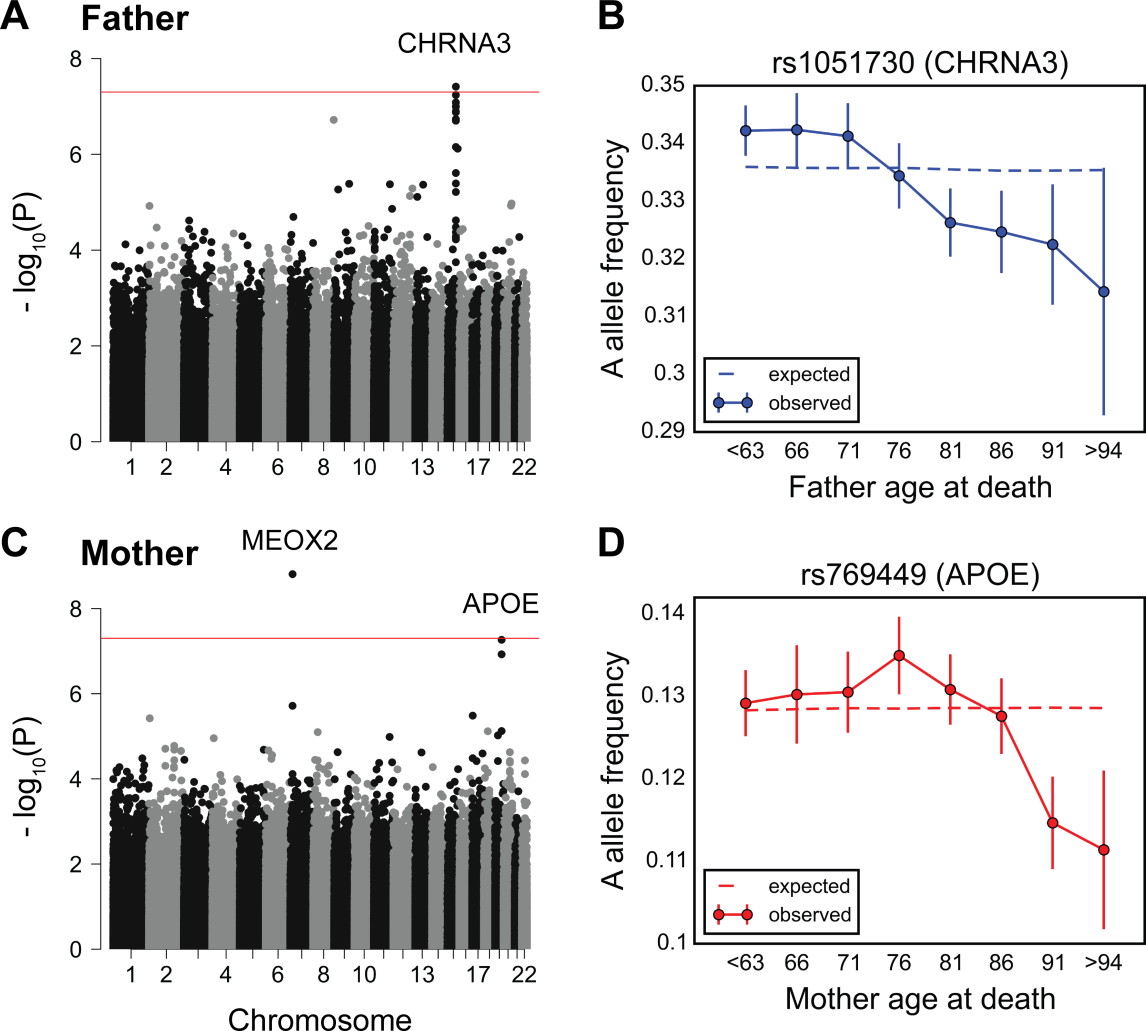
Testing for the influence of single genetic variants on age-specific mortality in the UK Biobank. (A) Manhattan plot of *P* values, obtained from testing for a change in allele frequency with age at death of fathers. (B) Allele frequency trajectory of rs1051730, within the *CHRNA3* locus, with father’s age at death. (C) Manhattan plot of *P* values, obtained from testing for a change in allele frequency with age at death of mothers. (D) Allele frequency trajectory of rs769449, within the *APOE* locus, with mother’s age at death. Red lines in (A) and (C) mark the *P* = 5 × 10^−8^ threshold. Data points in (B) and (D) are the frequencies of the risk allele within 5-year interval age bins (± 2 SE), with the center of the bin indicated on the x-axis (except for the first and the last points). The dashed line shows the expected frequency based on the null model, accounting for confounding batch effects and changes in ancestry (see Materials and methods). See S2 Data for underlying data.

Consistent with recent studies [33,34], the variants showing a genome-wide significant change in allele frequency with father’s age at death (*P* < 5 × 10^−8^) reside within a locus containing the nicotine receptor gene *CHRNA3* (Fig 3A). The A allele of the *CHRNA3* SNP rs1051730 (G, major allele; A, minor allele) has been shown to be associated with increased smoking quantity among individuals who smoke [56]. We observe a linear decrease in the frequency of the A allele of rs1051730 throughout almost all age ranges (Fig 3B) (*P*~1.3 × 10^−7^ and *P*~2.7 × 10^−10^, treating paternal age at death as a categorical or an ordinal variable, respectively). Although it does not reach genome-wide significance, this allele shows a similar trend with age in GERA (*P*~8.6 × 10^−3^, S15 Fig). We note that 30,819 of the UK Biobank individuals included in the above analysis were genotyped on the UK BiLEVE Axiom array (see Materials and methods), selected based on lung function and smoking behavior (while the remaining 57,776 samples were genotyped on the UK Biobank Axiom array) [57]. Expectedly, the frequency of the A allele is significantly higher among UK BiLEVE subjects (*P*~2.3 × 10^−10^), but the age effects are similar across both arrays (*P*~0.72, see Materials and methods).

For mother’s age at death, a SNP in a locus containing the *MEOX2* gene reached genome-wide significance (Fig 3C). The C allele of rs4721453 (T, major allele; C, minor allele) increases in frequency in the age bin centered at 76 years old (S16 Fig), i.e., there is an enrichment among individuals who died at 74 to 78 years of age, which corresponds to a deleterious effect of the C allele in this period. The trend is similar and nominally significant for other genotyped common SNPs in moderate linkage disequilibrium with rs4721453 (S16 Fig). Also, the signal for rs4721453 remains nominally significant when using subsets of individuals genotyped on the same genotyping array: 44,552 individuals on the UK Biobank Axiom array (*P*~6.6 × 10^−5^) and 25,231 individuals on the UK BiLEVE Axiom array (*P*~1.1 × 10^−4^). These observations suggest that the result is not due to genotyping errors, but it is not reproduced in GERA (*P*~0.023, S17 Fig) and so it remains to be replicated. Variants within the *APOE* locus are among the top nominally significant variants (Fig 3C). At the *APOE* SNP rs769449 (G, major allele; A, minor allele), there is an increase in the frequency of A allele at around 70 years old before subsequent decrease (Fig 3D, *P*~1.2 × 10^−7^). This pattern is consistent with our finding in GERA (of a monotonic decrease beyond 70 years of age), considering the difference in patterns expected between allele frequency trends with age among survivors versus individuals who died (S3 Fig).

We note that by considering parental age at death of the UK Biobank participants—as done also in [33–35]—we introduce a bias towards older participants, who are more likely to have deceased parents (S18 Fig). We confirmed that our top signals are not significantly affected after adjusting for age of the participants (among other potential confounders, including participants’ sex, year of birth, and socioeconomic status, as measured by the Townsend deprivation index): results remain similar for the SNP rs4721453 near *MEOX2* (*P*~2.1 × 10^−9^), *APOE* SNP rs769449 (*P*~1.5 × 10^−6^), and *CHRNA3* SNP rs1051730 (*P*~1.8 × 10^−6^ and *P*~4.3 × 10^−9^, treating paternal age at death as a categorical or an ordinal variable, respectively).

We further tested for trends in allele frequency with parental age at death that differ between fathers and mothers, focusing on 62,719 individuals with age at death information for both parents. No variant reached genome-wide significance level (S19A Fig). The rs4721453 near the *MEOX2* gene and *APOE* variant rs769449 show nominally significant sex effects (*P*~7.2 × 10^−8^ and *P*~2.2 × 10^−3^, respectively), with stronger effects in females (S19B Fig). Variants near the *CHRNA3* locus are nominally significant when using the model with parental ages at death treated as ordinal variables (rs11858836, *P*~5.7 × 10^−4^), with stronger effects in males (S19B Fig).

### Testing for changes in allele frequency at trait-associated variants

We next turned to sets of genetic variants that have been associated with polygenic traits rather than individual genetic variants. We focused on 42 polygenic traits, including disease risk and traits of evolutionary importance such as puberty timing, for which a large number of common variants have been mapped in GWASs (see S1 Table for the list of traits and number of loci) [37–40]. For each individual and each trait, we calculated a polygenic score based on the genetic variants that reached genome-wide significance level for association and then tested whether this polygenic score, or its squared difference from the mean in the case of stabilizing selection, is associated with survival (after controlling for covariates, see Materials and methods).

We first applied the Cox proportional hazards model in the UK Biobank for parental survival, focusing on the participants whose genetic ancestry is British and who reported their father’s or mother’s age or age at death (114,122 and 116,323 individuals, respectively). We then compared the results with our approach of testing for changes in the polygenic score across parental ages at death. We further analyzed 2 data sets for replication purposes: participants of the UK Biobank of non-British ancestry (29,511 and 30,372 individuals reporting father’s or mother’s age information, respectively) and the GERA cohort.

**Fig 4:**
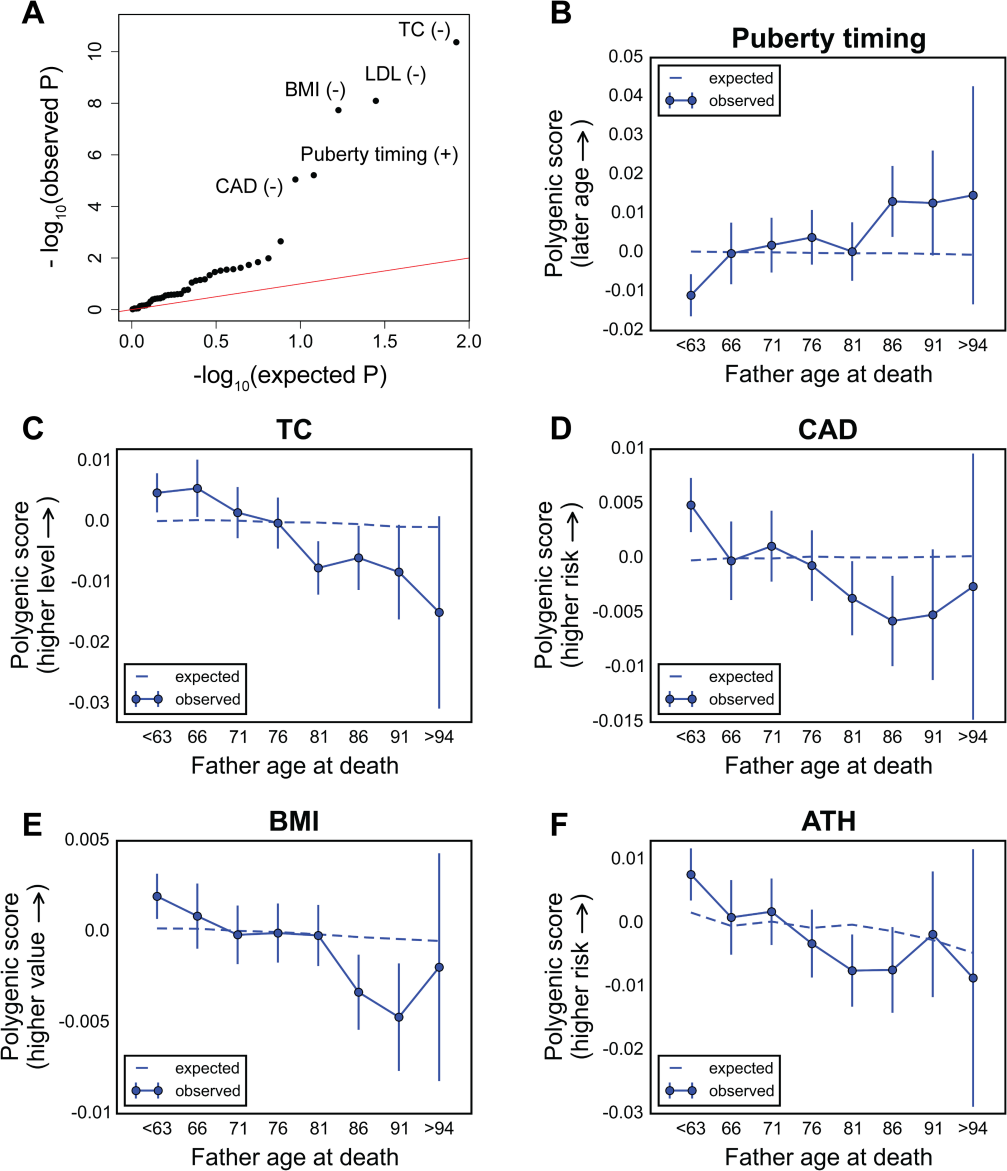
Testing for the influence of sets of trait-associated variants on survival of the fathers of UK Biobank participants. (A) Quantile-quantile plot for association between the polygenic score of 42 traits (see S1 Table) with father’s survival, using the Cox model. The red line indicates the distribution of the *P* values under the null model. Signs “+” and “−” indicate protective and detrimental effects associated with higher values of polygenic scores, respectively. See S2 Table for *P* values and hazard ratios for all traits. (B–F) Trajectory of polygenic score with age at death of fathers for top traits associated with paternal survival (only independent signals are shown, see S20 Fig): puberty timing (using age at menarche-associated variants) in males (B), total cholesterol (TC) (C), coronary artery disease (CAD) (D), body mass index (BMI) (E), and asthma (ATH) (F). Data points in (B–F) are mean polygenic scores within 5-year interval age bins (± 2 SE), with the center of the bin indicated on the x-axis (except for the first and the last points). The dashed line shows the expected score based on the null model, accounting for confounding batch effects, changes in ancestry, and participant’s age, sex, year of birth, and the Townsend index (a measure of socioeconomic status). See S2 Data for underlying data.

Using the Cox model, the scores for several traits show significant associations with father’s survival after accounting for multiple testing (Fig 4A, Table 1): total cholesterol (TC, *P*~4.3 × 10^−11^), low-density lipoproteins (LDL, *P*~8.1 × 10^−9^), body mass index (BMI, *P*~1.8 × 10^−8^), and coronary artery disease (CAD, *P*~9.0 × 10^−6^), consistent with 2 recent studies [34,35]. In addition, we uncovered significant association for the polygenic score for puberty timing (*P*~6.2 × 10^−6^); in this analysis, we used age at menarche-associated variants in females, motivated by the high genetic correlation between the timing of puberty in males and females [58]). A higher score for puberty timing is associated with longer paternal survival (per year hazard ratio of 0.96) (Table 1), indicating that variants delaying puberty timing are associated with a higher chance of survival, consistent with epidemiological studies suggesting early puberty timing to be associated with adverse health outcomes [59]. For all other traits, a higher score is negatively associated with paternal survival: 1 unit polygenic score hazard ratio of 1.09 for TC, 1.08 for LDL, 1.08 for CAD, and 1.22 for BMI (Table 1). With the exception of lipid traits, the effects on survival are not significantly changed after accounting for the effect of the polygenic score of another trait (S20 Fig). This is especially relevant to BMI and puberty timing, for which there is substantial genetic overlap [38]; the per year hazard ratio is 0.97 for the puberty timing score (*P*~4.8 × 10^−4^) after adjusting for the BMI score.

**Table 1:** Associations between sets of trait-associated variants and paternal and maternal survival among the UK Biobank participants of British ancestry, under the Cox model.

Trait	Scaling of effect	Father			Mother		
		Effect size (SE)	HR	*P* value	Effect size (SE)	HR	*P* value
Puberty timing	1 year	−0.0363 (0.0080)	0.96	6.2 × 10^−6^	−0.0278 (0.0090)	0.97	0.0020
AFB	1 year	−0.0398 (0.0180)	0.96	0.027	−0.0639 (0.0200)	0.94	0.0014
ATH	1 unit log-odds	0.0279 (0.0109)	1.03	0.010	0.0149 (0.0121)	1.02	0.22
BMI	1 SD	0.1996 (0.0355)	1.22	1.8 × 10^−8^	0.0823 (0.0395)	1.08	0.037
CAD	1 unit log-odds	0.0784 (0.0177)	1.08	9.0 × 10^−6^	0.0892 (0.0196)	1.09	5.2 × 10^−6^
HDL	1 SD	−0.0340 (0.0139)	0.97	0.014	−0.0605 (0.0154)	0.94	8.9 × 10^−5^
LDL	1 SD	0.0806 (0.0140)	1.08	8.1 × 10^−9^	0.0844 (0.0155)	1.09	5.2 × 10^−8^
TC	1 SD	0.0901 (0.0137)	1.09	4.3 × 10^−11^	0.0679 (0.0152)	1.07	7.8 × 10^−6^

**Abbreviations:** AFB, age at first birth; ATH, asthma; BMI, body mass index; CAD, coronary artery disease; HR, hazard ratio; HDL, high-density lipoproteins; LDL, low-density lipoproteins; TC, total cholesterol

Using our approach instead, i.e., considering the father’s age at death, led to very similar results. Specifically, all traits significantly associated with paternal survival show a significant change in polygenic score with father’s age at death using the model with parental ages at death treated as ordinal variables (S21 Fig): TC (*P*~8.8 × 10^−9^), CAD (*P*~3.3 × 10^−8^), puberty timing (*P*~1.6 × 10^−7^), LDL (*P*~8.6 × 10^−7^), and BMI (*P*~3.4 × 10^−6^). In addition, we uncovered significant changes in polygenic score with father’s age at death for asthma (ATH, *P*~9.4 × 10^−5^) and triglycerides (TG, *P*~4.4 × 10^−4^, the effect of which does not seem to be distinct from other lipid traits, S20 Fig). The score for puberty timing increases monotonically with the father’s age at death (Fig 4B), indicative of a protective effect of later predicted puberty timing, whereas all other traits with significant signal show a monotonic decline in score with age (Fig 4C–4F).

**Fig 5:**
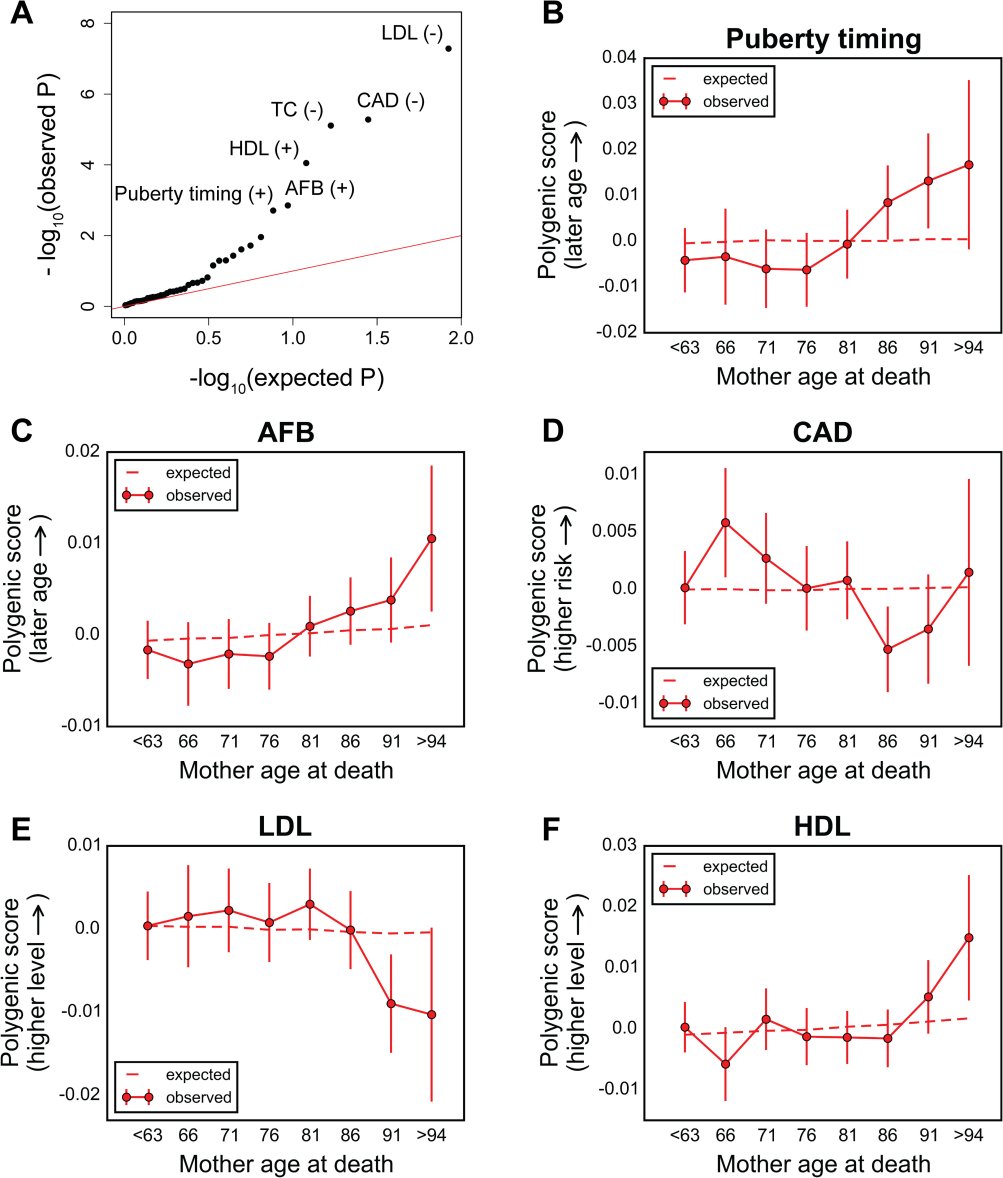
Testing for the influence of sets of trait-associated variants on survival of the mothers of UK Biobank participants. (A) Quantile-quantile plot for association between the polygenic score of 42 traits (see S1 Table) with mother’s survival, using the Cox model. The red line indicates the distribution of the *P* values under the null. Signs “+” and “−” indicate protective and detrimental effects associated with higher values of polygenic scores, respectively. See S2 Table for *P* values and hazard ratios for all traits. (B–F) Trajectory of polygenic score with age at death of mothers for top traits associated with maternal survival (only independent signals are shown, see S20 Fig): puberty timing (B), age at first birth (AFB) (C), coronary artery disease (CAD) (D), low-density lipoproteins (LDL) (E), and high-density lipoproteins (HDL) (F). Data points in (B–F) are mean polygenic scores within 5-year interval age bins (± 2 SE), with the center of the bin indicated on the x-axis (except for the first and the last points). The dashed line shows the expected score based on the null model, accounting for confounding batch effects, changes in ancestry, and participant’s age, sex, year of birth, and the Townsend index (a measure of socioeconomic status). See S2 Data for underlying data.

In a Cox survival model, for mothers as with for fathers, scores for TC, CAD, and LDL are significantly associated with survival, with similar hazard ratios (Fig 5A and Table 1): 1 unit polygenic score hazard ratio of 1.09 for LDL (*P*~5.2 × 10^−8^), 1.09 for CAD (*P*~5.2 × 10^−6^), and 1.07 for TC (*P*~7.8 × 10^−6^). In addition, the high-density lipoproteins (HDL) score is associated with maternal survival (1 standard deviation (SD) hazard ratio of 0.94, *P*~8.9 × 10^−5^). Also, suggestive evidence was detected for protective effects of increased predicted age at first birth (AFB) (per year hazard ratio of 0.94, *P*~1.4 × 10^−3^) as well as predicted puberty timing (per year hazard ratio of 0.97, *P*~2.0 × 10^−3^) (Fig 5A and Table 1). Other than the LDL and TC, all signals seem to be distinct (S20 Fig), including for puberty timing and AFB, despite the genetic correlation between the 2 phenotypes [39].

In turn, applying our approach to maternal age at death, puberty timing and AFB are the top signals (*P*~2.2 × 10^−4^ and *P*~3.1 × 10^−3^, respectively, S21 Fig). Higher polygenic scores for puberty timing are enriched among longer-lived mothers (Fig 5B), as seen for fathers. Similarly, the score for AFB increases with mother’s age at death (Fig 5C), indicating an association between variants that delay AFB and longer life span. Scores for CAD, LDL, and HDL did not show significant monotonic change across mother’s age at death bins (*P*~7.7 × 10^−3^, *P*~0.058, and *P*~0.35, respectively); however, the trends are suggestive of subtle age-dependent effects, with an effect of CAD score in middle age and late-onset effects of LDL and HDL scores (Fig 5D–5F). Testing for age by sex interactions, the TC and CAD score trends with parental ages at death are significantly different between fathers and mothers (*P*~4.0 × 10^−4^ and *P*~7.4 × 10^−4^, respectively, S22 Fig).

To further investigate the age dependency of the effects, we plotted polygenic scores among parents who had survived up to a given age as compared to the trends with parental ages at death (S23 and S24 Figs). All traits associated with paternal survival seemingly show more pronounced effects in middle age (S23 Fig). Similar patterns were observed for maternal survival-associated traits except for LDL and HDL, which had more pronounced late-age effects (S24 Fig). We also compared the hazard ratios for ages at death of ≤ 75 and > 75 years (Materials and methods), similar to a recent study [33]. Consistent with trends in scores with parental age, among the traits associated with paternal survival, almost all traits have seemingly stronger effects among younger fathers, particularly for CAD (S3 Table): 1 unit log-odds hazard ratio of 1.14 for younger fathers (*P*~2.6 × 10^−9^) and 0.99 for older fathers (*P*~0.70). Unlike in fathers, in mothers, TC, LDL, and HDL scores had more pronounced late-age effects (S3 Table): for TC, 1 SD hazard ratio of 1.03 for younger mothers (*P*~0.15) and 1.11 for older mothers (*P*~1.4 × 10^−6^) and for LDL, 1 SD hazard ratio of 1.05 for younger mothers (*P*~0.03) and 1.12 for older mothers (*P*~3.3 × 10^−8^).

Next, we sought to replicate the top associations observed among the UK Biobank participants of British ancestry (discovery cohort) in 2 other data sets: participants of the UK Biobank of non-British ancestry and the GERA cohort. Applying the Cox model using parental survival for UK Biobank participants of non-British ancestry, the direction of hazard ratios for all traits (as well as the estimated values for most traits) are consistent with the discovery cohort for both fathers and mothers (S4 Table). The congruence of results in 2 cohorts with different ancestries suggests that our top signals are not false positives caused by poor control for population structure. In the GERA cohort, we tested whether polygenic scores change with the age of the participant, similar to our approach for individual genetic variants in this cohort. All top signals except AFB have directionally consistent effects with the discovery cohort (S5 Table). Of particular interest, the strongest signal is an increase in the polygenic score for puberty timing with age of the participants (*P*~6.7 × 10^−3^, S25 Fig).

In the discovery cohort, we further investigated if there are significant changes in the squared difference of polygenic scores with parental ages at death, as might be expected if the mean value of the trait leads to the highest chance of survival. No trait shows evidence of such stabilizing selection (S26 Fig).

## Discussion

We introduced a new approach to identify genetic variants that affect survival to a given age and thus to directly observe viability selection ongoing in humans. Attractive features of the approach include that we do not need to make a decision a priori about which loci or traits matter to viability and focus not on an end point (e.g., survival to an old age) but on any shift in allele frequencies with age, thereby learning about the ages at which effects are manifest and possible differences between sexes.

To illustrate the potential of our approach, we performed a scan for genetic variants that impact age-specific mortality in the GERA and the UK Biobank cohorts. We only found a few individual genetic variants, almost all of which were identified in previous studies. This result is in some ways expected: available data only provide high power to detect effects of common variants (>0.15–0.2) on survival (Fig 1), yet if these variants were under viability selection, we would not expect them to be common, short of strong balancing selection due to trade-offs between sexes, ages, or environments. As sample sizes increase, however, the approach introduced here should provide a comprehensive picture of viability selection in humans. To illustrate this point, we repeated our power simulation with 500,000 samples and found that we should have high power to detect the trends for alleles at a couple percent frequency in the sample (S27 Fig).

Already, however, this application raises a number of interesting questions about the nature of viability selection in humans. Notably, we discovered only a few individual variants influencing viability in the 2 cohorts, most of which exert their effect late in life. On first thought, this finding may suggest such variants to be neutrally evolving. We would argue that if anything, our findings of only a few common variants with large effects on survival late in life suggest the opposite: that even variants with late-onset effects have been weeded out by purifying selection. Indeed, unless the number of loci in the genome that could give rise to such variants (i.e., the mutational target size) is tiny, other variants such as the *APOE* ε4 allele must often arise. That they are not observed when we have very high power to detect them suggests they are kept at lower frequency by purifying selection. Why might they be selected despite affecting survival only at old ages? Possible explanations include that they decrease the direct fitness of males sufficiently to be effectively selected (notably given the large, recent effective population size of humans [60]) or that they impact the inclusive fitness of males or females. If this explanation is correct, it raises the question of why the *APOE* ε4 allele has not been weeded out. We speculate that the environment has changed recently, making this allele more deleterious. For example, it has been proposed that the evolution of this allele has been influenced by changes in physical activity [61] and parasite burden [62].

Considering 42 traits that have been investigated by GWASs, we found a number of cases in which the mean polygenic score changes with age. Of course, detecting an effect of age on the traits does not imply that these are the phenotypes under viability selection, as the variants that contribute likely have pleiotropic effects on other traits [37]. Nonetheless, it is perhaps not surprising that we found detrimental effects of higher genetically predicted TC, LDL, BMI, and risk of CAD on survival, as these phenotypes are studied in GWASs precisely because of their adverse health effects. Intriguingly, however, we also found associations for fertility traits, notably, protective effects of later predicted puberty timing and AFB. If these findings reflect life-history trade-offs (e.g., longer life span at the cost of delayed reproduction), they may help to explain the persistence of extensive variation in such fitness-correlated traits [63,64]. Intriguingly, we saw a negative correlation between genetically predicted AFB and number of siblings of the UK Biobank participants, a proxy for the fertility of their parents (*P*~4.2 × 10^−8^, S28 Fig), consistent with previous reports of a genetic correlation between AFB and the number of children ever born [21,39]. These findings underscore that consideration of survival or fertility effects alone does not allow one to infer whether the net effect of a variant or set of variants is beneficial. Instead, to convert effects on viability such as those detected here or effects on fertility reported elsewhere [22,23] into an understanding of how natural selection acts on an allele requires a characterization of its effects on all components of fitness (including potentially inclusive fitness).

In this regard, it is also worth noting that while our method is designed to detect changes in allele frequencies (and in polygenic scores) caused by genetic effects on age-specific mortality, such changes could in principle also arise from effects on other components of fitness. For example, if the frequency of a genetic variant in a population decreases over decades due to an effect on fertility, its frequency would increase with the age of surviving individuals sampled at a given time (as in the GERA cohort). This confounding is less of an issue when considering effects on the age at death (what we measured in the UK Biobank). Nonetheless, even in the UK Biobank, fertility effects may manifest as effects on age at death; for example, when sampling a cohort of children, parents with later ages at death are possibly born earlier (S29 Fig). To this end, in the UK Biobank, we accounted for changes in allele frequencies with year of birth of the participants themselves (ideally, we would want to condition on parents born at similar times, which we cannot do; instead, we used year of birth of the participants as an estimator for year of birth of the parents). Thus, we believe our results in the UK Biobank not to be confounded by fertility effects. Moreover, a number of our findings in this study are consistent with prior knowledge of effects on survival, such as those for disease risk variants like the *APOE* ε4 allele. Nonetheless, some caution is required in interpreting trends with age as strictly reflecting viability effects.

Also of interest are the marked differences between males and females in our analysis of mothers and fathers of individuals in the UK Biobank. The differences between sexes are most notable at the *CHRNA3* locus, which shows a strong effect only in fathers, and sets of genetic variants associated with risk of CAD and cholesterol levels, which exhibit different age-dependent effects between fathers and mothers. Results for the *CHRNA3* locus, in which variants are associated with the amount of smoking among smokers, may reflect a gene-by-environment interaction rather than a sex effect per se. Consistent with a more pronounced effect on male than female age at death, smoking prevalence in men has been consistently higher than women over the past few decades in the United Kingdom: from 1970 to 2000, smoking prevalence decreased from around 70% to 36% in middle-aged men, compared to from around 50% to 28% in middle-aged women [65].

Moving forward, the application of approaches such as ours to the millions of samples in the pipeline (such as the UK Biobank [66], the Precision Medicine Initiative program [67], and the BioVU biobank at Vanderbilt University [68]) will allow viability effects of rare as well as common alleles to be examined. These analyses will provide a comprehensive answer to the question of which loci affect survival, helping to address long-standing open questions such as the relative importance of viability selection in shaping genetic variation and the extent to which genetic variation is maintained by fitness trade-offs between sexes or across ages.

## Materials and methods

### Ethics statement

This study used data sets from the UK Biobank (application number 11138), as approved by the UK Biobank Board, and the Genetic Epidemiology Research on Adult Health and Aging (GERA), obtained through dbGaP (request numbers 28113–4 and 57119–2) and approved by Columbia University Institutional Review Board, protocols AAAQ2700 and AAAN4411.

### Data sets

#### GERA cohort

We performed our analyses on the data for 62,318 participants of the GERA cohort (who are members of the Kaiser Permanente Medical Care Plan, Northern California Region and participating in its Research Program on Genes, Environment, and Health), self-reported to be “White-European American”, “South Asian”, “Middle-Eastern”, or “Ashkenazi” but no other ethnicities among a list of 23 choices on the GERA survey, and genotyped on a custom array at 670,176 SNPs designed for Non-Hispanic white individuals [31,32]. We considered the age of the participants and the number of years they were enrolled in the Kaiser Permanente Medical Care Plan at the time of the survey (year 2007).

#### UK Biobank

We performed our analyses on the data for 152,729 participants of the UK Biobank study, focusing on 120,286 individuals identified to be “British” by genetic analysis, and all other individuals for replication. They were genotyped on the UK Biobank Axiom or the UK BiLEVE Axiom SNP arrays at a total of 847,441 SNPs in the interim release [57,66].

### QC

#### GERA cohort

We used PLINK v1.9 [69] to remove individuals with missing sex information or with a mismatch between genotype data and sex information, individuals with <96% call rate, and individuals with at least one parent in the sample. We validated self-reported European ancestries using PCA, see below, and removed individuals identified as non-European (S4 and S5 Figs). In the end, 57,696 individuals remained.

Using PLINK, we removed SNPs with <1% minor allele frequency, SNPs with <95% call rate, and SNPs failing a Hardy-Weinberg equilibrium test with *P* < 10^−8^ (filtering based on HWE test could potentially exclude true signals of viability selection if selection coefficients were very large [70], but this possibility is much less likely than genotyping error). We additionally tested for a correlation between age (or sex) and proportion of missing data, which can induce artificial change in the allele frequencies as a function of age (or sex). We thus removed SNPs showing a significant age-missingness or sex-missingness correlation, defined as a chi-squared test with *P* < 10^−7^. After these steps, 583,357 SNPs remained.

We imputed the genotypes of the filtered GERA individuals using post-QC SNPs and using the 1000 Genomes phase 3 haplotypes as a reference panel [71]. We phased observed genotypes using EAGLE v1.0 software [72]. The inferred haplotypes were then passed to IMPUTE2 v2.3.2 software for imputation in chunks of 1 Mb using the default parameters of the software [73]. To gain computational speed, variants with minor allele frequency of <0.005 in the 1000 Genomes European populations were removed from the reference panel. This step should not affect our analysis because our statistical model is not well powered for rare variants, given the GERA data sample size. We called imputed genotypes with posterior probability of >0.9 and then filtered the imputed genotypes, removing variants with IMPUTE2 info score of <0.5 and with minor allele frequency of <0.01. We also used imputation with a leave-one-out approach [74] to impose a second stage of QC on genotyped SNPs, removing SNPs that were imputed back with high reported certainty (info score >0.5) and with <90% concordance between the imputed and the original genotypes. These yielded a total of 8,868,517 imputed and genotyped biallelic SNPs and indels.

For our analysis of the *APOE* alleles (ε2, ε3, and ε4), which are defined by rs7412 and rs429358 SNPs [55], given the lack of tag SNPs for all 3 alleles, we kept a subset of 38,703 individuals with no poorly imputed genotypes for these 2 SNPs, for whom the count of each *APOE* allele could be determined unambiguously.

#### UK Biobank

In the UK Biobank, we obtained sets of genotype calls and the output of imputation as performed by the UK Biobank researchers [57,75]. We first applied QC metrics to the autosomal genotyped SNPs, focusing on the individuals of British genetic ancestry. We used PLINK to remove SNPs with minor allele frequency of <0.01, SNPs with <95% call rate, and SNPs failing a Hardy-Weinberg equilibrium test with *P* < 10^−8^. These filters were applied separately to individuals genotyped on the UK Biobank Axiom and the UK BiLEVE Axiom arrays. Then, we divided the genotyped SNPs into 3 sets (SNPs specific to either array and shared SNPs) and then performed additional QC on each set separately: we removed SNPs with significant allele frequency difference between genotyped and imputed calls (chi-squared test *P* < 10^−5^) and SNPs showing a significant correlation between proportion of missing data and age or sex of the participants as well as with participants’ father’s or mother’s age at death (chi-squared test *P* < 10^−7^). We then extracted this list of SNPs from the imputed genotype files available from the UK Biobank (we did not use the full set of imputed genotypes). From this set, we removed SNPs with minor allele frequency of <0.01, SNPs with <95% call rate, and SNPs failing a Hardy-Weinberg equilibrium test with *P* < 10^−8^, yielding 590,437 SNPs. For variants influencing quantitative traits, we first extracted them from imputed genotype files and then imposed similar QC measures as above. For individuals of non-British ancestry, we first extracted the trait-influencing variants from imputed genotype files and then removed SNPs with minor allele frequency of <0.01 and SNPs with <90% call rate.

Each participant was asked to provide the survival status and age of their father and their mother on each assessment visit. For each participant who reported an age at death of father and/or mother, we averaged over the ages reported at recruitment and any subsequent repeat assessment visits and used PLINK to exclude individuals with >5-year variation in their answers across visits (around 800 individuals). For those reporting their parents to be alive, the latest assessment visit was considered. We also removed adopted individuals, individuals with a mismatch between genotype data and sex information, and individuals with missing values for the covariates, resulting in 88,595 individuals of British ancestry with age at death information for their father, 71,783 individuals of British ancestry with age at death information for their mother, and 62,719 individuals of British ancestry with age at death information for both parents. For the survival analyses, we further removed individuals with evidently invalid parental survival status, particularly parental ages at death values smaller than their age when still alive, resulting in 114,122 and 116,323 individuals of British ancestry with paternal and maternal survival information, respectively. With similar QC measures, 29,511 and 30,372 individuals of non-British ancestry with paternal and maternal survival information, respectively, were analyzed.

### PCA

We performed PCA using the EIGENSOFT v6.0.1 package with the fastpca algorithm [76,77] for 2 purposes: (i) as a QC on individuals to validate self-reported European ancestries (only in GERA data set) and (ii) to correct for population structure in our statistical model (for individuals in the UK Biobank of non-British ancestry, we used the PCs provided with the data).

#### European ancestry validation

We used more stringent QC criteria specifically for the PCA compared to the QC steps described above. We filtered a subset of 157,277 SNPs in GERA, retaining SNPs shared between the data sets and the 1000 Genomes phase 3 data, removing nonautosomal SNPs, SNPs with minor allele frequency of <0.01, SNPs with <99% call rate, and SNPs failing a Hardy-Weinberg equilibrium test with *P* < 10^−6^. We then performed LD pruning using PLINK with pairwise *r*^2^ < 0.2 in windows of 50 SNPs shifting every 10 SNPs. We used these SNPs to infer principal components for the 1000 Genomes phase 3 data [71]. We then projected individuals onto these PCs. We observed that the majority of individuals have European ancestry and marked individuals with PCs deviating from the population mean for any of the first 6 PCs as non-European (S4 and S5 Figs).

#### Control for population structure

After the main QC stage, additional QC steps (as in European ancestry validation) were implemented for PCA. In the UK Biobank, we also removed inversion variants on chromosome 8, which otherwise dominate the PC2 (not shown). A subset of 156,721 SNPs in GERA and 207,657 SNPs in the UK Biobank was then used to infer PCs for individuals passing QC (S1 Fig). The first 10 PCs were used as covariates in our statistical model.

### Quantitative traits

We downloaded the list of variants contributing to 39 traits (all traits but age at menarche, AFB, and age at natural menopause) and their effect sizes recently described in Pickrell et al. [37] from: https://github.com/PickrellLab/gwas-pw-paper/tree/master/all_single. For age at menarche, we used the variants and effect sizes recently identified by Day et al. [38]. We used variants associated with AFB from Barban et al. identified in either sex-specific analyses or analyses of both sexes and used the effect sizes estimated in the combined analysis [39]. We used age at natural menopause-associated variants and their effect sizes from Day et al. [40]. For all traits, we used variants that were genotyped/imputed with high quality in our data (see S1 Table).

### Statistical model

#### An individual variant

Using a logistic regression, we predicted the genotype of individual *j* (the counts of an arbitrarily selected reference allele, *G*_*ij*_ = 0,1, or 2) at variant *i*, using the individual’s ancestry, the batch in which the individual was genotyped, and the individual’s age (as well as sex, see below) as explanatory variables. Specifically, the distribution of *G*_*ij*_ is *Bin*(2,*p*_*ij*_), where *p*_*ij*_, the probability of observing the reference allele for individual *j* at variant *i*, is related to explanatory variables as:

$$\log(\frac{p_{ij}}{1-p_{ij}})=\alpha +\sum_{l=1}^{10}\beta_{l}PC_{lj}+\sum_{m}\gamma_{m}I_{j\in {BATCH}_{m}}+\sum_{n}\kappa_{n}J_{j\in BIN_{n}}$$

where *β*_*l*_ is the effect of principal component *l* (to account for population structure), *γ*_*m*_ is the effect of being in batch *m* (to account for potential systematic differences between genotyping packages), *κ*_*n*_ is the effect of being in age bin *n*, obtained by regression across individuals with nonmissing genotypes at variant *i*, and *I* and *J* are indicator variables for the genotyping batch and age bin, respectively. In the version of the model in which we treated age as an ordinal variable, we replaced *J* age bin variables with 1 age variable. In the GERA data set, age binning is over the age of the participants in 14 categories, from age 19 onwards, in 5-year intervals. For replication purposes, we further binned the ages in 7 categories, in 10-year intervals, to boost our power by increasing the sample size per bin, particularly for younger age bins. In the UK Biobank, we binned the age at death of father or mother over 8 categories, from age 63 onwards, in 5-year intervals. In the UK Biobank, we included all ages at death below 63 in one age bin to minimize the potential noise caused by accidental deaths at young ages.

We tested for an effect of age categories by a likelihood ratio test with a null model using only the covariates (PCs and batch terms) (*H*_0_: *κ*_*n*_ = 0, for all *n*) and an alternative also including age terms as predictors (*H*_1_: *κ*_*n*_ ≠ 0, for at least one *n*):

$$\left\{ \begin{array}{l} H_{0}:\;\log(\frac{p_{ij}}{1-p_{ij}})=\alpha +\sum_{l=1}^{10}\beta_{l}PC_{lj}+\sum_{m}\gamma_{m}I_{j\in {BATCH}_{m}}\\ H_{1}:\;\log(\frac{p_{ij}}{1-p_{ij}})=\alpha +\sum_{l=1}^{10}\beta_{l}PC_{lj}+\sum_{m}\gamma_{m}I_{j\in {BATCH}_{m}}+\sum_{n}\kappa_{n}J_{j\in BIN_{n}} \end{array}\right.$$

To test for age by sex effects in GERA, we included 2 sets of additional predictors. The first consists in an indicator variable for sex, *K*, which is included to capture possible sex effects induced by potential genotyping errors or mismapping of sex chromosome–linked alleles (we note that because of Hardy-Weinberg equilibrium, mean allele frequency difference between males and females is not expected). The second set of predictors consists in age by sex terms, *J* × *K*. We then compared a model with age and sex terms as predictors to a model also including age by sex terms. To test for sex effects in the UK Biobank, we compared a model with both father and mother age terms separately as predictors to a model with 1 set of age categories for average age at death of both parents, only for individuals reporting the age at death for both parents. In all models, PCs and batch terms were incorporated as covariates. For the top SNPs in the UK Biobank, we additionally tested models also including as covariates the participants’ age, sex, year of birth, and the Townsend index (a measure of socioeconomic status). For rs1051730, we also tested whether allele frequencies or trends in allele frequencies with the father’s age at death vary significantly across the UK Biobank genotyping arrays after adjusting for population structures, using similar models as described above.

#### Set of variants

As for the model described above for an individual variant, we investigated age and age by sex effects on quantitative traits for which a large number of common genetic variants have been identified in GWASs. For a given trait, we used a linear regression with the same covariates and predictors as for the model for an individual variant to predict the polygenic score for individual *j*, *S*_*j*_ (estimated by summing the previously estimated effects of single variants, assuming additivity and that the effect sizes are similar in the GWAS panels and the cohorts considered here):

$$S_{j}=\alpha +\sum_{l=1}^{10}\beta_{l}PC_{lj}+\sum_{m}\gamma_{m}I_{j\in {BATCH}_{m}}+\sum_{n}\kappa_{n}J_{j\in BIN_{n}}+\varepsilon_{j}$$

*S*_*j*_ is calculated as ∑*a*_*i*_*G*_*ij*_ + ∑2*a*_*i*_*q*_*i*_ (standardized to mean 0), where the first sum is across variants with nonmissing genotypes, *a*_*i*_ is the effect size for the arbitrary selected reference allele at variant *i*, and the second sum is across the variants with missing genotypes estimating their contribution assuming Hardy-Weinberg equilibrium where *q*_*i*_ is the frequency of the alternate allele. Likelihood ratio tests, as described above, were used to test for age and age by sex effects. In the UK Biobank, we additionally adjusted for participants’ age, sex, year of birth, and the Townsend index.

To evaluate the possibility of stabilizing selection on a trait, we applied the same model, but instead of the polygenic score, we regressed the squared difference of the score from the mean in each bin, ${\left(S_{j}-{\bar{S}}_{BIN,j}\right)}^{2}$, on the predictors, where ${\bar{S}}_{BIN,j}$ is the mean score in the age bin to which individual *j* belongs.

We also used the Cox proportional hazards model [46] to evaluate the association between polygenic scores and parental survival in the UK Biobank. Compared to the model described above, this approach presents the advantage of allowing data from participants with alive parents to be incorporated but has the disadvantage of assuming fixed effects across all ages. Under this model, at a given time *t* (age in our application):

$$\log\lambda_{j}(t)=\log\lambda_{0}(t)+\sum_{l=1}^{10}\beta_{l}PC_{lj}+\sum_{m}\gamma_{m}I_{j\in {BATCH}_{m}}+\kappa \;S_{j}$$

where *λ* is the hazard rate (probability of death within *t* + *dt* conditional on survival to time *t*) given the covariates, and *λ*_0_ is the baseline hazard rate that describes the risk for individuals with the value of 0 for all predictors. Not shown in the equation above are covariates to adjust for participants’ age, sex, year of birth, and the Townsend index. Using the R package “Survival” [78], for a given trait, we tested for a significant effect of polygenic score (*κ* ≠ 0). In addition, to assess the interdependence of detected effects (S20 Fig), for each pair of traits [*a*, *b*], we tested for the effect of the polygenic score for trait *a* but also incorporated the polygenic score for trait *b* as a covariate in the null model (in addition to the covariates mentioned above).

We further investigated the age dependency of the effects in the framework of the survival analysis by comparing hazard ratios in 2 age categories: ages at death of ≤ 75 and > 75 years. For the category of ages at death ≤ 75 years, all parental ages were included in the analysis, and parents with ages at death beyond 75 years were marked as alive. For the category of ages at death > 75 years, only parents who survived beyond 75 years were considered.

All Manhattan and quantile-quantile plots were generated using qqman [79] and GWASTools [80] packages.

### Power simulations

We ran simulations to determine the power of our statistical model to detect deviation of allele frequency trends with age across 14 age categories mimicking the GERA cohort’s age structure (57,696 individuals with age distribution as in S2 Fig) from a null model, which for simplicity was no change in frequency with age, i.e., no changes as a result of age-dependent variation in population structure and batch effects. For a given trend in frequency of an allele with age, we generated 1,000 simulated trends in which the distribution of the number of the alleles in age bin *i* is *Bin*(2*N*_*i*_, *f*_*i*_), where *N*_*i*_ and *f*_*i*_ are the sample size and the sample allele frequency in bin *i*. We then estimated the power to detect the trend as the fraction of cases in which *P* < 5 × 10^−8^, by a chi-squared test.

### Survival simulations

We ran simulations to investigate the relationship between allele frequency with age of the surviving individuals and the age of the individuals who died in a cohort. We simulated 2 × 10^6^ individuals going forward in time in 1-year increments. For each time step forward, we tuned the chance of survival of the individuals based on their count of a risk allele for a given variant such that the number of individuals dying in the increment complies with: (i) a normal distribution of ages at death with mean of 70 years and standard deviation of 13 years, roughly as is observed for parental ages at death in the UK Biobank and (ii) a given frequency of the risk allele among those who survive. Specifically, we modeled the survival rate of the population, *S*, as the weighted mean for 2-alleles carriers, *S*_2_, 1-allele carriers, *S*_1_, and noncarriers, *S*_0_:

$$S(x)=\sum_{i=0}^{2}f_{i}\;S_{i}(x)$$

where *f* denotes the frequency of genotypes in the population and *x* denotes the age. *S*_*i*_ and *S* are related: *S*_*i*_(*x*) = *S*(*x*) *f*_*i*_(*x*)/*f*_*i*_, where *f*_*i*_(*x*) is the genotype frequency among individuals survived up to age *x*. Given a trend in allele frequency with age, we calculated genotype frequencies with age assuming Hardy-Weinberg equilibrium and then estimated genotype-dependent chance of survival, *S*_*i*_(*x*), taking *S*(*x*) as the survival function for *N*(70, 13^2^).

## Supporting information

S1 Fig Results of principal component analysis (PCA). (A) PCA on 57,696 GERA individuals after quality control removing “non-European” individuals. (B) PCA on 120,286 UK Biobank participants of British ancestry. Results are in agreement with recent studies of these data [77,81].
(TIF)

S2 Fig Age distribution of the GERA individuals at the time of the survey, year 2007. The labels on the x-axis indicate the center of 5-year interval age bins (except the last category). See S1 Data for underlying data.
(TIF)

S3 Fig Comparison of trends in allele frequency with age and age at death. (A) Simulated allele frequencies among surviving individuals, reproducing trends as in Fig 1A. (B) Trends in allele frequency among individuals who died, corresponding to the trends in (A). Points are allele frequencies within 5-year interval age bins (mean ± 2 SE).
(TIF)

S4 Fig Validation of European ancestry in GERA. Shown are PCs inferred for all 26 populations in the 1000 Genomes Project phase 3 data. For clarity, in each plot, only a few representative populations are shown. GERA individuals (blue dots) are projected on the inferred PCs. The dashed lines correspond to the dashed lines in S5 Fig, delimiting the majority of GERA individuals.
(TIF)

S5 Fig Distribution of GERA individuals for PCs inferred from 1000 Genome Project phase 3 data. The dashed lines enclose the majority of the data points; beyond, individuals were labeled as “non-Europeans.”
(TIF)

S6 Fig Quantile-quantile plots for model results for individual variants in GERA. Quantile-quantile plots for age (A) and age by sex (B) effects. The red lines indicate the distribution of the *P* values under the null model (of no age or age by sex effect) and the shaded bands represent the 95% confidence intervals, assuming independent SNPs. See S1 Data for underlying data.
(TIF)

S7 Fig Frequency of the G allele of rs4988235 with age of the GERA participants. The data points are the frequencies within 5-year interval age bins (± 2 SE). The x-axis indicates the center of the age bin (except for the first and the last bins). Bins with ages below 38 years are merged into 1 bin because of the relatively small sample sizes per bin. The dashed line shows the expected frequency based on the null model, accounting for confounding batch effects and, importantly, changes in ancestry. See S1 Data for underlying data.
(TIF)

S8 Fig Regional plot for the *APOE* locus. The y-axis shows *P* values obtained from a test of the influence of single genetic variants on age-specific mortality in GERA.
(TIF)

S9 Fig Frequency of rs6857 genotypes with age in GERA. Frequency of noncarriers (A), heterozygous (B), and homozygous (C) carriers of the risk allele for rs6857, tagging the ε4 allele of the *APOE* gene, across GERA age bins. Data points are frequencies within 5-year interval age bins (± 2 SE), with the center of the bin indicated on the x-axis (except for the first and the last bins). Bins with ages below 38 years are merged into 1 bin because of the relatively small sample sizes per bin. The dashed line shows the expected frequency based on the null model, accounting for confounding batch effects and changes in ancestry. See S1 Data for underlying data.
(TIF)

S10 Fig Frequency of rs6857 genotypes with age among males and females in GERA. Frequency of noncarriers (A), heterozygous (B), and homozygous (C) carriers of the risk allele for rs6857, tagging the ε4 allele of the *APOE* gene, across GERA age bins. Data points are frequencies within 5-year interval age bins (± 2 SE), with the center of the bin indicated on the x-axis (except for the first and the last bins). Bins with ages below 38 years are merged into 1 bin because of the relatively small sample sizes per bin. The dashed line shows the expected frequency based on the null model, accounting for confounding batch effects and changes in ancestry. See S1 Data for underlying data.
(TIF)

S11 Fig Frequency of the *APOE* gene alleles with age in GERA. Frequency of the ε2 (A), ε3 (B), and ε4 (C) alleles across GERA age bins. Data points are frequencies within 5-year interval age bins (± 2 SE), with the center of the bin indicated on the x-axis (except for the first and the last bins). Bins with ages below 38 years are merged into 1 bin because of the relatively small sample sizes per bin. The dashed line shows the expected frequency based on the null model, accounting for confounding batch effects and changes in ancestry. See S1 Data for underlying data.
(TIF)

S12 Fig Enrollments of individuals in the Kaiser Permanente Medical Care Plan. (A) Years enrolled in the care plan at the time of the survey (mean ± SD) per age bin. The x-axis indicates the center of 5-year interval age bins (except the last category). (B) Years enrolled in the plan (mean ± SD) for individuals >70 years old versus the rs6857 (*APOE*) genotype that they carry. See S1 Data for underlying data.
(TIF)

S13 Fig Testing for the influence of single genetic variants on age-specific mortality in the GERA cohort. Manhattan plot of *P* values testing for a change in allele frequency with age using the version of the model with age treated as an ordinal variable. The plot only includes the filtered genotyped SNPs in the GERA study. Red line marks the *P* = 5 × 10^−8^ threshold. The signal for variant on chromosome 18 is presumably caused by genotyping error, as other closely linked variants did not show a similar behavior, and the signal was lost when the variant was imputed using a leave-one-out approach. See S1 Data for underlying data.
(TIF)

S14 Fig Quantile-quantile plots for model results for individual variants in the UK Biobank. Quantile-quantile plots for significant change in allele frequency with father’s (A) and mother’s (B) age at death. The red lines indicate distribution of the *P* values under the null (no change in frequency) and the shaded bands represent the 95% confidence intervals, assuming independent SNPs. See S2 Data for underlying data.
(TIF)

S15 Fig Effect of rs1051730 (*CHRNA3*) on survival in GERA (*P*~8.6 × 10^−3^). Allele frequency trajectory of rs1051730 with age for males and females together (A) and separately (B). The data points are the frequencies within 10-year interval age bins (± 2 SE). The x-axis indicates the center of the age bin (except for the first and the last bins). The dashed line shows the expected frequency based on the null model, accounting for confounding batch effects and changes in ancestry. See S1 Data for underlying data.
(TIF)

S16 Fig Allele frequencies of variants in the *MEOX2* locus with mother’s age at death in the UK Biobank. Plots are for 4 genotyped SNPs in moderate linkage disequilibrium with *P* < 10^−4^ for the change in allele frequency with mother’s age at death. Data points are frequencies within 5-year interval age bins (± 2 SE), with the center of the bin indicated on the x-axis (except for the first and the last bins). The dashed line shows the expected frequency based on the null model, accounting for confounding batch effects and changes in ancestry. See S2 Data for underlying data.
(TIF)

S17 Fig No significant effect of rs4721453 (near *MEOX2*) on survival in GERA (*P*~0.023). Allele frequency trajectory of rs4721453 with age for males and females together (A) and separately (B). The data points are the frequencies within 10-year interval age bins (± 2 SE). The x-axis indicates the center of the age bin (except for the first and the last bins). The dashed line shows the expected frequency based on the null model, accounting for confounding batch effects and changes in ancestry. See S1 Data for underlying data.
(TIF)

S18 Fig Ascertainment bias towards older participants introduced by using parental ages at death in the UK Biobank. Fraction of the participants in each age bin (bin size of 3 years) who reported their father’s or mother’s age at death. See S2 Data for underlying data.
(TIF)

S19 Fig Testing for a significant age by sex effect of individual genetic variants in the UK Biobank. (A) Manhattan plot of *P* values, testing a difference between fathers and mothers in the change in allele frequency with parental age at death. (B) Allele frequencies as a function of father’s and mother’s age at death for top SNPs with age effects: rs4721453 (near *MEOX2*), rs11858836 (near *CHRNA3*), and rs769449 (*APOE*). The data points are the frequencies within 5-year interval age bins (± 2 SE). The x-axis indicates the center of the age bin (except for the first and the last bins). The dashed line shows the expected frequency based on the null model, accounting for confounding batch effects and changes in ancestry. See S2 Data for underlying data.
(TIF)

S20 Fig Heat map showing interdependence between the age effects of pairs of trait-associated variants in the UK Biobank. Each square [*i*,*j*] shows the effect size (log[hazard ratio]) of the polygenic score for trait *i* on father’s (left) or mother’s (right) survival in the Cox model, after accounting for the effect of the polygenic score of trait *j* (i.e., incorporating the polygenic score for trait *j* as a covariate in the null model, see Materials and methods). Squares on the diagonal (marked by black rectangles) show the effect size of the polygenic score without accounting for the score for other traits. See S2 Data for underlying data.
(TIF)

S21 Fig Testing for age effect of sets of trait-associated variants in the UK Biobank, treating age variables as ordinal. Quantile-quantile plots for changes in polygenic score of 42 traits (see S1 Table) with father’s (A) or mother’s (B) age at death, after accounting for confounding batch effects, changes in ancestry, and the participant’s age, sex, year of birth, and the Townsend index (a measure of socioeconomic status). The red lines indicate the distribution of the *P* values under the null model. Signs “+” and “−” indicate protective and deleterious effects associated with higher values of polygenic scores, respectively. See S2 Data for underlying data.
(TIF)

S22 Fig Testing for age by sex effect of sets of trait-associated variants in the UK Biobank. (A) Quantile-quantile plot for changes in polygenic score of 42 traits (see S1 Table) with parental ages at death that are different between fathers and mothers of the UK Biobank participants. The red lines indicate the distribution of the *P* values under the null. (B) The trend in polygenic score with parental ages at death for total cholesterol and coronary artery disease, which show significant age by sex effects. The data points are the mean polygenic scores within 5-year interval age bins (± 2 SE). The x-axis indicates the center of the age bin (except for the first and the last bins). The dashed line shows the expected polygenic score based on the null model, accounting for confounding batch effects, changes in ancestry, and the participant’s age, sex, year of birth, and the Townsend index (a measure of socioeconomic status). See S2 Data for underlying data.
(TIF)

S23 Fig Trajectories of polygenic scores with father’s age for traits associated with paternal survival in the UK Biobank. Each plot shows (in blue) the mean polygenic score (± 2 SE) among the fathers who died in a 5-year interval centered around the plotted discs, and (in black) the mean polygenic score among fathers alive up to a given age, i.e., all fathers with age or age at death (if deceased) exceeding a given age. The dashed lines show the expected changes in polygenic scores based on the null model. If there is no effect of the score on survival at a given time (age), then the score among those who died (blue disc) should be the same as the score among those who were alive at the previous time interval. Thus, the divergence between the blue and the black lines in any time interval is an indicator of the effect of the score on survival (and its direction) within that interval. The precise effect, however, also depends on the total hazard rate of the sample, which varies by age. See S2 Data for underlying data.
(TIF)

S24 Fig Trajectories of polygenic scores with mother’s age for traits associated with maternal survival in the UK Biobank. Same as S23 Fig, but plotted for mothers (with red instead of blue). See S2 Data for underlying data.
(TIF)

S25 Fig Protective effect of later predicted puberty timing on survival in GERA (*P*~6.7 × 10^−3^). Polygenic score for puberty timing with age of the participants. The data points are the mean scores within 10-year interval age bins (± 2 SE). The x-axis indicates the center of the age bin (except for the first and the last bins). The dashed line shows the expected score based on the null model, accounting for confounding batch effects and changes in ancestry. See S1 Data for underlying data.
(TIF)

S26 Fig Testing for stabilizing selection on traits in the UK Biobank. Quantile-quantile plots testing for a change in the squared difference of polygenic score from the mean with fathers’ (A) and mothers’ (B) age at death, treating age variables as ordinal. 42 traits were tested (see S1 Table). The red line indicates the distribution of the *P* values under the null model. See S2 Data for underlying data.
(TIF)

S27 Fig Power of the model to detect changes in allele frequency with age. Same as Fig 1, but with 500,000 samples evenly distributed among age categories and only showing the results using models with age treated as a categorical variable. As can be seen, there should be substantial power to detect such effects even for relatively rare variants (i.e., at a couple of percent frequency in the population).
(TIF)

S28 Fig Association between variants influencing age at first birth and apparent fertility in the UK Biobank (*P*~4.2 × 10^−8^). Polygenic score versus the number of siblings for 112,130 participants with mother’s age ≥ 50 years. Data points are mean scores (± 2 SE). The polygenic score was regressed on the number of siblings, accounting for the confounding batch effects, changes in ancestry, and the participant’s age, sex, year of birth, and the Townsend index (a measure of socioeconomic status). The dashed line shows the expected score based on the null model. See S2 Data for underlying data.
(TIF)

S29 Fig Ascertainment bias towards older participants for older parental age at death categories in the UK Biobank. Fraction of the participants > 61 years old (last 3 age categories in S18 Fig) in each parental age bin. Assuming parents of older participants on average belong to earlier generations, older age at death categories will contain parents born earlier. See S2 Data for underlying data.
(TIF)

S1 Table List of phenotypes and abbreviations. The numbers of loci passing quality control measures are shown for each data set.
(DOCX)

S2 Table Results of the Cox model for association of polygenic scores for 42 traits with survival of parents of the UK Biobank participants. (XLSX)

S3 Table Age dependency of hazard ratios for the top associations with parental survival in the UK Biobank under the Cox model. (DOCX)

S4 Table Replication of associations in the discovery panel (UK Biobank individuals of British ancestry) in the UK Biobank participants of non-British ancestry. (DOCX)

S5 Table Testing for change in polygenic scores with age of the GERA participants. (DOCX)

S1 Data Numerical values underlying the main and supplemental figures presenting analyses on the GERA data set. For genome-wide analyses, data for SNPs with *P* < 10^−5^ are shown.
(XLSX)

S2 Data Numerical values underlying the main and supplemental figures presenting analyses on the UK Biobank data set. For genome-wide analyses, data for SNPs with *P* < 10^−5^ are shown.
(XLSX)

## Abbreviations

AD: Alzheimer disease
AFB: age at first birth
ATH: asthma
BMI: body mass index
CAD: coronary artery disease
GERA: Genetic Epidemiology Research on Adult Health and Aging
GWAS: genome-wide association study
HDL: high-density lipoproteins
LDL: low-density lipoproteins
PCA: principal component analysis
QC: quality control
SNP: single-nucleotide polymorphism
TC: total cholesterol
TG: triglycerides
