# Supplementary figures and images for: Identifying genetic variants that affect viability in large cohorts
Source: PLoS Biol. 2017 Sep 5;15(9):e2002458. doi: 10.1371/journal.pbio.2002458 (PMC5584811; doi:10.1371/journal.pbio.2002458)

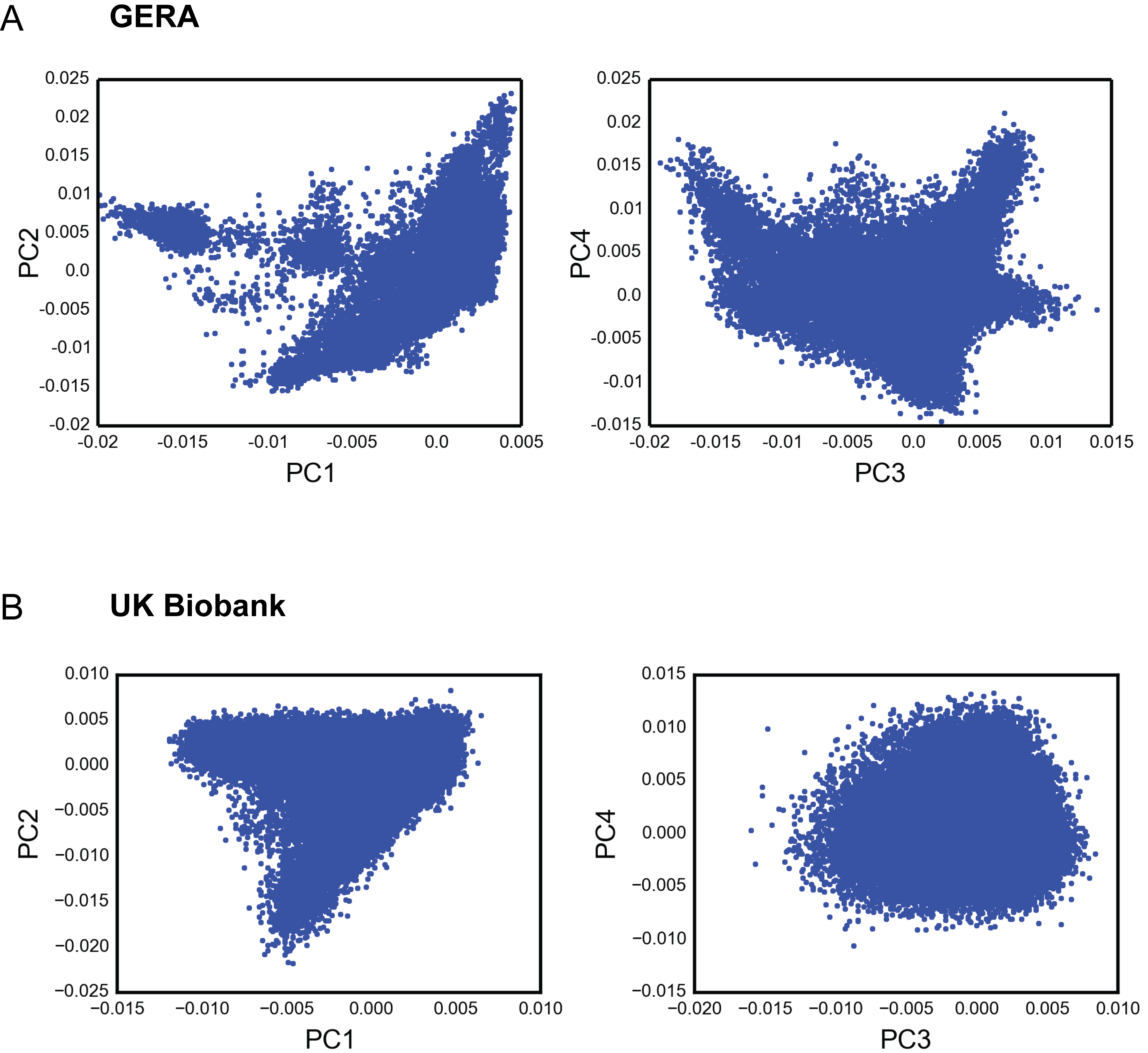

Supplement: S1 Fig — (A) PCA on 57,696 GERA individuals after quality control removing “non-European” individuals. (B) PCA on 120,286 UK Biobank participants of British ancestry. Results are in agreement with recent studies of these data [77,81]. (TIF) [file pbio.2002458.s001.tif]

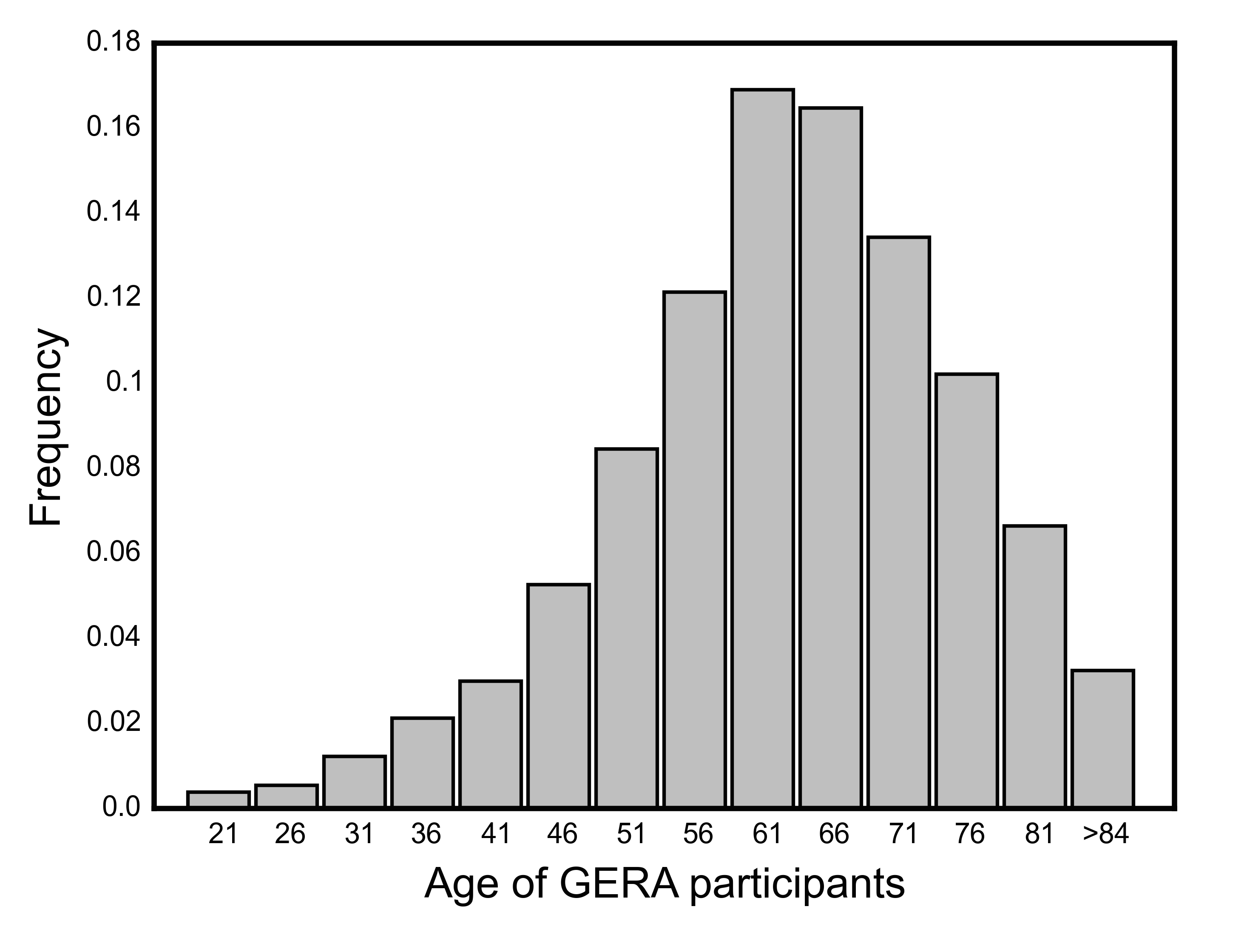

Supplement: S2 Fig — The labels on the x-axis indicate the center of 5-year interval age bins (except the last category). See S1 Data for underlying data. (TIF) [file pbio.2002458.s002.tif]

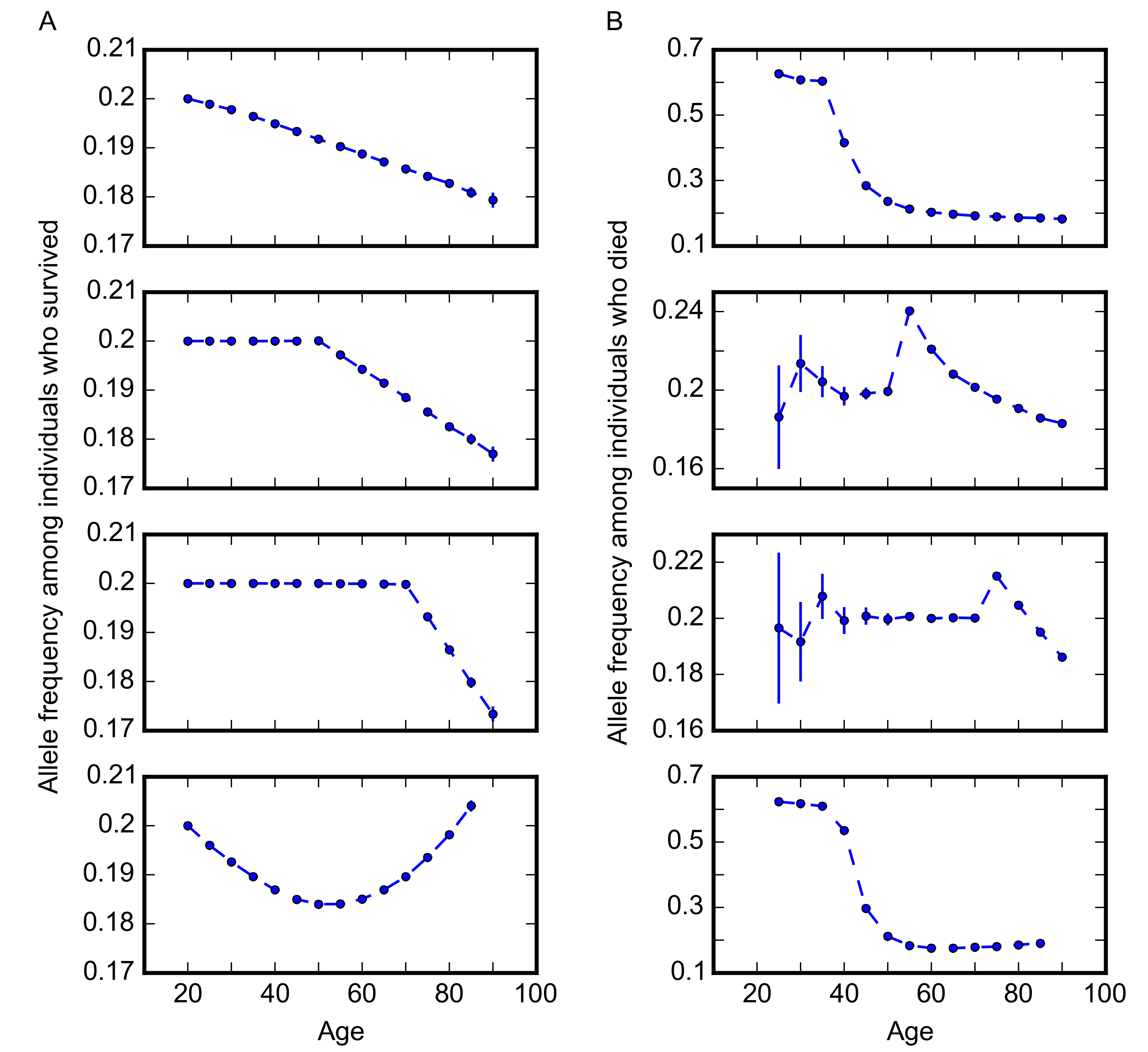

Supplement: S3 Fig — (A) Simulated allele frequencies among surviving individuals, reproducing trends as in Fig 1A. (B) Trends in allele frequency among individuals who died, corresponding to the trends in (A). Points are allele frequencies within 5-year interval age bins (mean ± 2 SE). (TIF) [file pbio.2002458.s003.tif]

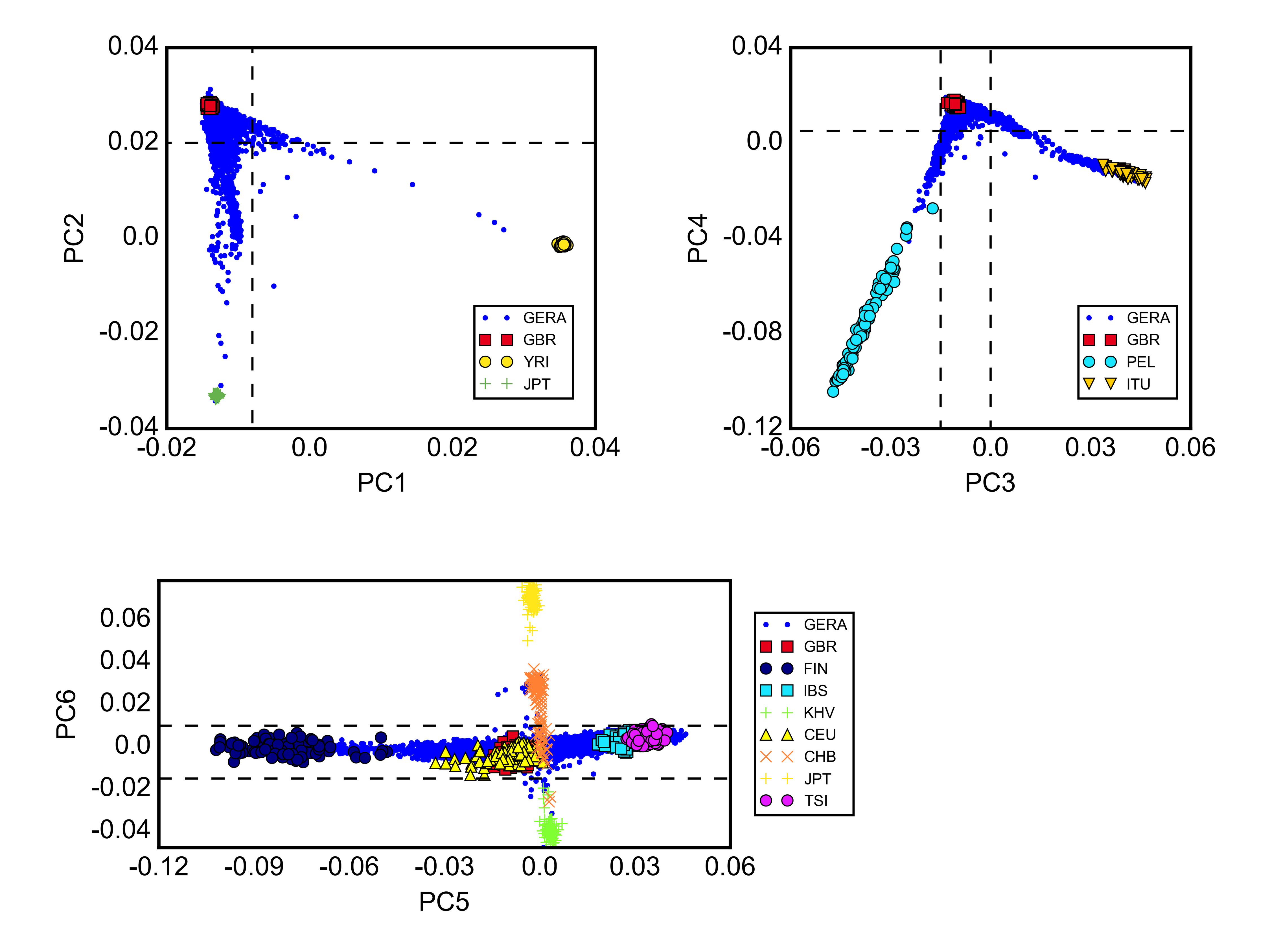

Supplement: S4 Fig — Shown are PCs inferred for all 26 populations in the 1000 Genomes Project phase 3 data. For clarity, in each plot, only a few representative populations are shown. GERA individuals (blue dots) are projected on the inferred PCs. The dashed lines correspond to the dashed lines in S5 Fig, delimiting the majority of GERA individuals. (TIF) [file pbio.2002458.s004.tif]

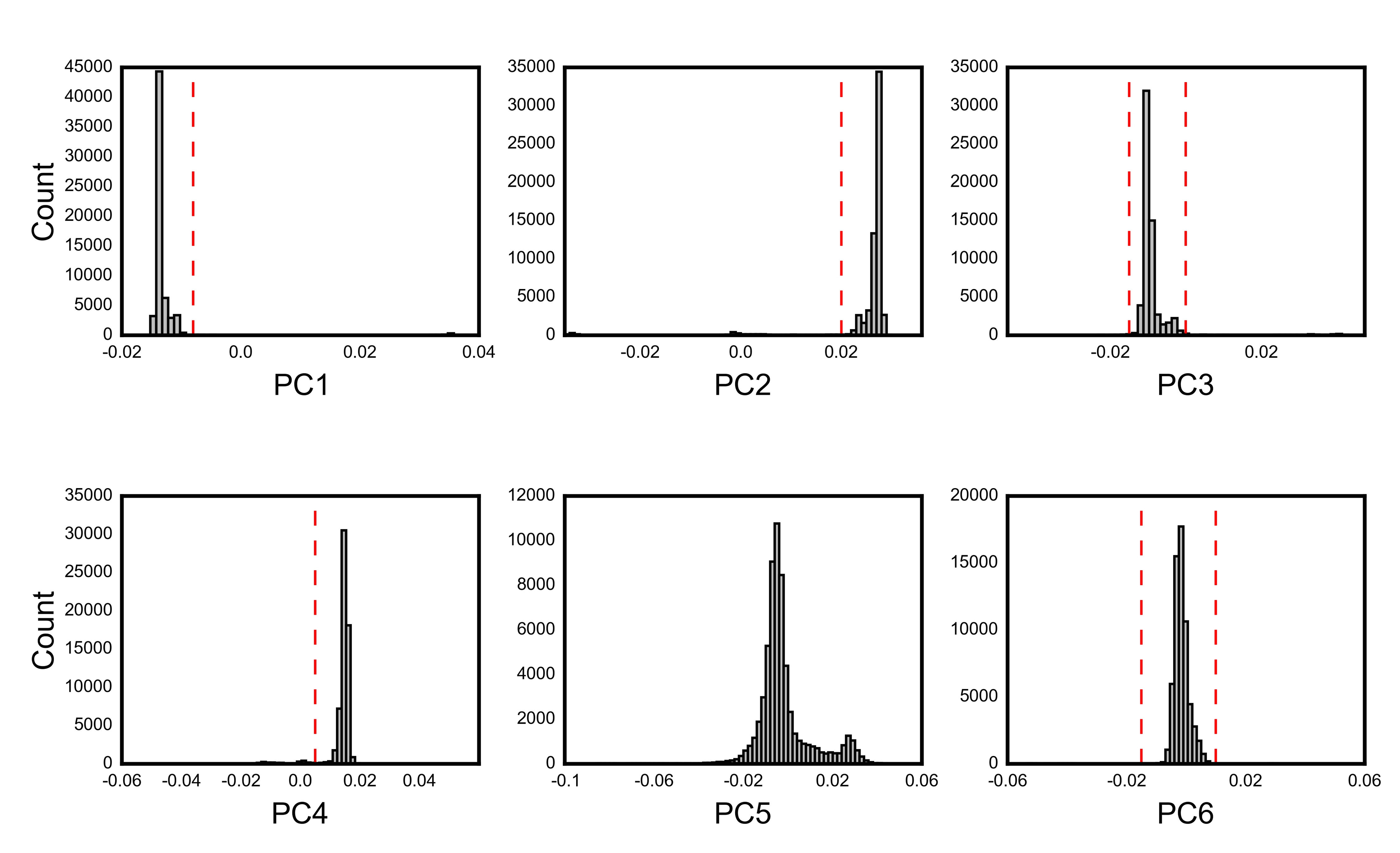

Supplement: S5 Fig — The dashed lines enclose the majority of the data points; beyond, individuals were labeled as “non-Europeans.” (TIF) [file pbio.2002458.s005.tif]

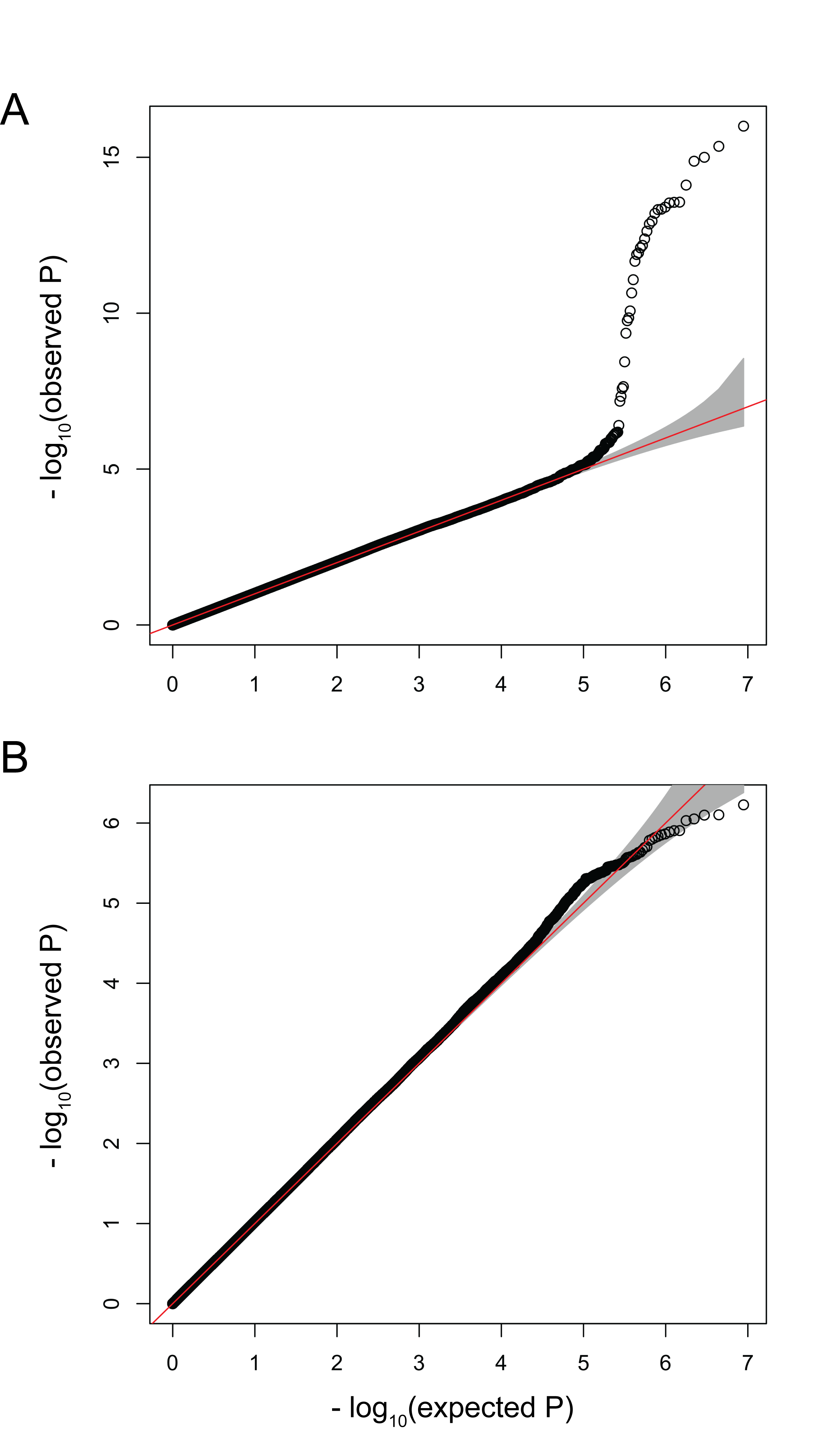

Supplement: S6 Fig — Quantile-quantile plots for age (A) and age by sex (B) effects. The red lines indicate the distribution of the P values under the null model (of no age or age by sex effect) and the shaded bands represent the 95% confidence intervals, assuming independent SNPs. See S1 Data for underlying data. (TIF) [file pbio.2002458.s006.tif]

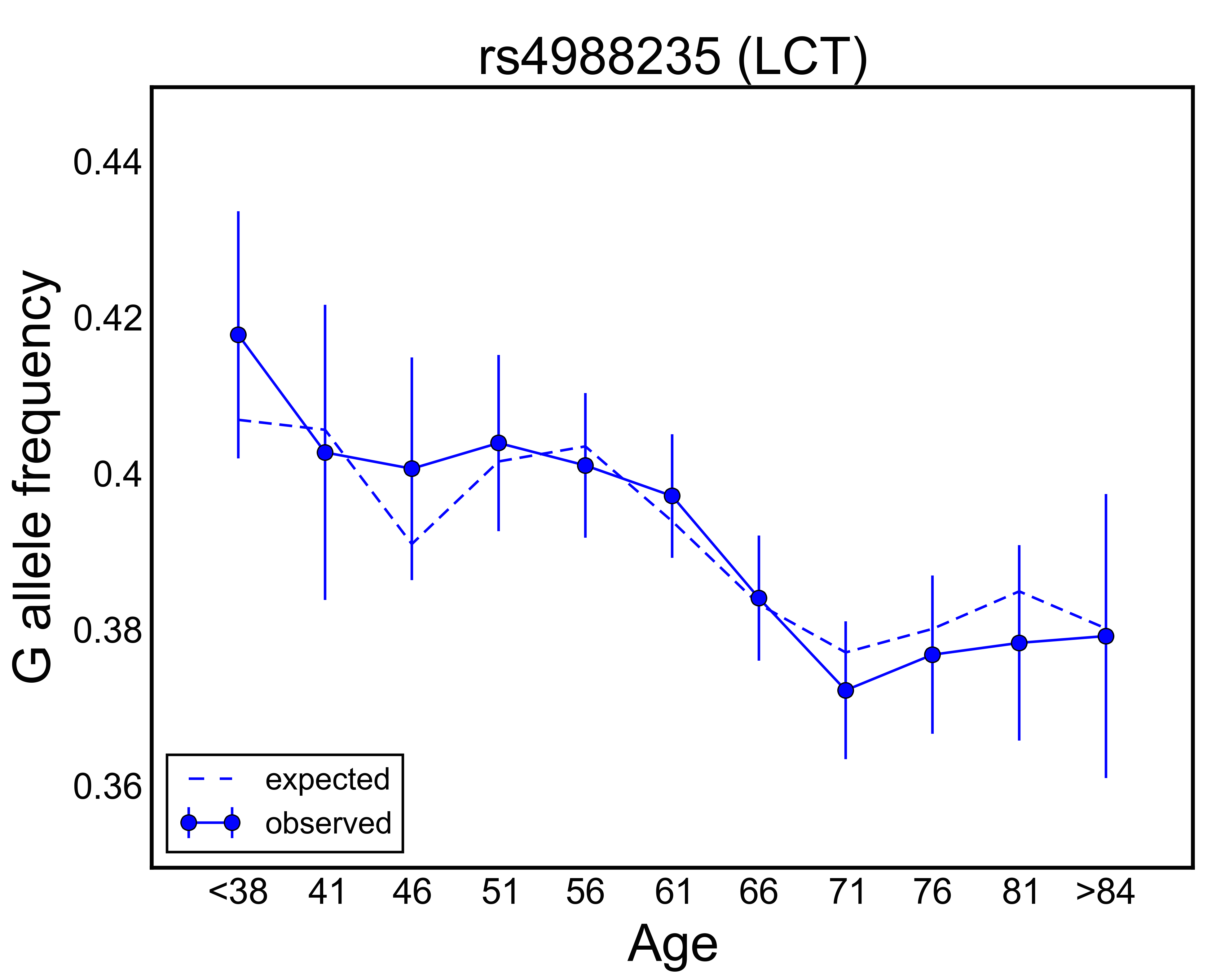

Supplement: S7 Fig — The data points are the frequencies within 5-year interval age bins (± 2 SE). The x-axis indicates the center of the age bin (except for the first and the last bins). Bins with ages below 38 years are merged into 1 bin because of the relatively small sample sizes per bin. The dashed line shows the expected frequency based on the null model, accounting for confounding batch effects and, importantly, changes in ancestry. See S1 Data for underlying data. (TIF) [file pbio.2002458.s007.tif]

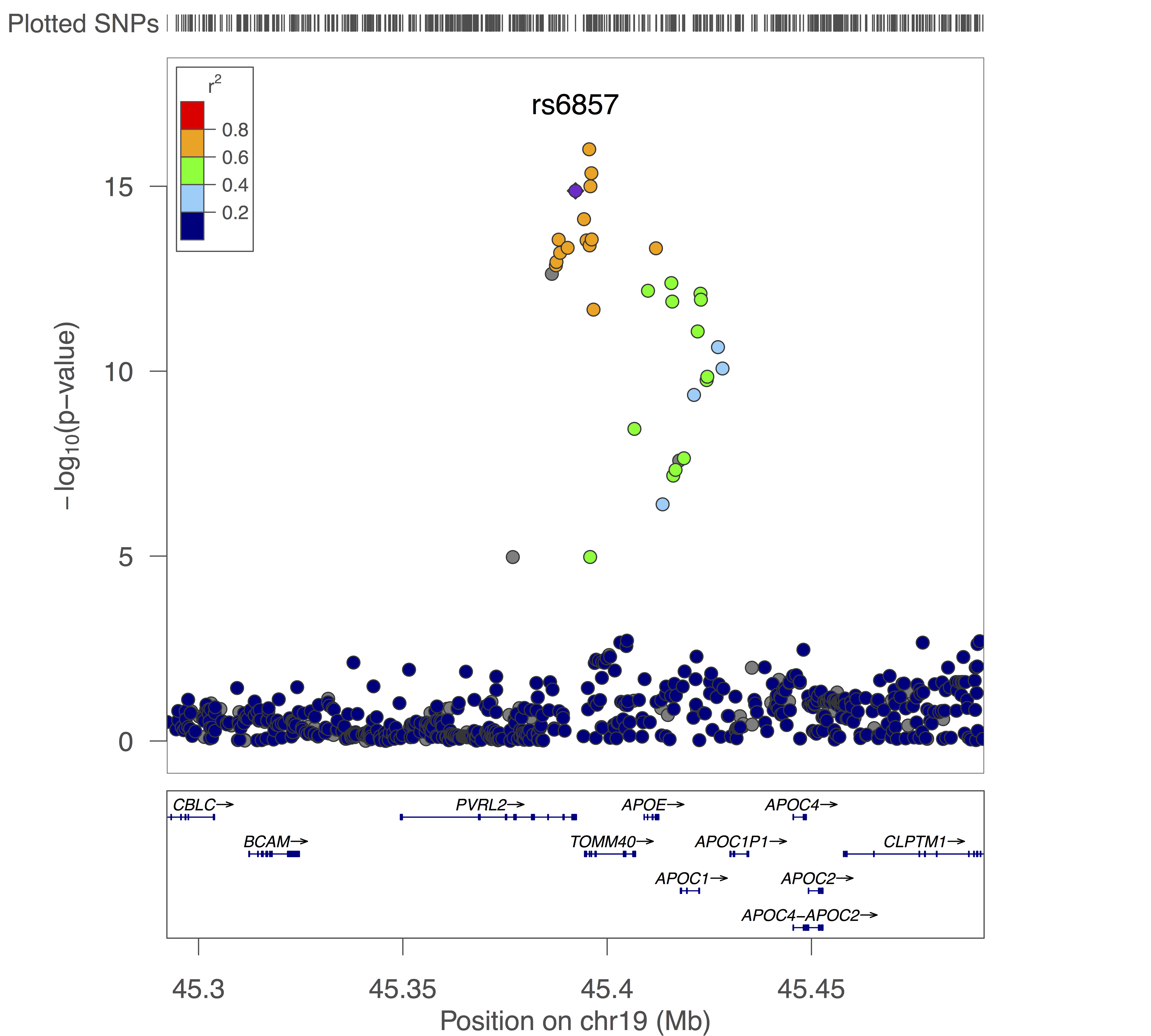

Supplement: S8 Fig — The y-axis shows P values obtained from a test of the influence of single genetic variants on age-specific mortality in GERA. (TIF) [file pbio.2002458.s008.tif]

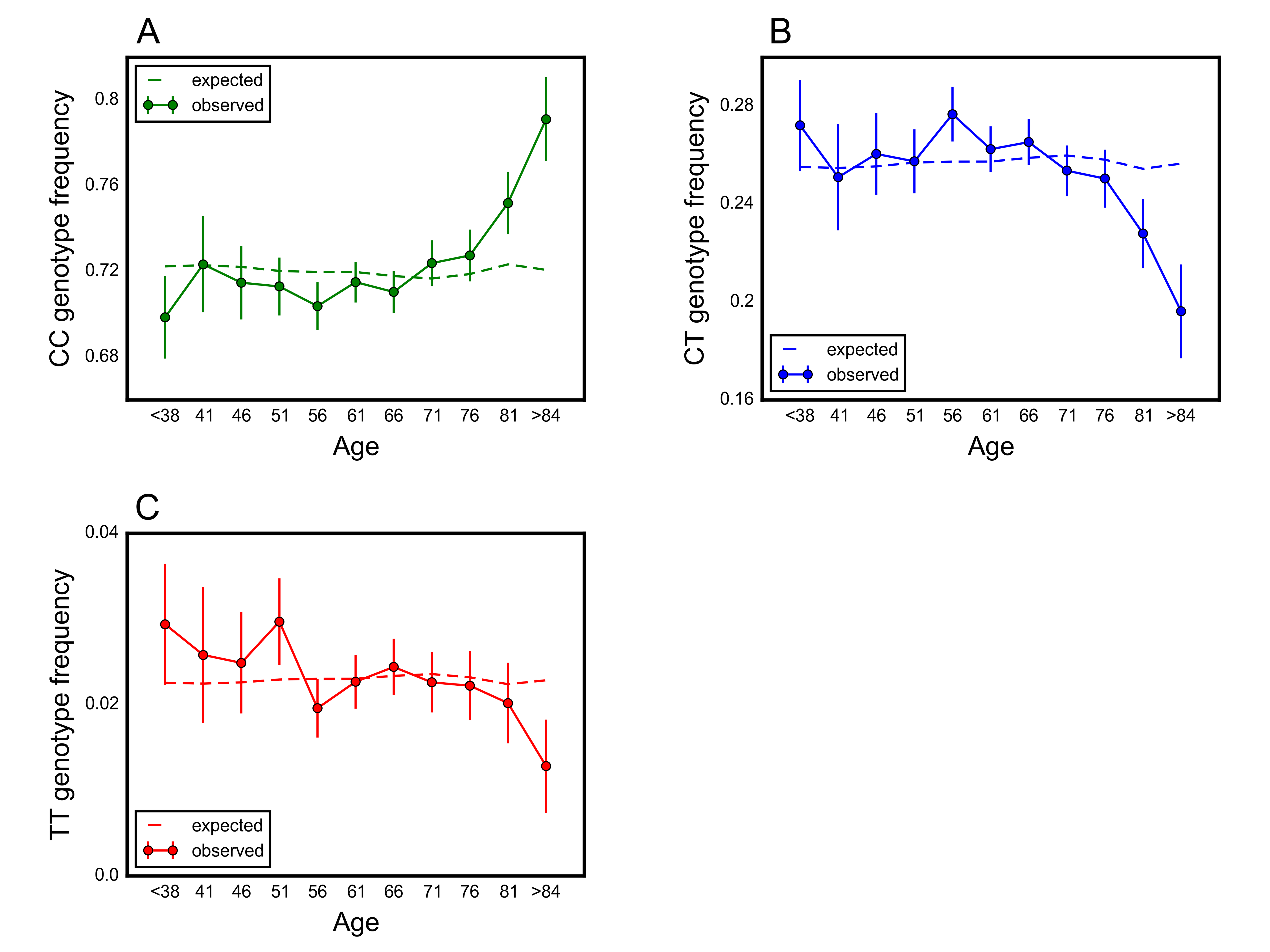

Supplement: S9 Fig — Frequency of noncarriers (A), heterozygous (B), and homozygous (C) carriers of the risk allele for rs6857, tagging the ε4 allele of the APOE gene, across GERA age bins. Data points are frequencies within 5-year interval age bins (± 2 SE), with the center of the bin indicated on the x-axis (except for the first and the last bins). Bins with ages below 38 years are merged into 1 bin because of the relatively small sample sizes per bin. The dashed line shows the expected frequency based on the null model, accounting for confounding batch effects and changes in ancestry. See S1 Data for underlying data. (TIF) [file pbio.2002458.s009.tif]

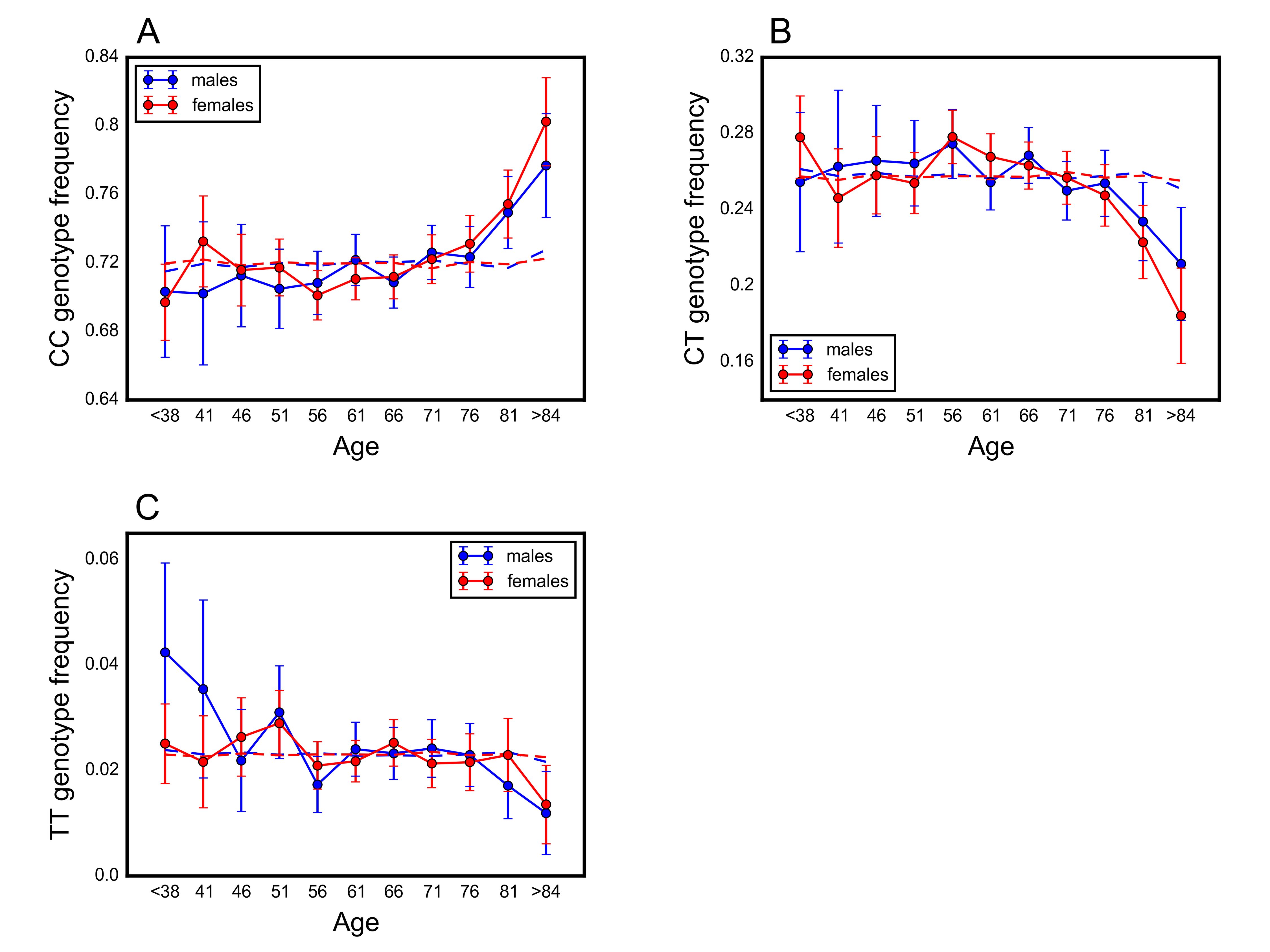

Supplement: S10 Fig — Frequency of noncarriers (A), heterozygous (B), and homozygous (C) carriers of the risk allele for rs6857, tagging the ε4 allele of the APOE gene, across GERA age bins. Data points are frequencies within 5-year interval age bins (± 2 SE), with the center of the bin indicated on the x-axis (except for the first and the last bins). Bins with ages below 38 years are merged into 1 bin because of the relatively small sample sizes per bin. The dashed line shows the expected frequency based on the null model, accounting for confounding batch effects and changes in ancestry. See S1 Data for underlying data. (TIF) [file pbio.2002458.s010.tif]

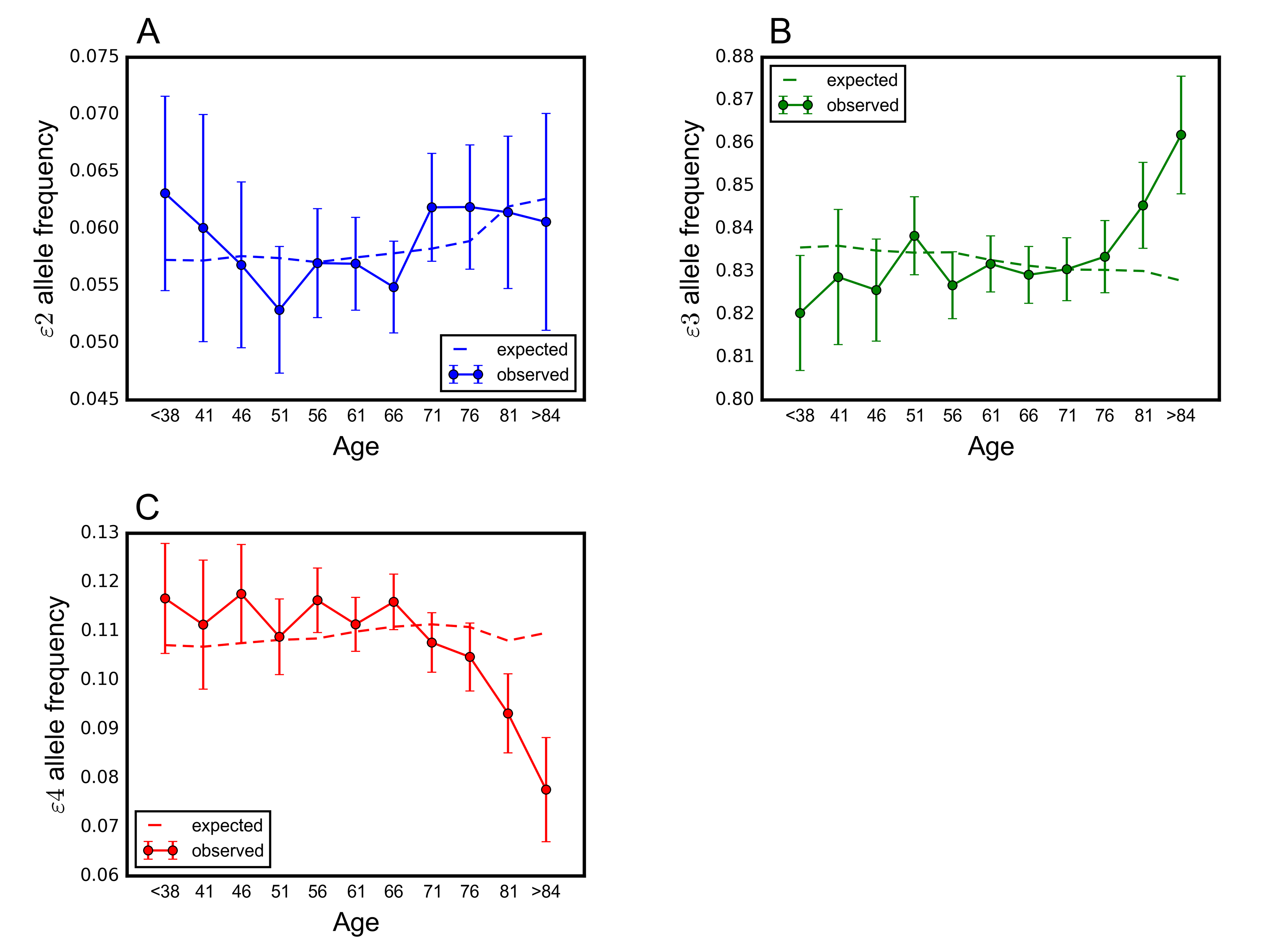

Supplement: S11 Fig — Frequency of the ε2 (A), ε3 (B), and ε4 (C) alleles across GERA age bins. Data points are frequencies within 5-year interval age bins (± 2 SE), with the center of the bin indicated on the x-axis (except for the first and the last bins). Bins with ages below 38 years are merged into 1 bin because of the relatively small sample sizes per bin. The dashed line shows the expected frequency based on the null model, accounting for confounding batch effects and changes in ancestry. See S1 Data for underlying data. (TIF) [file pbio.2002458.s011.tif]

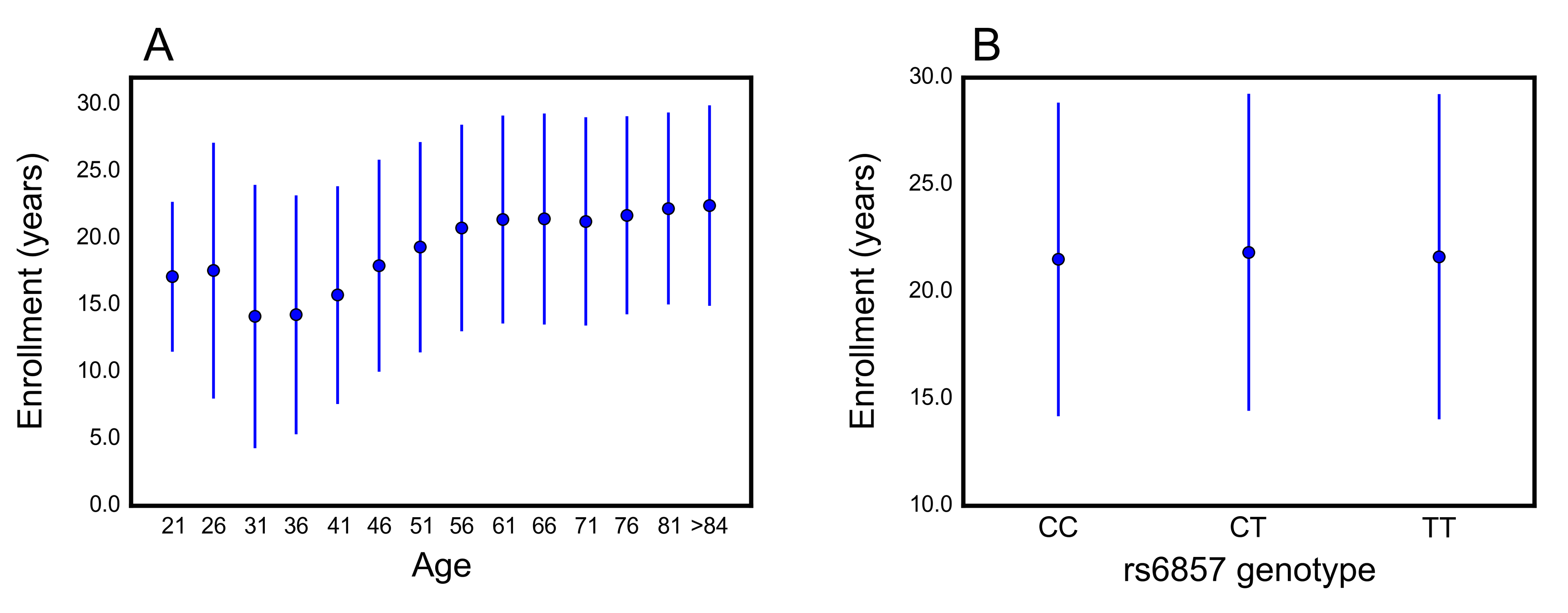

Supplement: S12 Fig — (A) Years enrolled in the care plan at the time of the survey (mean ± SD) per age bin. The x-axis indicates the center of 5-year interval age bins (except the last category). (B) Years enrolled in the plan (mean ± SD) for individuals >70 years old versus the rs6857 (APOE) genotype that they carry. See S1 Data for underlying data. (TIF) [file pbio.2002458.s012.tif]

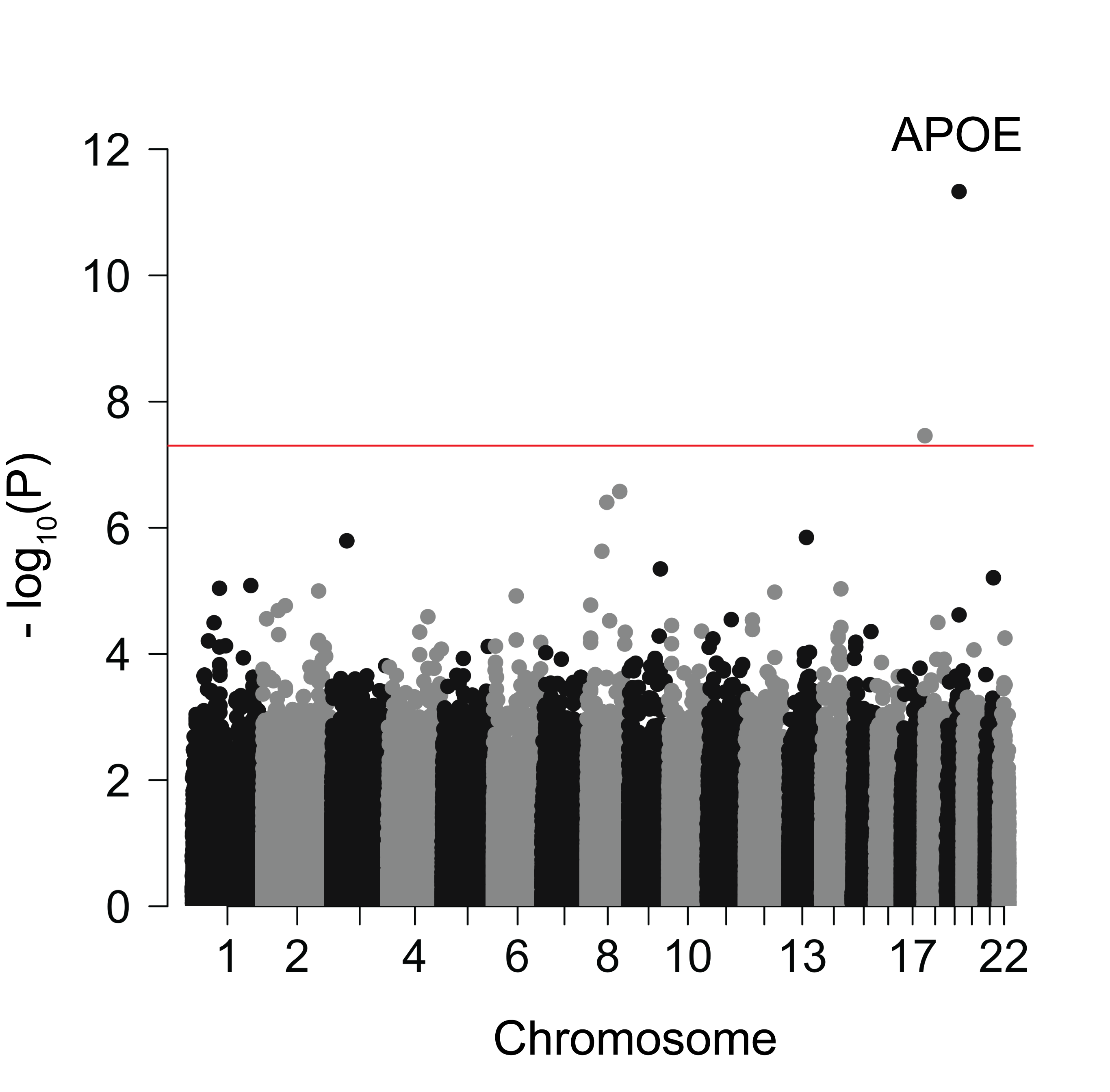

Supplement: S13 Fig — Manhattan plot of P values testing for a change in allele frequency with age using the version of the model with age treated as an ordinal variable. The plot only includes the filtered genotyped SNPs in the GERA study. Red line marks the P = 5 × 10^−8^ threshold. The signal for variant on chromosome 18 is presumably caused by genotyping error, as other closely linked variants did not show a similar behavior, and the signal was lost when the variant was imputed using a leave-one-out approach. See S1 Data for underlying data. (TIF) [file pbio.2002458.s013.tif]

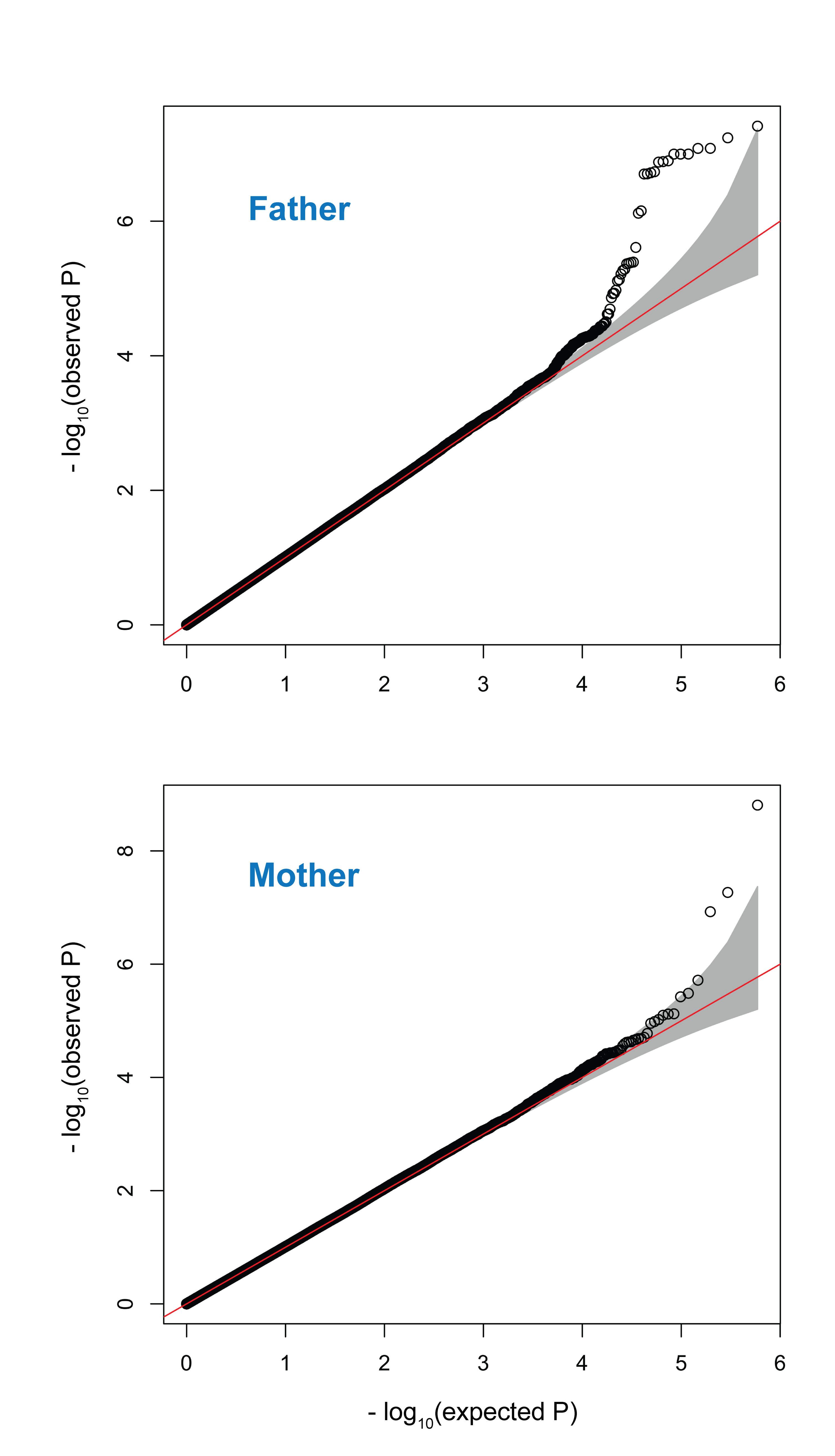

Supplement: S14 Fig — Quantile-quantile plots for significant change in allele frequency with father’s (A) and mother’s (B) age at death. The red lines indicate distribution of the P values under the null (no change in frequency) and the shaded bands represent the 95% confidence intervals, assuming independent SNPs. See S2 Data for underlying data. (TIF) [file pbio.2002458.s014.tif]

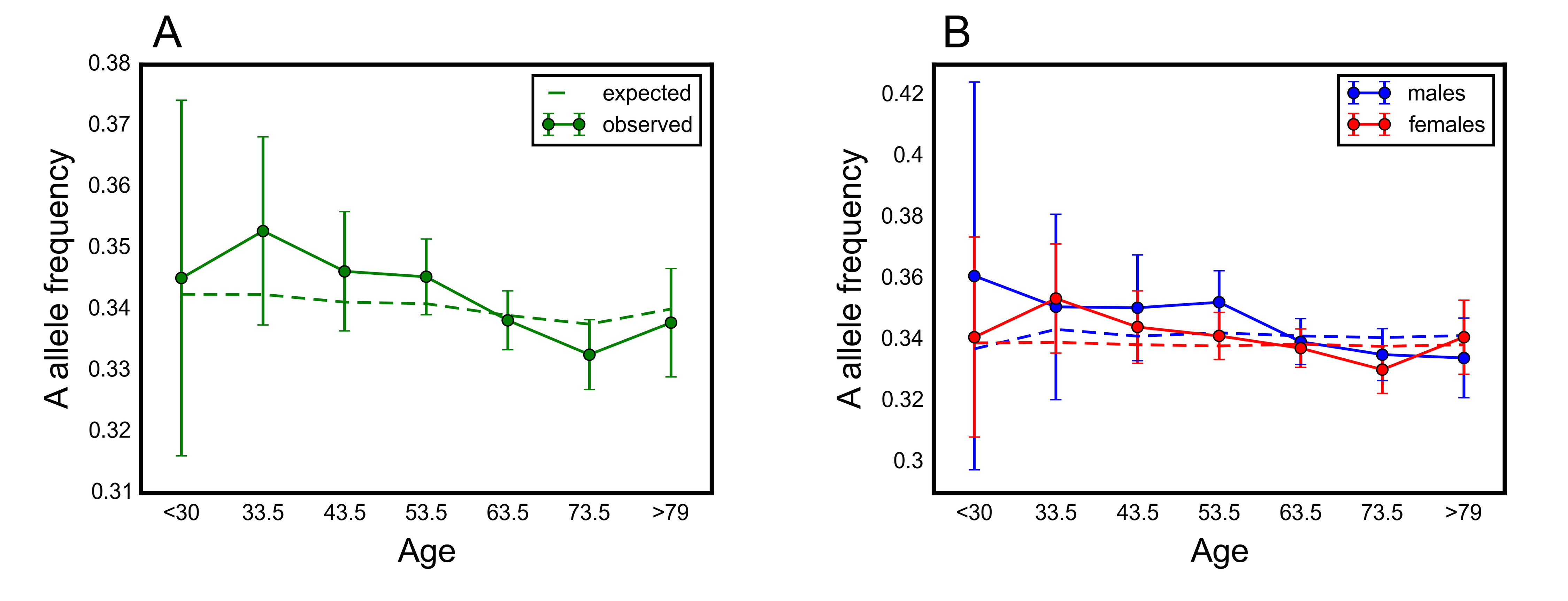

Supplement: S15 Fig — Allele frequency trajectory of rs1051730 with age for males and females together (A) and separately (B). The data points are the frequencies within 10-year interval age bins (± 2 SE). The x-axis indicates the center of the age bin (except for the first and the last bins). The dashed line shows the expected frequency based on the null model, accounting for confounding batch effects and changes in ancestry. See S1 Data for underlying data. (TIF) [file pbio.2002458.s015.tif]

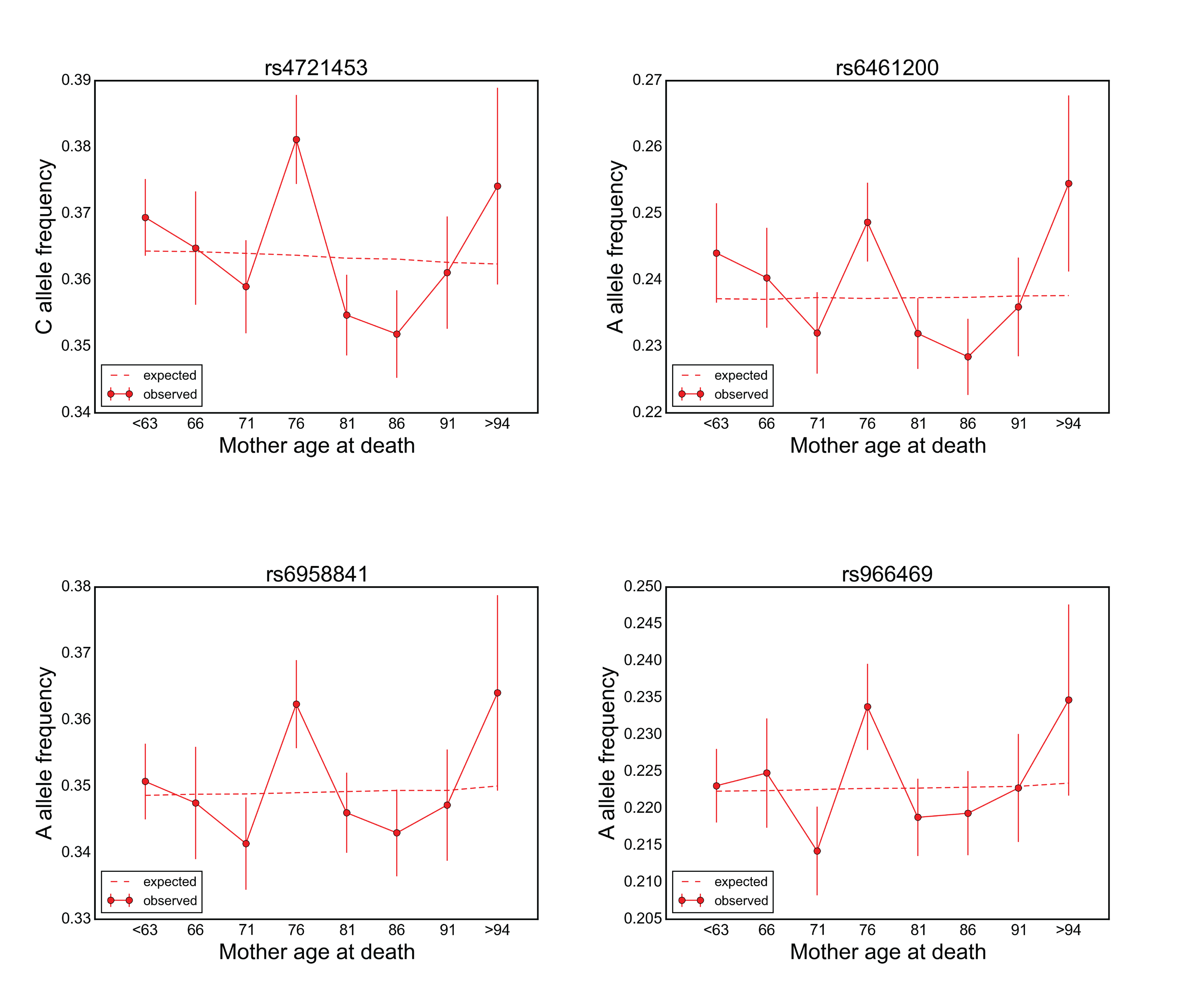

Supplement: S16 Fig — Plots are for 4 genotyped SNPs in moderate linkage disequilibrium with P < 10−4 for the change in allele frequency with mother’s age at death. Data points are frequencies within 5-year interval age bins (± 2 SE), with the center of the bin indicated on the x-axis (except for the first and the last bins). The dashed line shows the expected frequency based on the null model, accounting for confounding batch effects and changes in ancestry. See S2 Data for underlying data. (TIF) [file pbio.2002458.s016.tif]

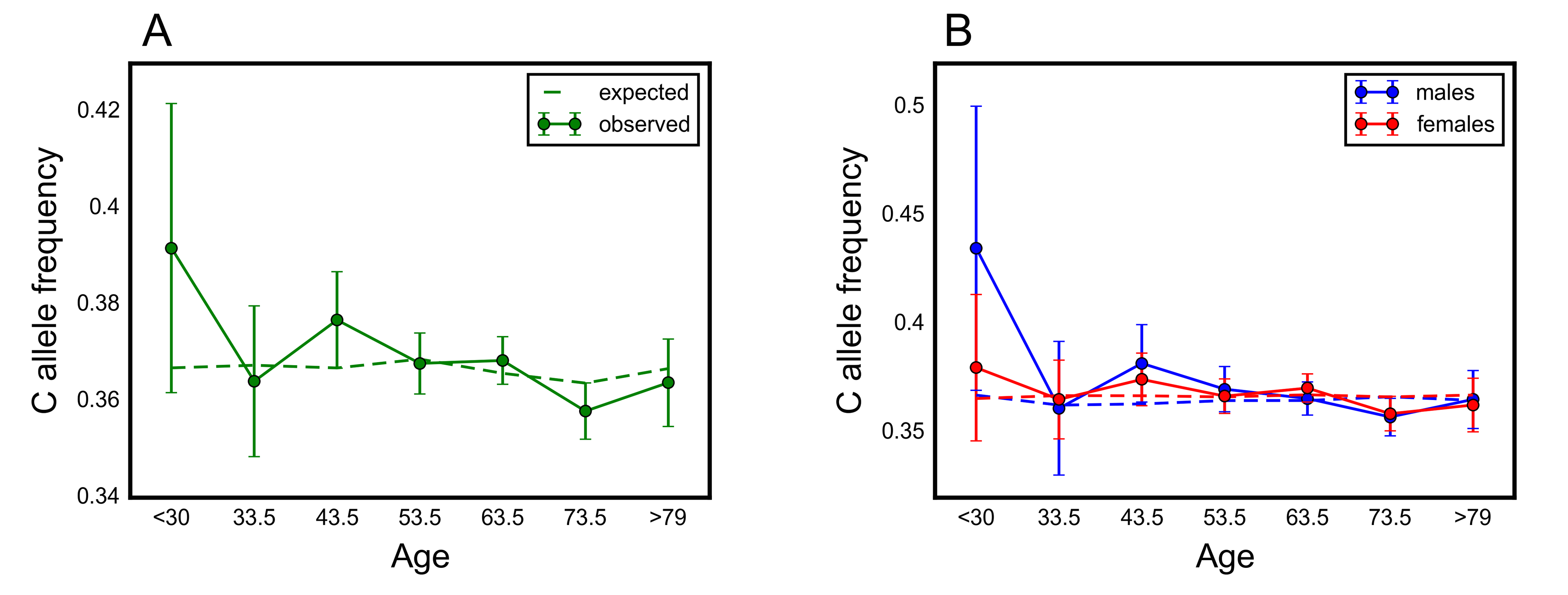

Supplement: S17 Fig — Allele frequency trajectory of rs4721453 with age for males and females together (A) and separately (B). The data points are the frequencies within 10-year interval age bins (± 2 SE). The x-axis indicates the center of the age bin (except for the first and the last bins). The dashed line shows the expected frequency based on the null model, accounting for confounding batch effects and changes in ancestry. See S1 Data for underlying data. (TIF) [file pbio.2002458.s017.tif]

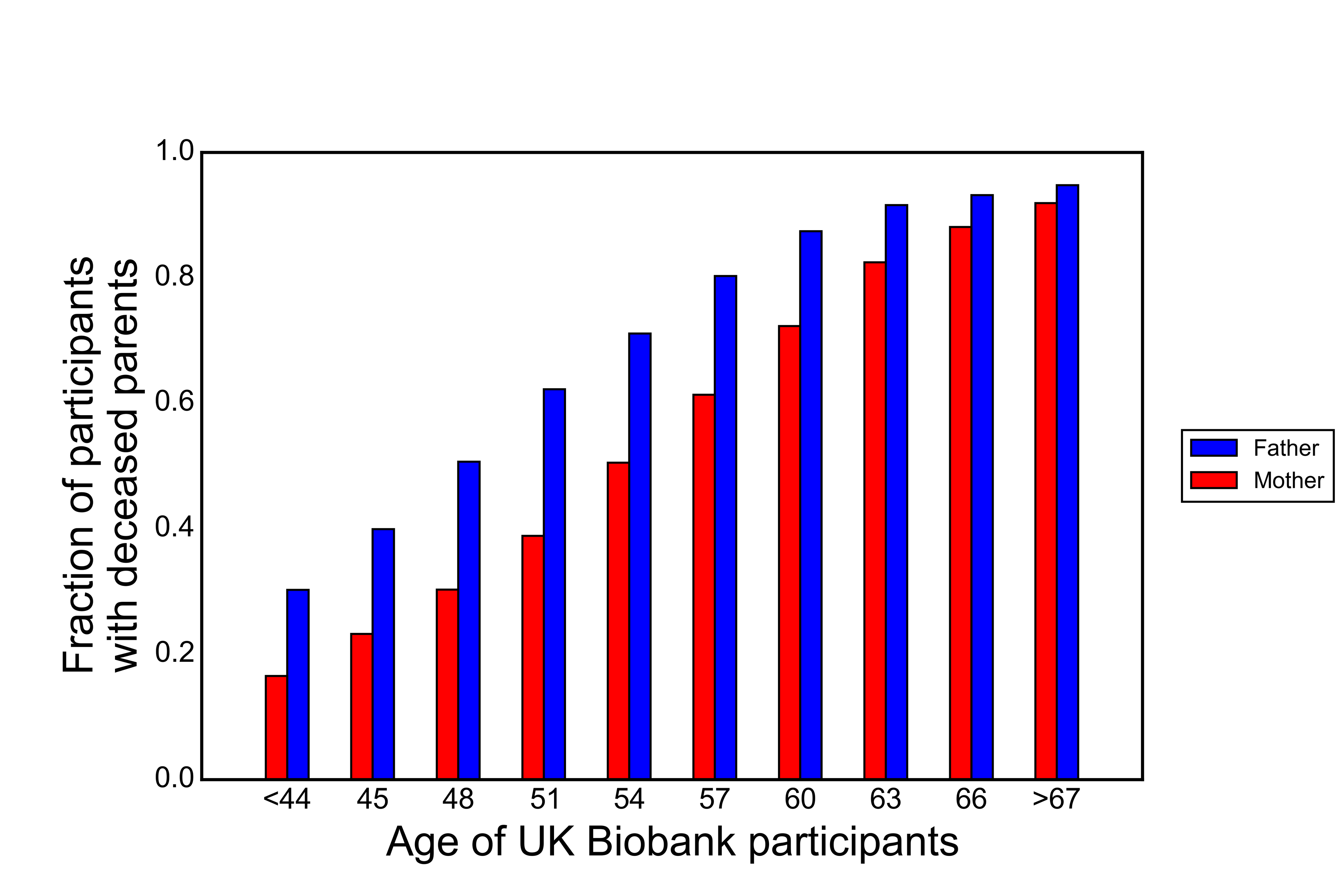

Supplement: S18 Fig — Fraction of the participants in each age bin (bin size of 3 years) who reported their father’s or mother’s age at death. See S2 Data for underlying data. (TIF) [file pbio.2002458.s018.tif]

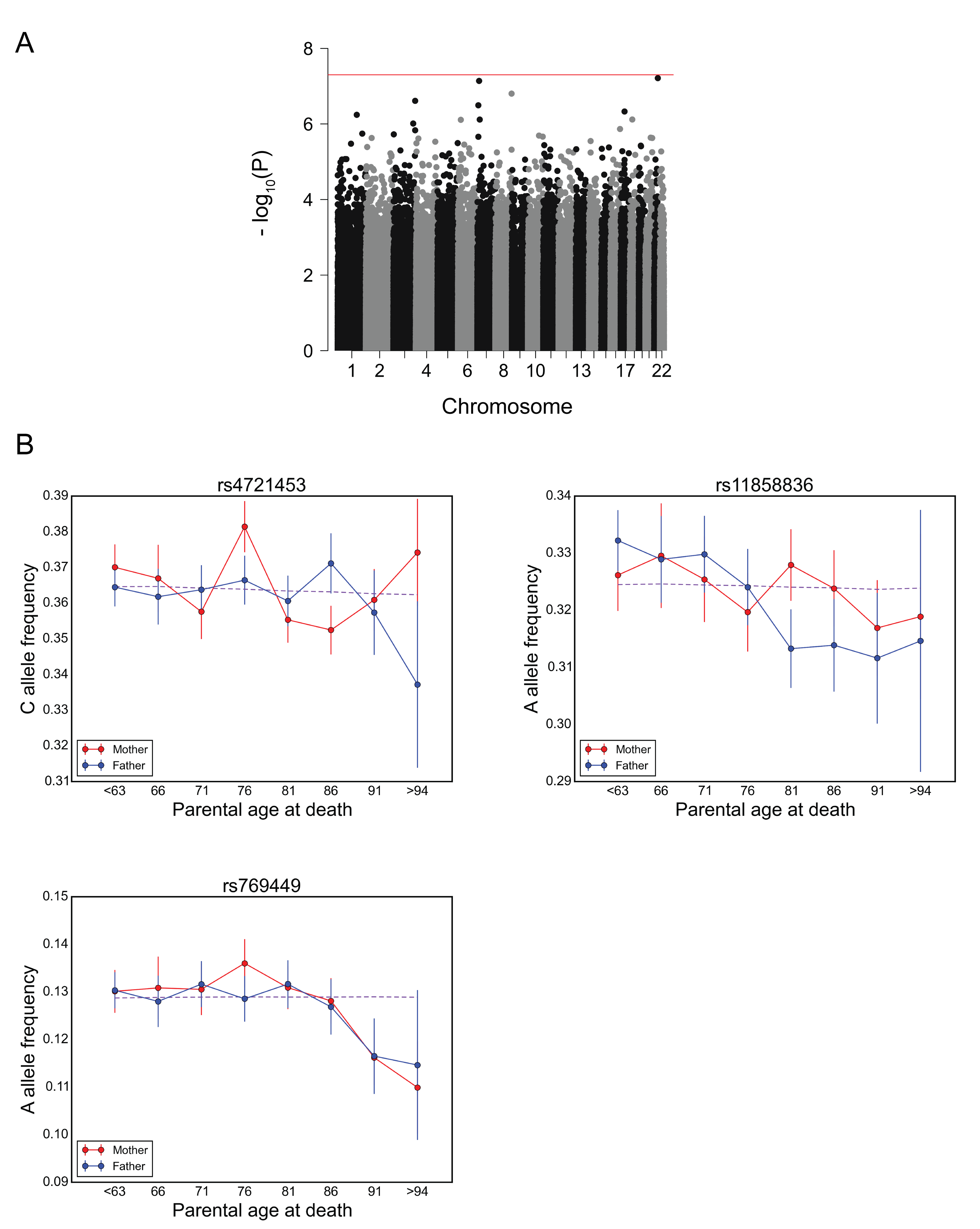

Supplement: S19 Fig — (A) Manhattan plot of P values, testing a difference between fathers and mothers in the change in allele frequency with parental age at death. (B) Allele frequencies as a function of father’s and mother’s age at death for top SNPs with age effects: rs4721453 (near MEOX2), rs11858836 (near CHRNA3), and rs769449 (APOE). The data points are the frequencies within 5-year interval age bins (± 2 SE). The x-axis indicates the center of the age bin (except for the first and the last bins). The dashed line shows the expected frequency based on the null model, accounting for confounding batch effects and changes in ancestry. See S2 Data for underlying data. (TIF) [file pbio.2002458.s019.tif]

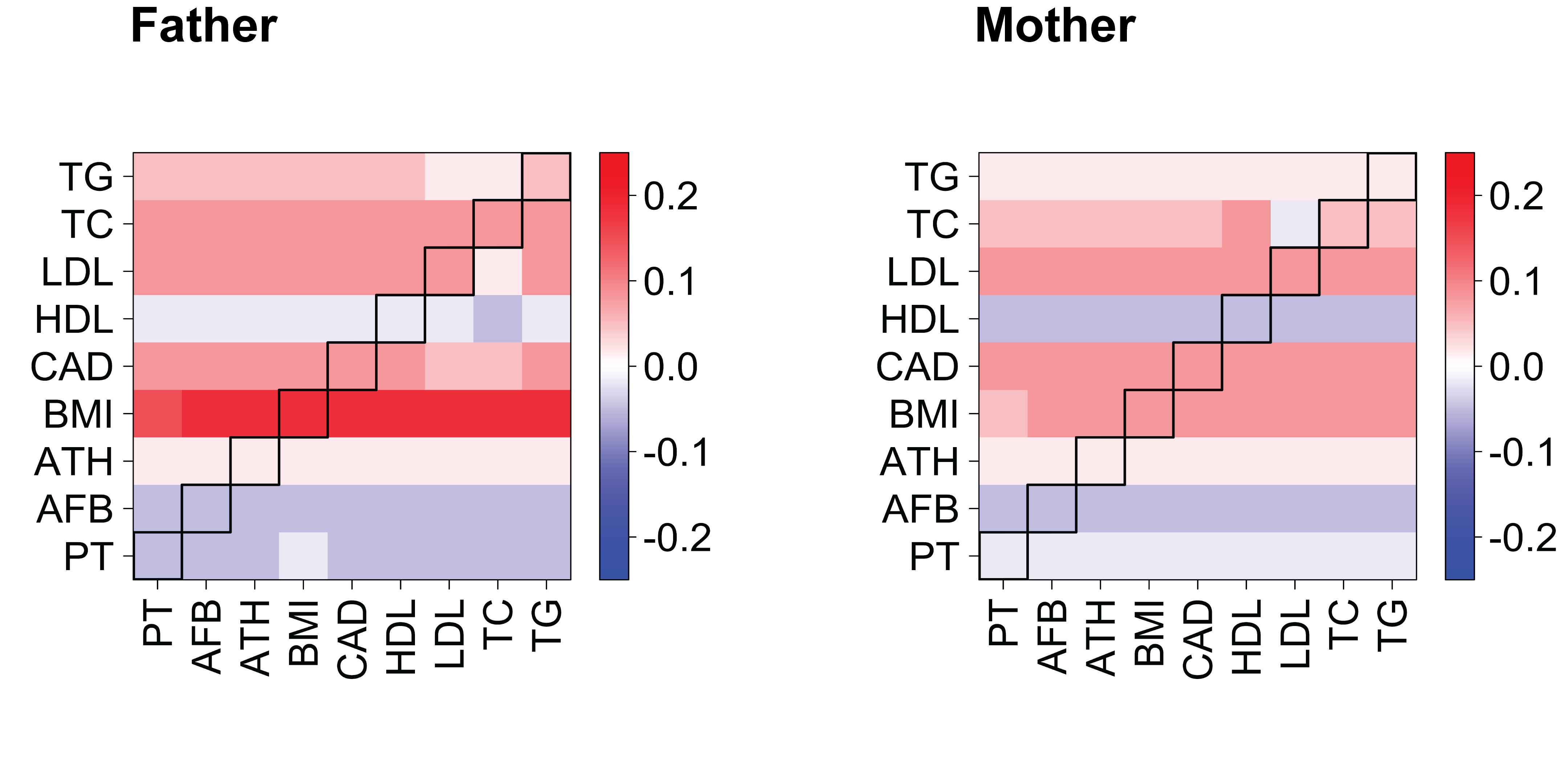

Supplement: S20 Fig — Each square [i,j] shows the effect size (log[hazard ratio]) of the polygenic score for trait i on father’s (left) or mother’s (right) survival in the Cox model, after accounting for the effect of the polygenic score of trait j (i.e., incorporating the polygenic score for trait j as a covariate in the null model, see Materials and methods). Squares on the diagonal (marked by black rectangles) show the effect size of the polygenic score without accounting for the score for other traits. See S2 Data for underlying data. (TIF) [file pbio.2002458.s020.tif]

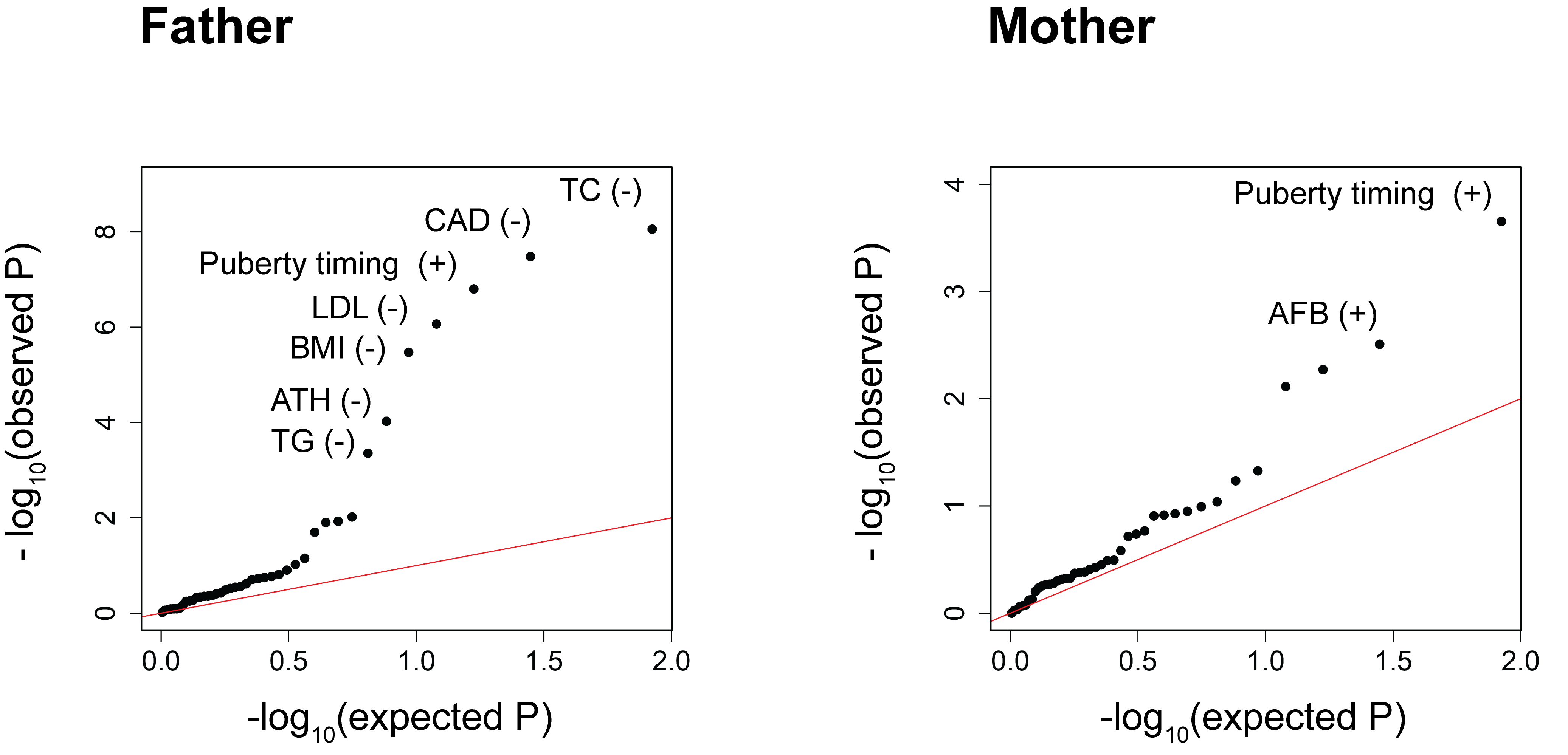

Supplement: S21 Fig — Quantile-quantile plots for changes in polygenic score of 42 traits (see S1 Table) with father’s (A) or mother’s (B) age at death, after accounting for confounding batch effects, changes in ancestry, and the participant’s age, sex, year of birth, and the Townsend index (a measure of socioeconomic status). The red lines indicate the distribution of the P values under the null model. Signs “+” and “−” indicate protective and deleterious effects associated with higher values of polygenic scores, respectively. See S2 Data for underlying data. (TIF) [file pbio.2002458.s021.tif]

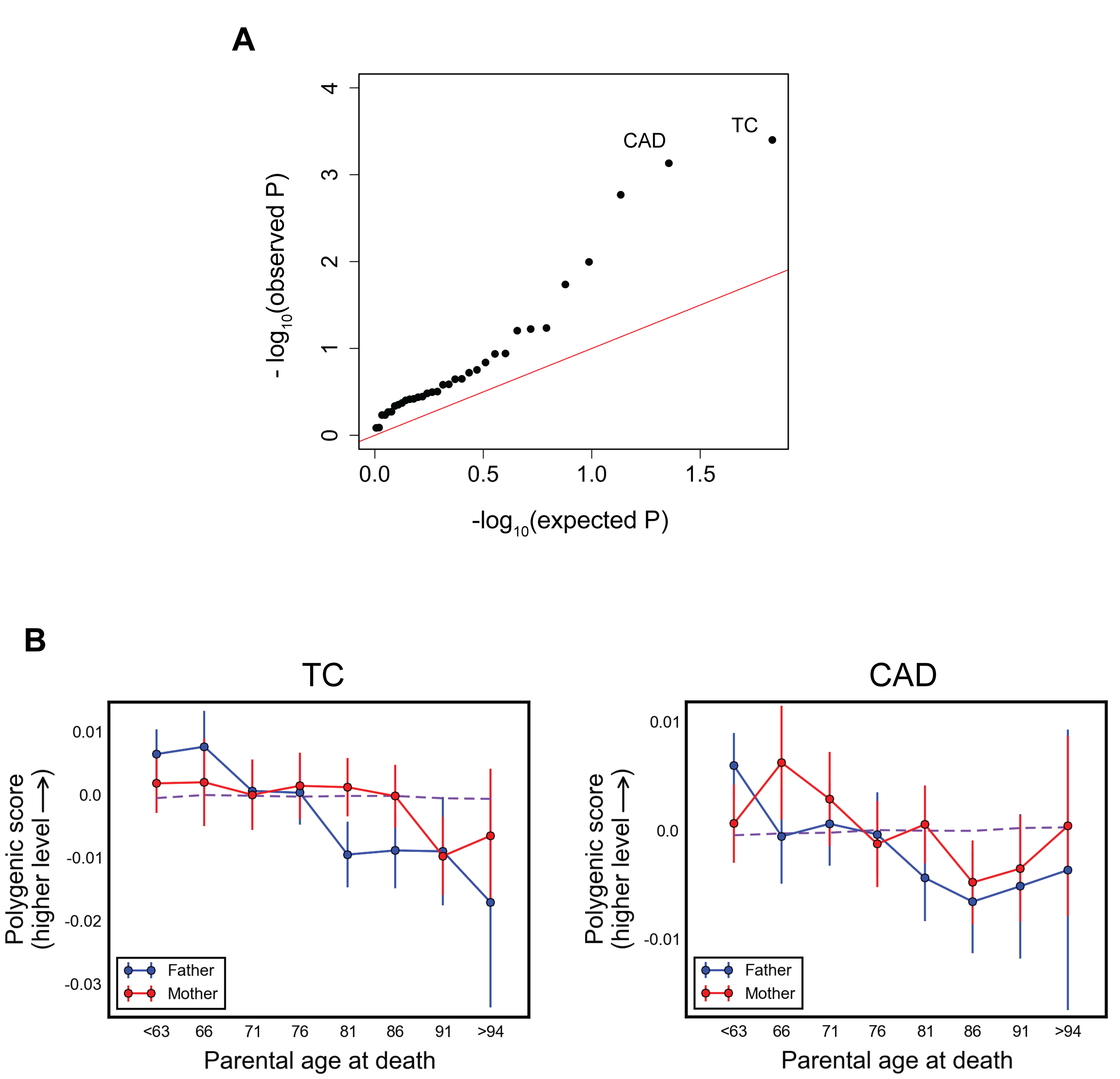

Supplement: S22 Fig — (A) Quantile-quantile plot for changes in polygenic score of 42 traits (see S1 Table) with parental ages at death that are different between fathers and mothers of the UK Biobank participants. The red lines indicate the distribution of the P values under the null. (B) The trend in polygenic score with parental ages at death for total cholesterol and coronary artery disease, which show significant age by sex effects. The data points are the mean polygenic scores within 5-year interval age bins (± 2 SE). The x-axis indicates the center of the age bin (except for the first and the last bins). The dashed line shows the expected polygenic score based on the null model, accounting for confounding batch effects, changes in ancestry, and the participant’s age, sex, year of birth, and the Townsend index (a measure of socioeconomic status). See S2 Data for underlying data. (TIF) [file pbio.2002458.s022.tif]

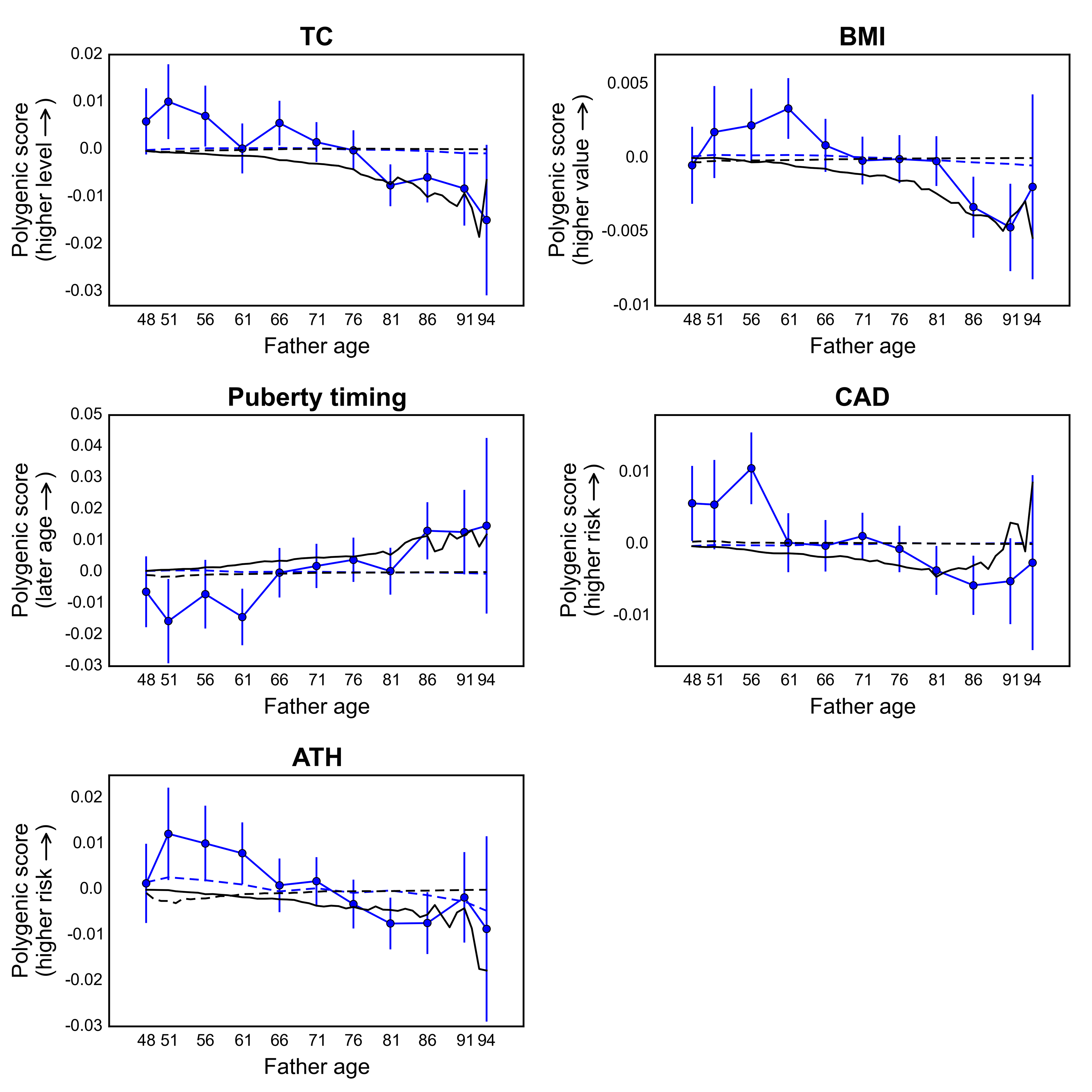

Supplement: S23 Fig — Each plot shows (in blue) the mean polygenic score (± 2 SE) among the fathers who died in a 5-year interval centered around the plotted discs, and (in black) the mean polygenic score among fathers alive up to a given age, i.e., all fathers with age or age at death (if deceased) exceeding a given age. The dashed lines show the expected changes in polygenic scores based on the null model. If there is no effect of the score on survival at a given time (age), then the score among those who died (blue disc) should be the same as the score among those who were alive at the previous time interval. Thus, the divergence between the blue and the black lines in any time interval is an indicator of the effect of the score on survival (and its direction) within that interval. The precise effect, however, also depends on the total hazard rate of the sample, which varies by age. See S2 Data for underlying data. (TIF) [file pbio.2002458.s023.tif]

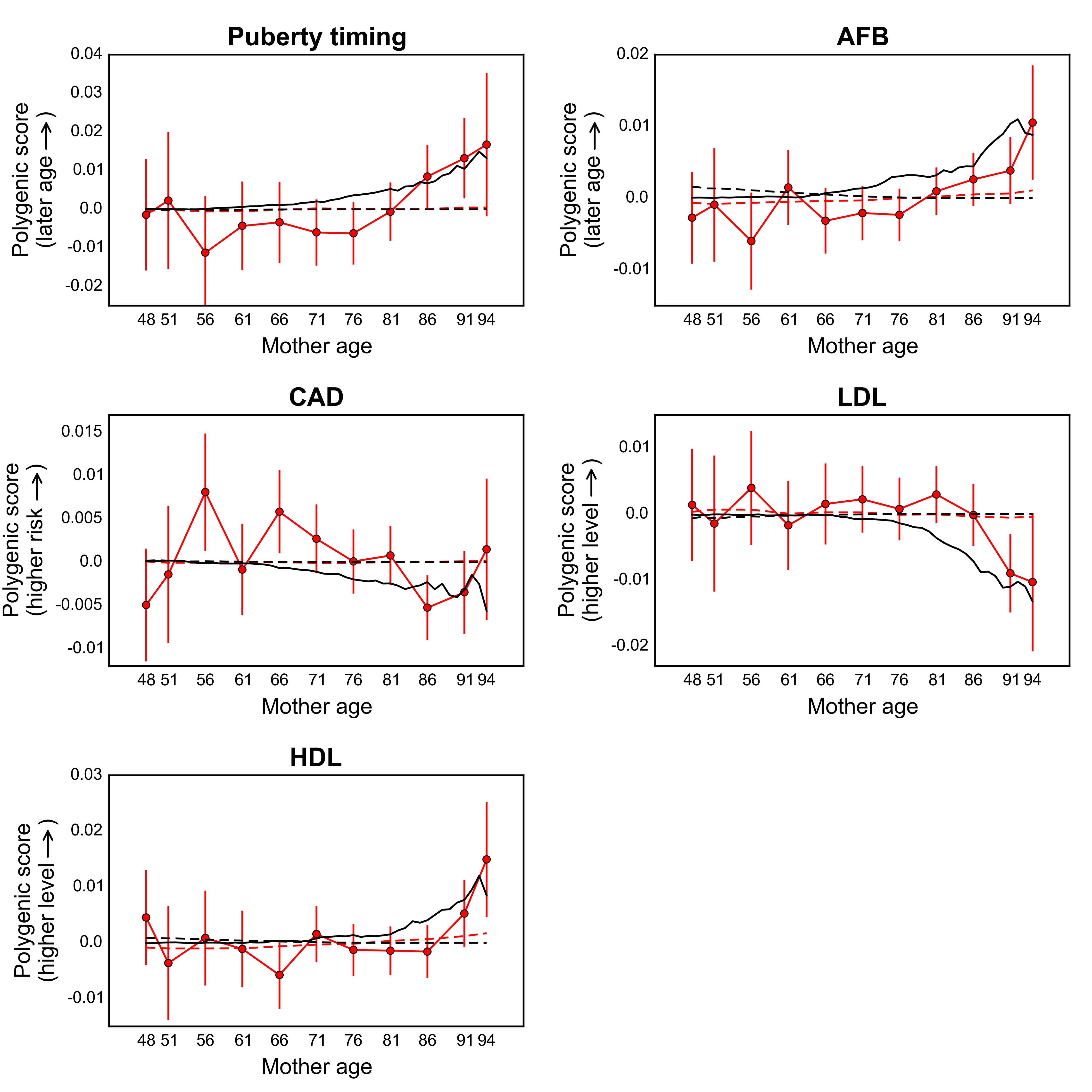

Supplement: S24 Fig — Same as S23 Fig, but plotted for mothers (with red instead of blue). See S2 Data for underlying data. (TIF) [file pbio.2002458.s024.tif]

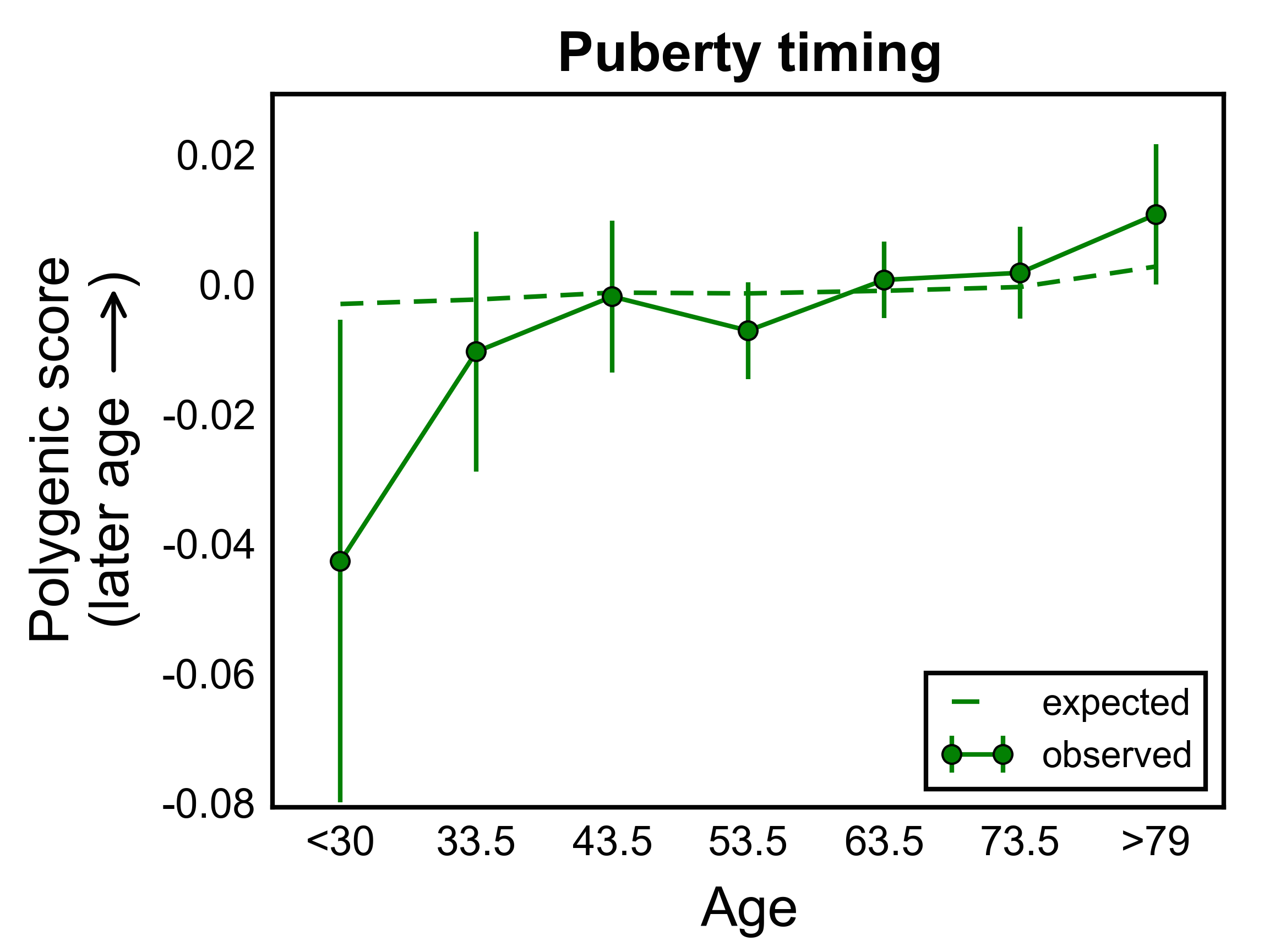

Supplement: S25 Fig — Polygenic score for puberty timing with age of the participants. The data points are the mean scores within 10-year interval age bins (± 2 SE). The x-axis indicates the center of the age bin (except for the first and the last bins). The dashed line shows the expected score based on the null model, accounting for confounding batch effects and changes in ancestry. See S1 Data for underlying data. (TIF) [file pbio.2002458.s025.tif]

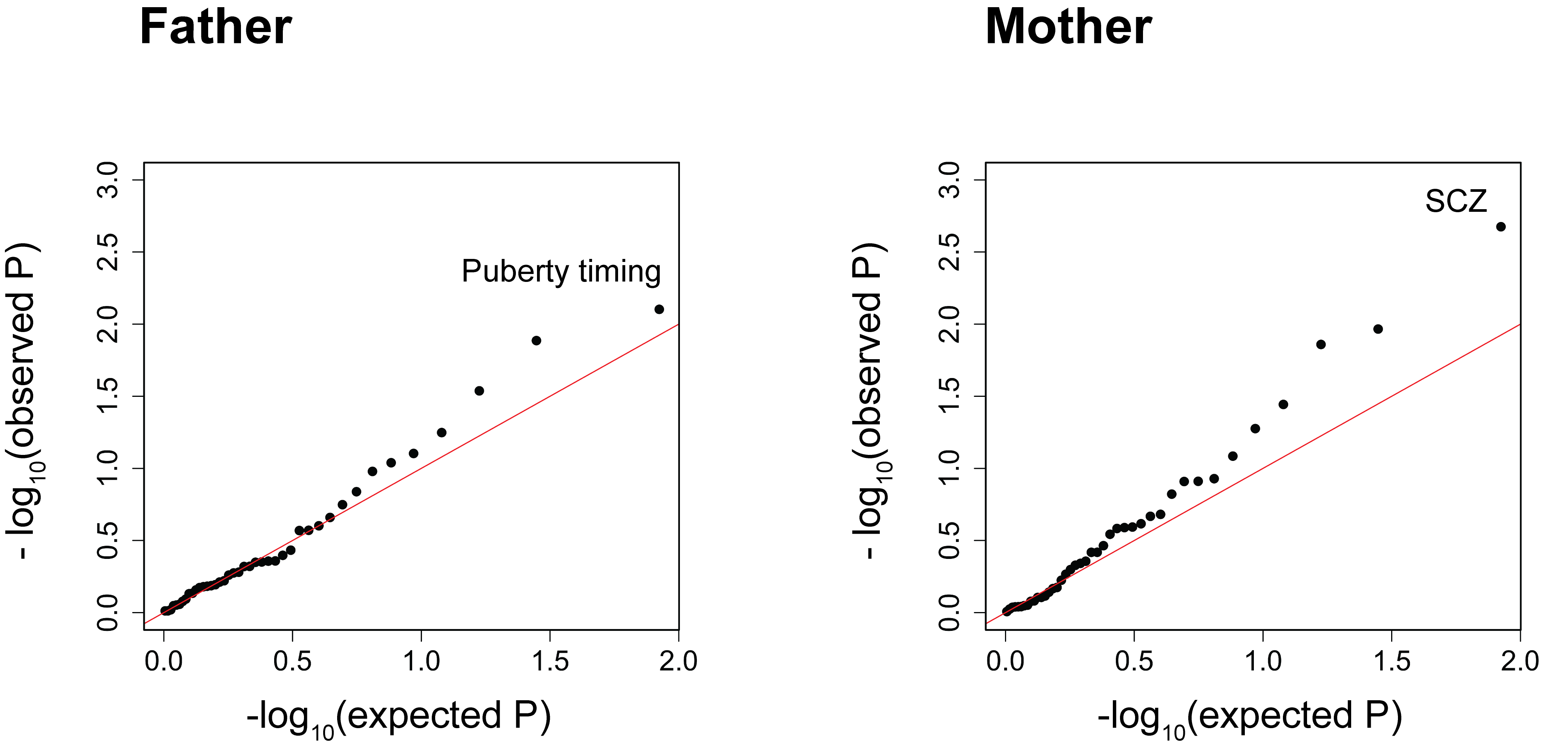

Supplement: S26 Fig — Quantile-quantile plots testing for a change in the squared difference of polygenic score from the mean with fathers’ (A) and mothers’ (B) age at death, treating age variables as ordinal. 42 traits were tested (see S1 Table). The red line indicates the distribution of the P values under the null model. See S2 Data for underlying data. (TIF) [file pbio.2002458.s026.tif]

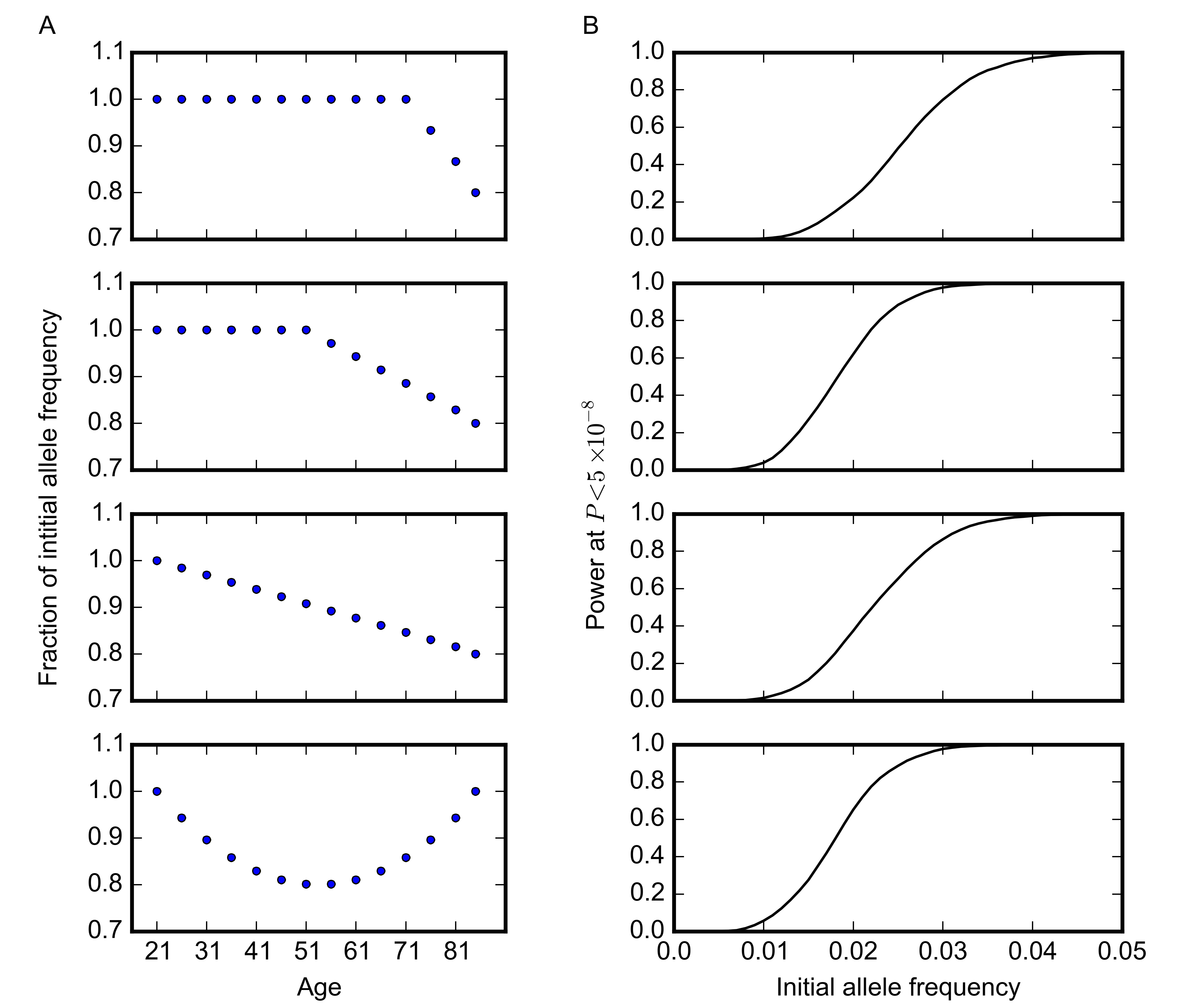

Supplement: S27 Fig — Same as Fig 1, but with 500,000 samples evenly distributed among age categories and only showing the results using models with age treated as a categorical variable. As can be seen, there should be substantial power to detect such effects even for relatively rare variants (i.e., at a couple of percent frequency in the population). (TIF) [file pbio.2002458.s027.tif]

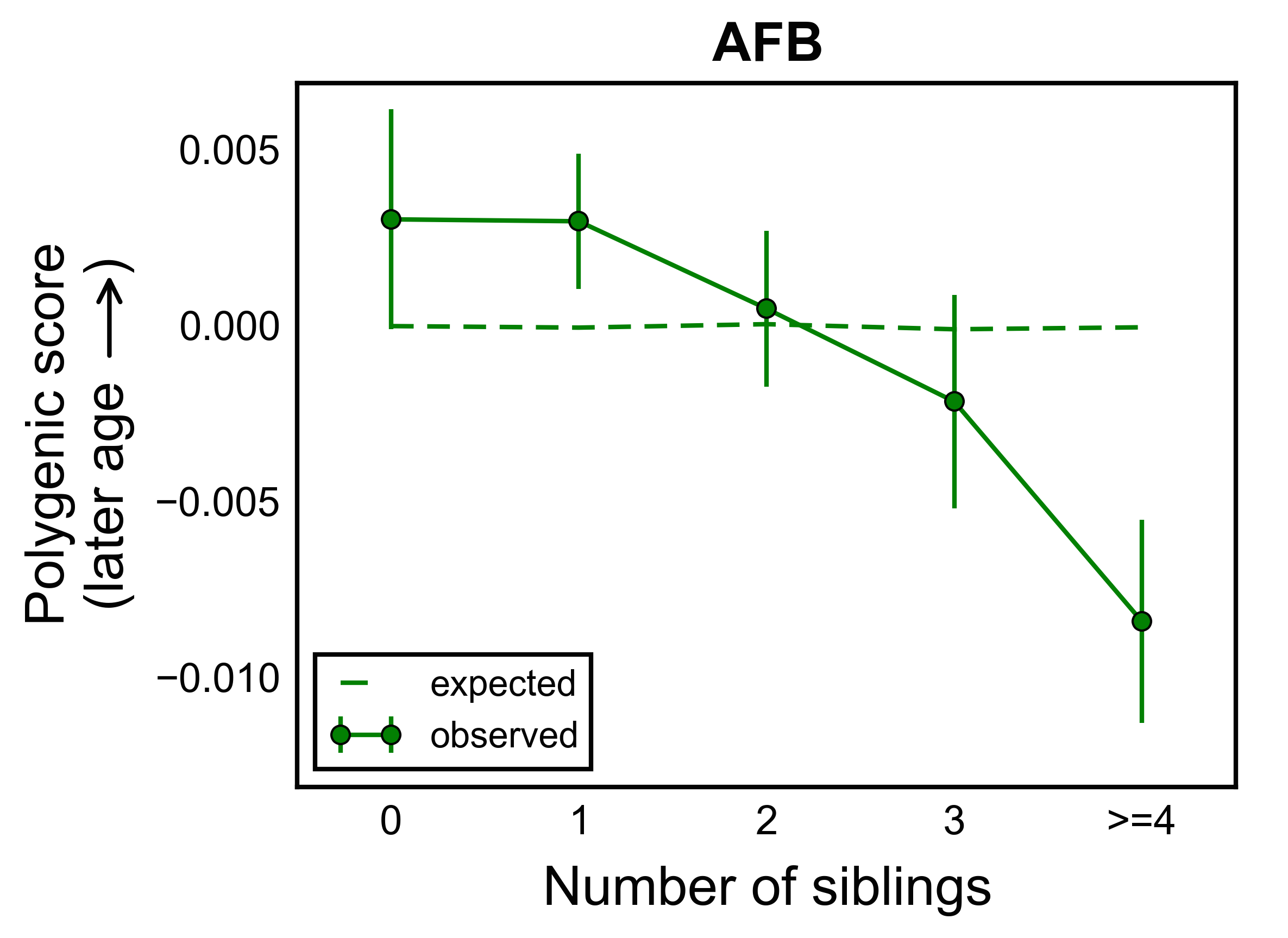

Supplement: S28 Fig — Polygenic score versus the number of siblings for 112,130 participants with mother’s age ≥ 50 years. Data points are mean scores (± 2 SE). The polygenic score was regressed on the number of siblings, accounting for the confounding batch effects, changes in ancestry, and the participant’s age, sex, year of birth, and the Townsend index (a measure of socioeconomic status). The dashed line shows the expected score based on the null model. See S2 Data for underlying data. (TIF) [file pbio.2002458.s028.tif]

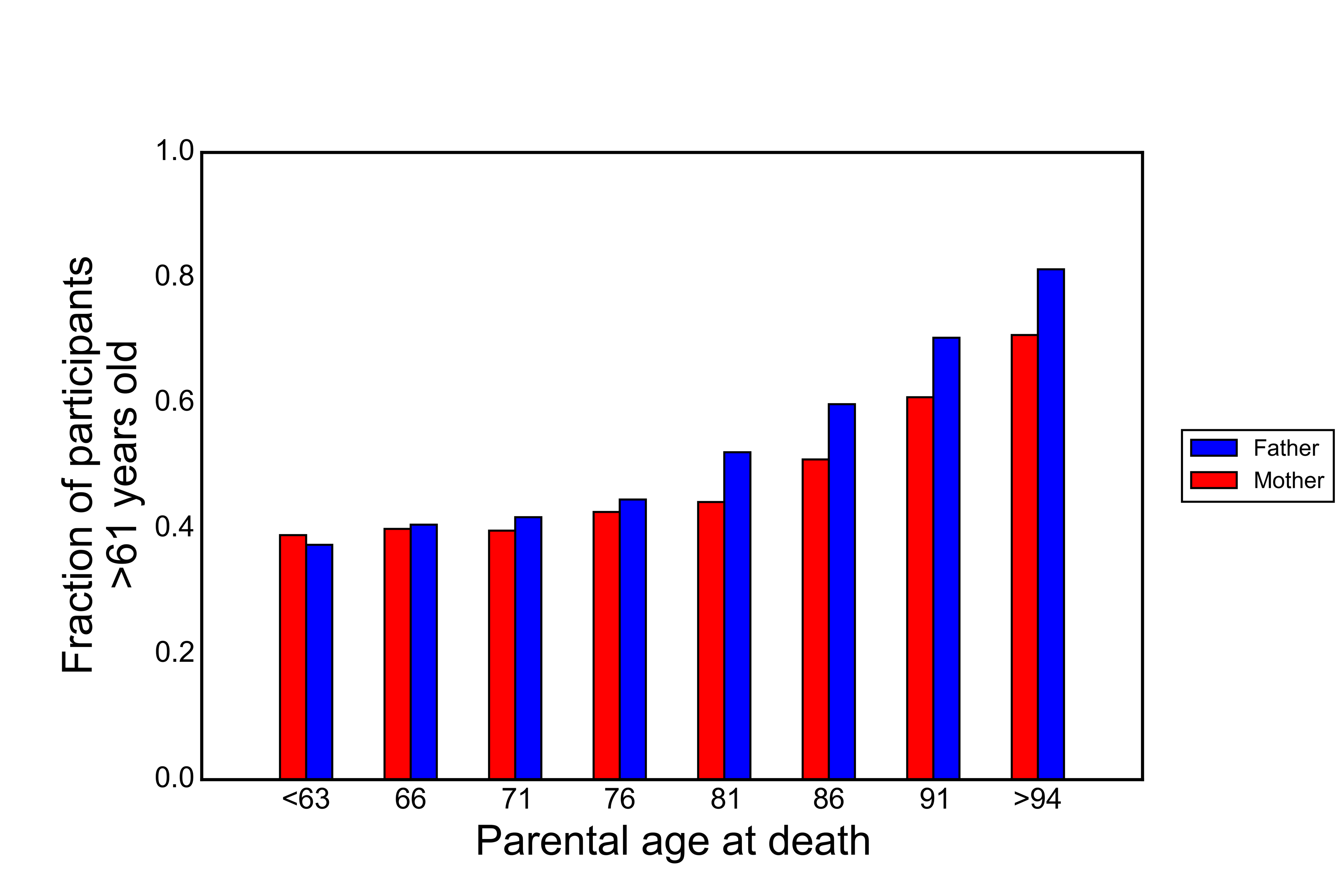

Supplement: S29 Fig — Fraction of the participants > 61 years old (last 3 age categories in S18 Fig) in each parental age bin. Assuming parents of older participants on average belong to earlier generations, older age at death categories will contain parents born earlier. See S2 Data for underlying data. (TIF) [file pbio.2002458.s029.tif]
