# Supplementary material for: Identifying genetic variants that affect viability in large cohorts
Source: PLoS Biol. 2017 Sep 5;15(9):e2002458. doi: 10.1371/journal.pbio.2002458 (PMC5584811; doi:10.1371/journal.pbio.2002458)
Supplement: S1 Table — The numbers of loci passing quality control measures are shown for each data set. (DOCX) [file pbio.2002458.s030.docx]

| Phenotype | Abbreviation | # loci in UK Biobank ^a^ | # loci in UK Biobank ^b^ | # loci in GERA |
| --- | --- | --- | --- | --- |
| Age at first birth | AFB | 10 | 10 | 10 |
| Age at natural menopause | ANM | 53 |  |  |
| Age at voice drop | AVD | 5 |  |  |
| Alzheimer’s disease ^c^ | AD | 4 |  |  |
| Any allergies | ALL | 35 |  |  |
| Asthma | ATH | 33 | 33 | 32 |
| Beighton hypermobility | BHM | 17 |  |  |
| Body mass index | BMI | 30 | 30 | 30 |
| Bone mineral density (femoral neck) | FNBMD | 18 |  |  |
| Bone mineral density (lumbar spine) | LSBMD | 20 |  |  |
| Breast size | CUP | 14 |  |  |
| Childhood ear infections | CEI | 13 |  |  |
| Chin dimples | DIMP | 52 |  |  |
| Coronary artery disease | CAD | 11 | 11 | 11 |
| Crohn’s disease | CD | 58 |  |  |
| Fasting glucose | FG | 15 |  |  |
| Height | HEIGHT | 561 |  |  |
| Hemoglobin | HB | 15 |  |  |
| High-density lipoproteins | HDL | 45 | 45 | 46 |
| Hypothyroidism | HTHY | 26 |  |  |
| Low-density lipoproteins | LDL | 39 | 40 | 40 |
| Male pattern baldness | MPB | 44 |  |  |
| Mean cell hemoglobin concentration | MCHC | 15 |  |  |
| Mean platelet volume | MPV | 29 |  |  |
| Mean red cell volume | MCV | 41 |  |  |
| Migraine | MIGR | 29 |  |  |
| Nearsightedness | NST | 159 |  |  |
| Nose size | NOSE | 10 |  |  |
| Packed red cell volume | PCV | 12 |  |  |
| Parkinson’s disease | PD | 23 |  |  |
| Photic sneeze reflex | PS | 60 |  |  |
| Platelet count | PLT | 49 |  |  |
| Puberty timing ^d^ | PT | 359 | 358 | 359 |
| Red blood cell count | RBC | 22 |  |  |
| Rheumatoid arthritis | RA | 68 |  |  |
| Schizophrenia | SCZ | 191 |  |  |
| Tonsillectomy | TS | 38 |  |  |
| Total cholesterol | TC | 49 | 51 | 51 |
| Triglycerides | TG | 28 |  |  |
| Type 2 diabetes | T2D | 11 |  |  |
| Unibrow | UB | 53 |  |  |
| Waist-hip ratio | WHR | 12 |  |  |

a: Among participants of British genetic ancestry

b: Among participants of non-British genetic ancestry

c: For AD the *APOE* locus was excluded.

d: Age at menarche associated variants were used to proximate puberty timing scores in both males and females because of the strong genetic correlation between the timing of puberty in males and females [1].

**References**

1. Day FR, Bulik-Sullivan B, Hinds DA, Finucane HK, Murabito JM, Tung JY, et al. Shared genetic aetiology of puberty timing between sexes and with health-related outcomes. Nat Commun. 2015;6:8842.
