# Supplementary material for: Identifying genetic variants that affect viability in large cohorts
Source: PLoS Biol. 2017 Sep 5;15(9):e2002458. doi: 10.1371/journal.pbio.2002458 (PMC5584811; doi:10.1371/journal.pbio.2002458)
Supplement: S3 Table — (DOCX) [file pbio.2002458.s032.docx]

| Trait | Age range | Father | | | |  | | Mother | | |  |
| --- | --- | --- | --- | --- | --- | --- | --- | --- | --- | --- | --- |
|  |  | Effect size (SE) | HR* | *P* value |  | | Effect size (SE) | | HR* | *P* value | |
| Puberty timing | $>$75  $\leq$75 | -0.0167 (0.0129)  -0.0486 (0.0102) | 0.98  0.95 | 0.20  2.1 $\times$10^-6^ |  | | -0.0174 (0.0124)  -0.0390 (0.0130) | | 0.98  0.96 | 0.16  0.0027 | |
| AFB | $>$75  $\leq$75 | -0.0453 (0.0291)  -0.0370 (0.0229) | 0.96  0.96 | 0.12  0.11 |  | | -0.0399 (0.0276)  -0.0903 (0.0290) | | 0.96  0.91 | 0.15  0.0019 | |
| ATH | $>$75  $\leq$75 | -0.0122 (0.0176)  0.0524 (0.0138) | 0.99  1.05 | 0.49  1.5 $\times$10^-4^ |  | | 0.0130 (0.0168)  0.0173 (0.0176) | | 1.01  1.02 | 0.44  0.32 | |
| BMI | $>$75  $\leq$75 | 0.1658 (0.0575)  0.2182 (0.0451) | 1.18  1.24 | 0.0040  1.3 $\times$10^-6^ |  | | 0.0659 (0.0545)  0.0961 (0.0572) | | 1.07  1.1 | 0.23  0.093 | |
| CAD | $>$75  $\leq$75 | -0.0111 (0.0286)  0.1337 (0.0224) | 0.99  1.14 | 0.70  2.6 $\times$10^-9^ |  | | 0.0647 (0.0270)  0.1150 (0.0284) | | 1.07  1.12 | 0.017  5.2 $\times$10^-5^ | |
| HDL | $>$75  $\leq$75 | -0.0304 (0.0225)  -0.0360 (0.0176) | 0.97  0.96 | 0.18  0.041 |  | | -0.0807 (0.0214)  -0.0394 (0.0224) | | 0.92  0.96 | 1.6 $\times$10^-4^  0.078 | |
| LDL | $>$75  $\leq$75 | 0.0431 (0.0227)  0.1030 (0.0177) | 1.04  1.11 | 0.058  6.2 $\times$10^-9^ |  | | 0.1183 (0.0214)  0.0487 (0.0225) | | 1.12  1.05 | 3.3 $\times$10^-8^  0.03 | |
| TC | $>$75  $\leq$75 | 0.0490 (0.0222)  0.1144 (0.0173) | 1.05  1.12 | 0.027  4.2 $\times$10^-11^ |  | | 0.1014 (0.0210)  0.0319 (0.0220) | | 1.11  1.03 | 1.4 $\times$10^-6^ 0.15 | |

*Hazard ratio.
