# Supplementary material for: Identifying genetic variants that affect viability in large cohorts
Source: PLoS Biol. 2017 Sep 5;15(9):e2002458. doi: 10.1371/journal.pbio.2002458 (PMC5584811; doi:10.1371/journal.pbio.2002458)
Supplement: S4 Table — (DOCX) [file pbio.2002458.s033.docx]

| Trait |  | Father | | |  | | Mother | | | |  | | | Meta-analysis ^a^ | | | | | | |  |
| --- | --- | --- | --- | --- | --- | --- | --- | --- | --- | --- | --- | --- | --- | --- | --- | --- | --- | --- | --- | --- | --- |
|  |  | Effect size (SE) | HR | *P* value | |  | | Effect size (SE) | HR | *P* value | |  | Effect size (SE) | | | HR | | *P* value | |  |  |
| Puberty timing |  | -0.0497 (0.0164) | 0.95 | 0.0024 | |  | | -0.0276 (0.0186) | 0.97 | 0.14 | |  | | | -0.0401 (0.0123) | | 0.96 | | 0.0011 | | |
| AFB |  | -0.0167 (0.0359) | 0.98 | 0.64 | |  | | -0.0277 (0.0409) | 0.97 | 0.50 | |  | | | -0.0215 (0.0270) | | 0.98 | | 0.42 | | |
| ATH |  | 0.0296 (0.0204) | 1.03 | 0.15 | |  | | 0.0253 (0.0228) | 1.02 | 0.27 | |  | | | 0.0277 (0.0152) | | 1.03 | | 0.068 | | |
| BMI |  | 0.2240 (0.0719) | 1.25 | 0.0018 | |  | | 0.2137 (0.0814) | 1.24 | 0.0087 | |  | | | 0.2194 (0.0539) | | 1.24 | | 4.7 $\times$10^-5^ | | |
| CAD |  | 0.0466 (0.0354) | 1.05 | 0.19 | |  | | 0.0578 (0.0399) | 1.06 | 0.15 | |  | | | 0.0516 (0.0265) | | 1.05 | | 0.051 | | |
| HDL |  | -0.0168 (0.0276) | 0.98 | 0.54 | |  | | -0.0442 (0.0313) | 0.96 | 0.16 | |  | | | -0.0288 (0.0207) | | 0.97 | | 0.16 | | |
| LDL |  | 0.0711 (0.0282) | 1.07 | 0.012 | |  | | 0.0865 (0.0320) | 1.09 | 0.0068 | |  | | | 0.0778 (0.0211) | | 1.08 | | 2.3 $\times$10^-4^ | | |
| TC |  | 0.0460 (0.0273) | 1.05 | 0.092 | |  | | 0.0738 (0.0310) | 1.08 | 0.017 | |  | | | 0.0581 (0.0205) | | 1.06 | | 0.0045 | | |

a: Combined results for fathers and mothers using inverse-variance meta-analysis on the effect sizes.
