# Supplementary material for: Identifying genetic variants that affect viability in large cohorts
Source: PLoS Biol. 2017 Sep 5;15(9):e2002458. doi: 10.1371/journal.pbio.2002458 (PMC5584811; doi:10.1371/journal.pbio.2002458)
Supplement: S5 Table — (DOCX) [file pbio.2002458.s034.docx]

| Trait |  | Beta (SE) ($\boldsymbol{\times}$ 10^4^) ^a^ | *P* value |
| --- | --- | --- | --- |
| Puberty timing |  | 3.7 (1.4) | 0.0067 |
| AFB |  | -0.69 (0.60) | 0.25 |
| ATH |  | -0.41 (1.0) | 0.70 |
| BMI |  | -0.26 (0.32) | 0.42 |
| CAD |  | -1.1 (0.62) | 0.088 |
| HDL |  | 0.042 (0.81) | 0.96 |
| LDL |  | -0.65 (0.74) | 0.38 |
| TC |  | -1.0 (0.78) | 0.18 |

a: Linear regression slope coefficient (polygenic score per year).
